# Impact of Improved Diagnosis and Treatment on Holistic CKD Burden

**DOI:** 10.1016/j.ekir.2025.05.039

**Published:** 2025-06-06

**Authors:** Navdeep Tangri, Stacey Priest, Anthony Zara, Bo Ren Long, Jieling Chen, Naveen Rao, Clélia-Elsa Froguel, Breonny Robson, Nick Guldemond, Matthew Eckelman, Ana Flavia Moura, Ralph Audehm, Fiona Adshead, Ming-hui Zhao, Christoph Wanner, Steven Chadban

**Affiliations:** 1 University of Manitoba, Winnipeg, Manitoba, Canada; 2 Department of Value & Evidence, EVERSANA, Burlington, Ontario, Canada; 3 BioPharmaceuticals Medical, AstraZeneca, Gaithersburg, Maryland, USA; 4 BioPharmaceuticals Medical, AstraZeneca, Cambridge, UK; 5 Kidney Health Australia, Melbourne, Victoria, Australia; 6 Leiden University Medical Centre, Leiden, The Netherlands; 7 Department of Civil & Environmental Engineering, Northeastern University, Boston, Massachusetts, USA; 8 Escola Bahiana de Medicina e Saúde Pública, Salvador, Brazil; 9 Department of General Practice, University of Melbourne, Melbourne, Australia; 10 Sustainable Healthcare Coalition, Devon, UK; 11 Renal Division, Peking University First Hospital Peking University Institute of Nephrology Beijing, People's Republic of China; 12 Division of Nephrology, University Hospital of Würzburg, Würzburg, Germany; 13 Royal Prince Alfred Hospital, Camperdown, Australia

**Keywords:** chronic kidney disease, disease burden, guideline directed medical therapy, health policy model, kidney replacement therapy, targeted screening

## Abstract

**Introduction:**

Chronic kidney disease (CKD) is an underdiagnosed and undertreated disease despite the availability of effective interventions. The potential clinical, economic, and environmental impacts of increased diagnosis and improved adherence to guideline-directed medical therapies (GDMTs) recommended for patients with CKD are not well-understood.

**Methods:**

Eight country populations (Australia, Brazil, China, Germany, The Netherlands, Spain, UK, and USA) were simulated for 25 years using the IMPACT CKD model to compare burdens under various diagnosis and GDMT adherence scenarios versus current practice. GDMT consisted of kidney protecting, glucose lowering, lipid lowering, as well as antihypertensive and lifestyle interventions. Patients could be treated with 1 or multiple therapies, if eligible, and no guideline changes occurred over the time horizon. Treatment effects were assumed multiplicative.

**Results:**

Scenarios with improved GDMT adherence projected cumulative decreases in dialysis, cardiovascular (CV) events, and death by −3.2% to −23.2%, −12.2% to −41.4%, and −2.3% to −9.3%, respectively, compared with current practice over 10 years. Because of delayed CKD progression, kidney replacement therapy (KRT) costs and environmental burden were also projected to decrease by −2.5% to −19.4% and −2.7% to −21.2%, respectively, compared with current practice. All treatment scenarios predicted greater improvements over 25 years, underscoring the long-term impact of CKD, and highlighting the importance of early intervention.

**Conclusion:**

Differences in projected impacts between countries are multifactorial, though they are sensitive to demographics and health care systems. Implementation of policies that lead to improved detection and treatment of CKD are urgently required across the globe to mitigate the growing burdens of CKD on patients and caregivers, health care systems, society, and our environment.

## Graphical abstract

**Figure:**
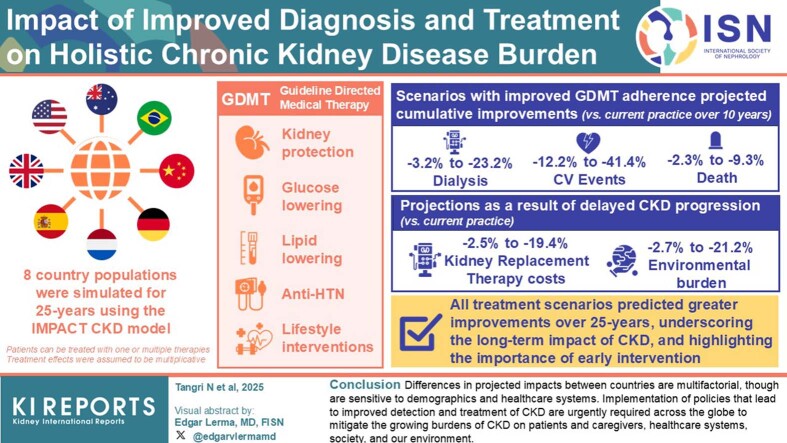

See Commentary on Page 2524

CKD is a prevalent and growing health issue, affecting approximately 13% of the world’s population; imparting negative impacts on patients, health care systems, and societies worldwide.^1^^,^^2^ Although projections for CKD have historically focused on the negative impact of CKD on patient health, quality of life, and longevity, there is the need for evaluation of CKD burden across multiple domains relevant to policymakers. Costs are high and inexorably growing, particularly for those with CKD progressing to kidney failure, where provision of KRT will contribute to a large portion of total CKD costs and an estimated 2% to 3% of the annual health care budget in high-income countries.^3^ Dialysis is the dominant form of KRT and is one of the most environmentally demanding clinical care services with high consumptions of energy, water, and single-use plastics.^4^ This is particularly problematic given the global calls to limit the environmental impact of health systems.^4^ A holistic assessment of CKD burdens, and how these burdens may be impacted by policies designed to improve rates of detection and management of CKD is required.

The IMPACT CKD microsimulation model has been validated^5^ and used to evaluate the impact of CKD across clinical, economic, societal, patient, and environmental outcomes for 8 countries globally over a 10-year time period.^6^ Across the 8 countries, the prevalence of CKD was projected to increase as much as 22.0% and patients requiring dialysis were projected to increase by 3.6% to 170.8%. The costs of CKD and KRT were projected to increase by 19.9% to 216.0%, and 20.1% to 169.5%, respectively. The overall environmental burden because of KRT was projected to increase by 11.8% to 166.2% across the 8 countries.

To mitigate growing burdens and improve outcomes for patients, urgent interventions to detect and manage CKD on a timescale commensurate with a policymaker’s standpoint are required.^7^ CKD remains underdiagnosed and undertreated^8^ despite the high burden and evidence that existing therapies can effectively delay disease progression and reduce incidence of clinical events.^9^^,^^10^ In addition, CKD is not prioritized as a noncommunicable disease, such as cancer or diabetes, by the World Health Organization; only half of the governments in countries surveyed by the Global Kidney Health Atlas recognize CKD as a health priority; and even fewer have CKD-specific policy plans.^11^ Implementation of health policies designed to improve awareness and detection of CKD, and to promote adherence to GDMTs, has great potential to mitigate the global burden.

However, knowledge gaps exist about the impact that such strategies may have on the holistic burden of CKD over time, and such data are required to inform policy decision making. Using the IMPACT CKD model, the multidimensional global burden of CKD was projected over the next 25 years with comparisons of current practice to various diagnosis and treatment scenarios.

## Methods

### Simulation Overview

The IMPACT CKD model^5^ was used to simulate 8 country populations (Australia, Brazil, China, Germany, The Netherlands, Spain, UK, and US) independently for 25 years using country-specific inputs to capture the comprehensive impact of policy interventions on CKD burdens over time. To illustrate the potential policy impacts, various diagnosis, targeted screening, and GDMT adherence scenarios were assessed and compared with the continuation of current practice. These scenarios reflect hypothetical population-level targets in screening, diagnosis, and GDMT adherence that could be achieved by implementation of recommended CKD policies. The current practice scenario was parameterized consistently with previous IMPACT CKD analyses projecting CKD burden over a 25-year time horizon; as well as calibrated and validated for each of the 8 countries, using historical country-specific epidemiological and clinical data.^5^^,^^6^

### Primary Scenarios

Primary scenarios were constructed to incrementally examine the impact of improved diagnosis, treatment, and screening and/or diagnosis plus treatment combinations versus current practice. The following primary scenario analyses were simulated: (i) increase in CKD-diagnosed population by 25%, (ii) 75% adherence to GDMTs, (iii) increase in CKD-diagnosed population by 25% combined with 75% adherence to GDMTs, and (iv) targeted CKD screening every year for patients with comorbidities (including type 2 diabetes, hypertension, heart failure, history of myocardial infarction, and history of stroke), and aged > 45 years combined with 75% adherence to GDMT.

### GDMT Considered and Targeted Level of Use

GDMT included kidney-protective, glucose- and lipid-lowering, antihypertensive, and exercise interventions. The pharmacological interventions considered in the model were: renin–angiotensin–aldosterone system inhibitors (such as angiotensin-converting enzyme inhibitors and angiotensin receptor blockers), sodium-glucose cotransporter 2 inhibitors, glucagon-like peptide-1 receptor agonists, mineralocorticoid receptor antagonists, and statins. Exercise was the sole lifestyle intervention considered in the present analysis.

Multiple kidney disease–improving global outcomes guidelines were used to inform the GDMTs, and hypothetical target use scenarios were modelled. Treatment eligibility was informed by the kidney disease–improving global outcomes guidelines.^12^, ^13^, ^14^ Target use was calculated by multiplying the guideline recommended percentage of use by the target percentage. For example, if a therapy is recommended for 90% of a subpopulation, a target GDMT use of 75% would equate to 67.5% of eligible patients receiving the therapy. Current prevalence of GDMT use was informed by various literature sources and expert opinion.^15^, ^16^, ^17^, ^18^, ^19^, ^20^ Current and target use can be found in Table 1.^12^, ^13^, ^14^, ^15^, ^16^, ^17^, ^18^, ^19^, ^20^, ^21^, ^22^, ^23^

**Table 1:** Comparison of current therapy use, and 75% of target therapy use for eligible patients based on guideline recommendations

Intervention	Comorbidity	Status of use^a^	Stage 1	Stage 2	Stage 3	Stage 4	Stage 5
RAASi^b^ (ACEi or ARB)	HTN	Current use^15^	55.9%	55.9%	55.9%	55.9%	55.9%
		Target use^12^^,^^13^	75.0%	75.0%	75.0%	75.0%	75.0%
MRA	HTN	Current use^16^	10.0%	10.0%	8.0%	5.0%	5.0%
		Target use^14^^,^^21^	11.8%	14.5%	20.0%	12.8%	5.0%
SGLT-2i	Diabetes	Current use^17^	18.2%	18.4%	15.5%	6.6%	6.4%
		Target use^c^^,^^14^	67.5%	67.5%	67.5%	49.5%	6.4%
	No diabetes	Current use^c^	10.0%	10.0%	10.0%	6.6%	6.4%
		Target use^c^^,^^14^	67.5%	67.5%	67.5%	49.5%	6.4%
GLP-1ra	Diabetes	Current use^18^	6.1%	4.2%	3.0%	2.4%	2.6%
		Target use^18^^,^^21^^,^^22^	6.1%	4.2%	3.0%	25.5%	2.6%
Lifestyle (exercise)	Any	Current use^19^	34.0%	34.0%	17.0%	11.0%	11.0%
		Target use^c^	67.5%	67.5%	67.5%	67.5%	67.5%
Statins	Any	Current use^20^	24.1%	24.1%	45.1%	53.0%	53.0%
		Target use^23^	66.0%	66.0%	66.0%	66.0%	66.0%

ACEi, angiotensin-converting enzyme inhibitor; ARB, angiotensin 2 receptor blockers; GLP-1ra, glucagon-like peptide 1 receptor agonist; HTN, hypertension; MRA, mineralocorticoid receptor antagonist; RAASi, renin-angiotensin-aldosterone system inhibitor; SGLT-2i, sodium-glucose cotransporter 2 inhibitor.

a Target use calculated by multiplying the guideline-directed use by 75%. For each sensitivity analysis (60% and 90%), target use was calculated by multiplying the guideline-directed use by the respective percentage.

b Study used to inform the current use of RAASi did not differentiate between CKD stages; therefore, an equal distribution was assumed.

c Informed by clinical expert opinion.

**Figure 1:**
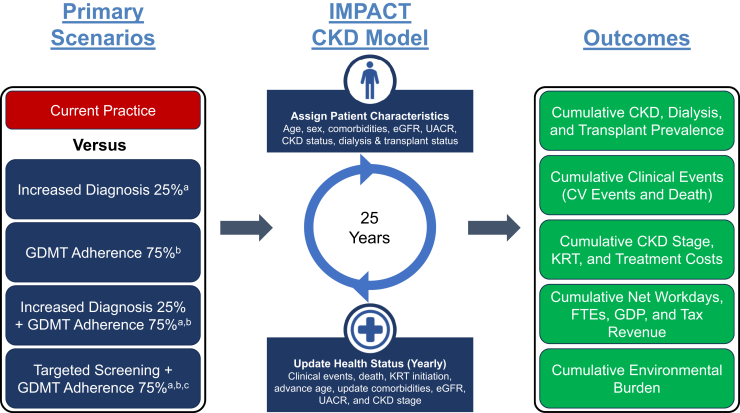
Scenario analysis framework. ^a^Only patients with partial or unlimited access to care can be diagnosed. Undiagnosed patients incur a percentage of the cost of diagnosed patients depending on the stage, as informed by data from Chen *et al.*^24^ and Spencer *et al.*^25^^b^Only patients with partial or unlimited access to care can be treated. Only diagnosed patients can be treated. Patients with CKD can be treated with multiple therapies as per guideline eligibility. A patient can be on RAASis, MRAs, SGLT-2is (or GLP-1ras), lifestyle modifications, and statins simultaneously. A patient can only be on either SGLT-2is or GLP-1ras at one time; SGLT-2is are assigned first. GLP-1ras are assigned to eligible patients not on SGLT-2is. No changes to the guidelines or detection rates are assumed over the time horizon. All therapies are associated with an improvement in eGFR decline and may reduce the occurrence of CV events or AKI events. ^c^Only patients with partial or unlimited access to care can be screened. eGFR and UACR screening are assumed to have 100% sensitivity and specificity. All screened patients undergo UACR testing. AKI, acute kidney injury; CKD, chronic kidney disease; CV, cardiovascular; eGFR, estimated glomerular filtration rate; FTE, full-time equivalent; GDMT, guideline-directed medical therapy; GDP, gross domestic product; GLP-1ra, glucagon-like peptide 1 receptor agonist; KRT, kidney replacement therapy; MRA, mineralocorticoid receptor antagonist; RAASi, renin–angiotensin-aldosterone system inhibitor; SGLT-2i, sodium-glucose cotransporter 2 inhibitor; UACR, urine albumin-creatinine ratio.

Country-specific treatment costs were identified from national public formularies, and literature sources summarized in Supplementary Tables S1 to S8. Cost inflation was not accounted for over the course of the model time horizon. Therapies had impacts on the annual rate of estimated glomerular filtration rate decline and the occurrence of CV and acute kidney injury events, which were assumed to be multiplicative and were derived from the literature (Supplementary Tables S9 and S10). Where class-specific treatment effects were unavailable, representative therapies were selected in consultation with clinical experts. Therapy effects on albuminuria were not considered for this work. An overview of the scenario analysis framework and key analysis assumptions on treatment, diagnosis, and screening can be found in Figure 1.^24^^,^^25^

### Sensitivity Scenario Analyses

Because of the interrelatedness of diagnosis, treatment, and screening, single parameter 1-way sensitivity analysis was not suitable. Instead, a set of secondary scenario analyses were conducted to examine the sensitivity of the results. The sensitivity analyses are described in the Supplementary Material.

## Results

Clinical, economic, and environmental results are presented for primary scenario analyses for each individual country (Figure 2, Figure 3, Figure 4, Figure 5, Figure 6, Supplementary Figures S1–S5). In addition, because of similarities in country characteristics, European aggregate results are shown for the 4 European countries (Germany, The Netherlands, Spain, and UK) (Figure 2, Figure 3, Figure 4, Figure 5, Figure 6, Supplementary Figures S1–S5). All CKD environmental burden and sensitivity analyses are summarized in Supplementary Figures S6 to S8 and Supplementary Figures S9 to S21, respectively. Furthermore, all cumulative outcomes at 10 and 25 years are presented in Supplementary Tables S11 to S18 and additional societal outcomes (e.g., net workdays, full-time equivalents, gross domestic product, and tax revenue) are summarized in Supplementary Tables S19 to S26.

**Figure 2:**
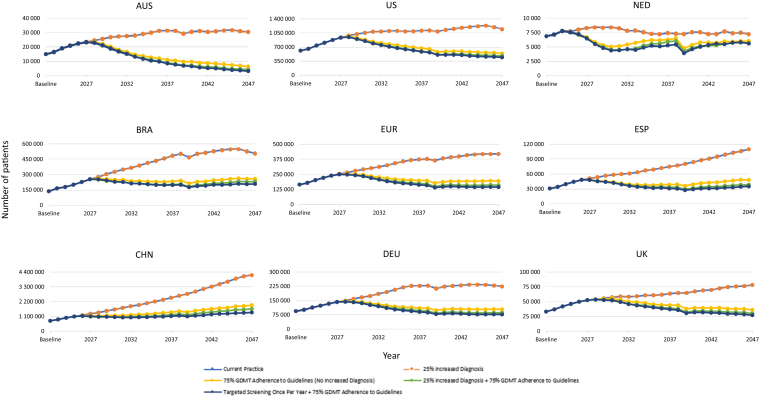
Primary scenarios—change in patients with CKD requiring dialysis over 25 years. Line traces for the “current practice” and “25% increased diagnosis” scenarios overlap because of unchanged mortality and disease progression without improved GDMT adherence. AUS, Australia; BRA, Brazil; CHN, China; CKD, chronic kidney disease; DEU, Germany; ESP, Spain; EUR, Europe; GDMT, guideline-directed medical therapy; NED, Netherlands; UK, United Kingdom; US, United States of America.

**Figure 3:**
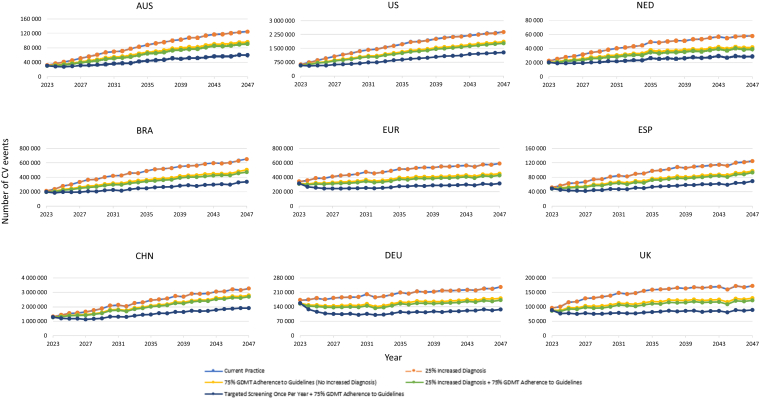
Primary scenarios—change in cardiovascular events over 25 years. Line traces for the “current practice” and “25% increased diagnosis” scenarios overlap because of unchanged mortality and disease progression without improved GDMT adherence. AUS, Australia; BRA, Brazil; CHN, China; CV, cardiovascular; DEU, Germany; ESP, Spain; EUR, Europe; GDMT, guideline-directed medical therapy; NED, Netherlands; UK, United Kingdom; US, United States of America.

**Figure 4:**
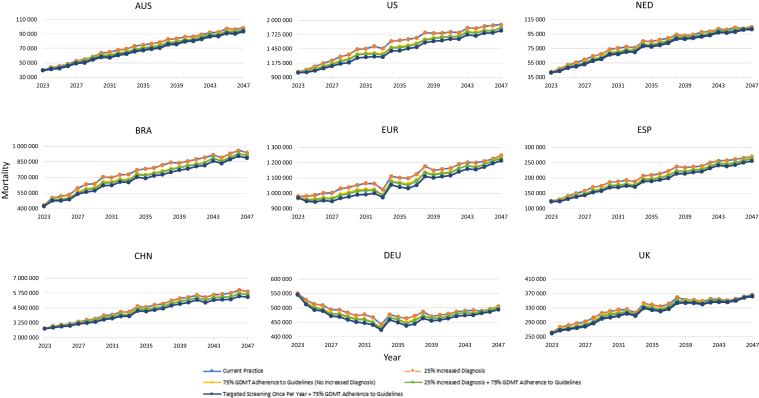
Primary scenarios—change in mortality over 25 years. Line traces for the “current practice” and “25% increased diagnosis” scenarios overlap because of unchanged mortality and disease progression without improved GDMT adherence. AUS, Australia; BRA, Brazil; CHN, China; DEU, Germany; ESP, Spain; EUR, Europe; GDMT, guideline-directed medical therapy; NED, The Netherlands; UK, United Kingdom; US, United States of America.

**Figure 5:**
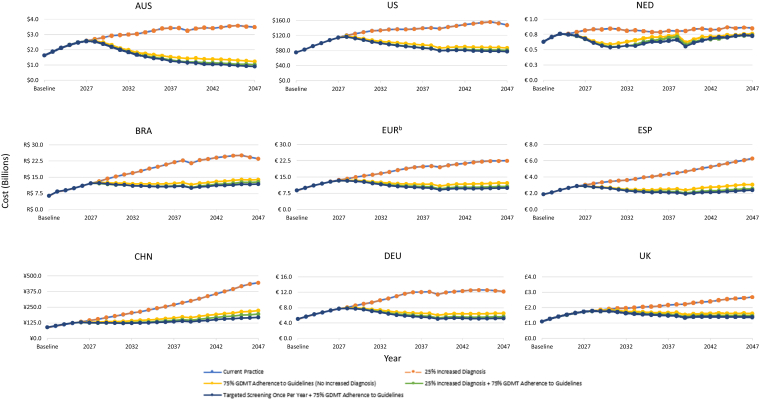
Primary scenarios—change in KRT Cost^a^ over 25 years. Line traces for the “current practice” and “25% increased diagnosis” scenarios overlap because of unchanged mortality and disease progression without improved GDMT adherence. ^a^Does not include costs associated with CKD treatment or screening. ^b^Currency conversion for UK from GBP (£) to euro (€) was performed prior to aggregation across European countries using the 2022 annual average from ECB.^26^ Values used in conversions were as follows: £1.0 = €1.173. AUS, Australia; BRA, Brazil; CHN, China; CKD, chronic kidney disease; DEU, Germany; ECB, European Central Bank; ESP, Spain; EUR, Europe; GBP, pound sterling; GDMT, guideline-directed medical therapy; KRT, kidney replacement therapy; NED, The Netherlands; UK, United Kingdom; US, United States of America.

**Figure 6:**
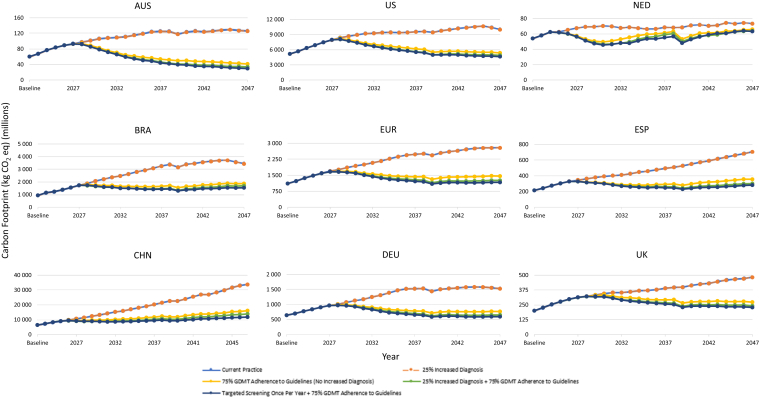
Primary scenarios—change in carbon footprint because of KRT over 25 years. Line traces for the “current practice” and “25% increased diagnosis” scenarios overlap because of unchanged mortality and disease progression without improved GDMT adherence. AUS, Australia; BRA, Brazil; CHN, China; DEU, Germany; eq, equivalent; ESP, Spain; EUR, Europe; GDMT, guideline-directed medical therapy; KRT, kidney replacement therapy; NED, The Netherlands; UK, United Kingdom; US, United States of America.

### Increase in CKD-Diagnosed Population by 25%

With increased diagnosis, dialysis prevalence (Figure 2), clinical events (Figure 3), deaths (Figure 4), CKD prevalence (Supplementary Figure S1), costs because of KRT (Figure 5),^26^ and environmental burdens because of KRT (Figure 6, Supplementary Figures S2–S3) were projected to remain essentially unchanged across all regions compared with current practice. However, non-KRT CKD costs were projected to increase slightly, by 1.9% to 2.5% over 10 years and 1.6% to 2.1% over 25 years (Supplementary Figure S4).

### Increase of GDMT Adherence to 75%

With 75% adherence to GDMTs, cumulative dialysis prevalence, CV events, and deaths were each projected to decrease by −3.2% to −19.5%, −12.2% to −21.0%, and −2.3% to −5.6% over 10 years, respectively; and −20.1% to −48.2%, −14.2% to −24.4%, and −1.8% to −5.2% over 25 years, respectively (Figure 2, Figure 3, Figure 4). Because CKD progression is delayed and survival is improved by GDMTs, cumulative early-stage (stages 1 and 2) CKD prevalence was projected to increase marginally by 0.4% to 0.6% over 10 years and 0.8% to 1.2% over 25 years across all the countries (Supplementary Figure S1, Supplementary Table S12). Similarly, late-stage (stages 3–5) CKD prevalence was projected to increase by 0.3% to 1.2% over 10 years across all the countries and the European aggregate (except for Australia, which incurred a decrease of −0.1%) and 1.0% to 3.8% over 25 years compared with current practice (Supplementary Figure S1, Supplementary Table S12).

Cumulative non-KRT CKD costs were projected to show a consistent decrease over 10 years (−0.4% to −9.6%) across all the countries and the European aggregate, except for US, which incurred a 0.5% increase compared with current practice. Over 25 years, Australia, The Netherlands, UK, and the European aggregate showed a decrease, ranging from −0.1% to −12.7%; whereas Brazil, China, Germany, and US were projected to show an increase, ranging from 0.2% to 4.5%. Spain incurred nominal changes (0.0%) (Supplementary Figure S4). Cumulative KRT costs were projected to consistently decrease by −2.5% to −15.3% over 10 years and by −14.4% to −40.6% over 25 years across all the countries and the European aggregate (Figure 5). However, cumulative total costs (including non-KRT CKD, KRT, and incremental treatment costs) were generally projected to increase across all the countries, with increases ranging from 1.8% to 17.5%, except for Australia and The Netherlands, which incurred a decrease of −2.7% and −2.2%, respectively, over 10 years. Over 25 years, the cumulative total costs in Brazil, China, UK, and US were projected to increase by 2.6% to 14.0%; whereas Australia, Germany, The Netherlands, Spain, and the European aggregate incurred a decrease ranging from −2.4% to −12.2% (Supplementary Figure S5).

Environmental burdens because of KRT were projected to consistently decrease (freshwater consumption [−2.9% to −16.7% over 10 years, −16.5% to −44.6% over 25 years; Supplementary Figure S2], fossil fuel depletion [−2.7% to −16.5% over 10 years, −13.4% to −44.6% over 25 years; Supplementary Figure S3], and carbon footprint [-2.7% to −16.6% over 10 years, −13.9% to −42.0% over 25 years; Figure 6]) across all the countries and the European aggregate compared with current practice.

The magnitude of benefits was found to be proportional to the adherence to GDMTs, meaning lower adherence to GDMT (60%) was predicted to slightly lower the reduction in cumulative clinical, economic, and environmental burdens, whereas a higher adherence to GDMT (90%) was predicted to result in higher reductions in burdens compared with the 75% adherence to GDMT scenario (Supplementary Figures S9–S21).

### Increase in CKD-Diagnosed Population by 25% and Increase of GDMT Adherence to 75%

Increasing the CKD-diagnosed population by 25% combined with GDMT adherence to 75% was projected to result in reductions in cumulative dialysis prevalence, CV events, and deaths, which were each respectively projected to decrease by −5.5% to −22.9%, −14.6% to −25.3%, and −2.8% to −6.8% over 10 years and by −25.5% to −53.5%, −17.0% to −29.0%, and −2.1% to −6.0% over 25 years compared with current practice (Figure 2, Figure 3, Figure 4). Consequent to delayed CKD progression and reduced mortality, cumulative early-stage CKD increased across all the countries and the European aggregate by 0.5% to 0.7% over 10 years and 1.1% to 1.5% over 25 years were projected to occur (Supplementary Figure S1, Supplementary Table S13). Likewise, cumulative late-stage CKD was projected to increase by 0.3% to 1.4% over 10 years except for Australia (which decreased by −0.1%); and to increase by 1.1% to 4.3% over 25 years across all the countries and the European aggregate (Supplementary Figure S1, Supplementary Table S13).

Cumulative non-KRT CKD were generally projected to increase over 10 years (0.4% to 2.8%), except for Australia, The Netherlands, and the European aggregate, which were projected to incur a decrease of −4.0%, −9.8%, and −0.1% respectively. Similarly, over 25 years, a general increase was projected, ranging from 0.9% to 6.9%, except for Australia and The Netherlands, which were projected to incur decreases of −3.5% and −13.8%, respectively (Supplementary Figure S4). Cumulative KRT costs were projected to consistently decrease across all the countries and the European aggregate over 10 years (−4.3% to −19.3%) and 25 years (−18.2% to −45.1%) (Figure 5). Cumulative total costs (including non-KRT CKD, KRT, and incremental treatment costs) shared a similar trend to non-KRT CKD which were generally projected to increase over 10 years (4.3% to 22.9%), except for Australia and The Netherlands which incurred decreases of −1.4% and −1.2%, respectively (Supplementary Figure S5). However, at 25 years, cumulative total costs in Australia, Germany, The Netherlands, Spain, and the European aggregate were projected to decrease by −1.3% to −12.5%; whereas in Brazil, China, UK, and US, they were projected to increase by 5.1% to 18.5% (Supplementary Figure S5).

Cumulative environmental burdens because of KRT were projected to consistently decrease across all the countries and the European aggregate compared with current practice (freshwater consumption [−5.0% to −21.1% over 10 years, −20.9% to −49.5% over 25 years; Supplementary Figure S2], fossil fuel depletion [−4.6% to −20.8% over 10 years, −17.0% to −46.8% over 25 years; Supplementary Figure S3], and carbon footprint [−4.6% to −20.8% over 10 years, −17.6% to −46.7% over 25 years; Figure 6]).

### Targeted CKD Screening Every Year for Patients With Comorbidities and Aged > 45 Years Coupled With an Increase of GDMT Adherence to 75%

In the scenario combining targeted screening for high-risk populations and increased GDMT adherence to 75%, cumulative dialysis prevalence was projected to be reduced over 10 years (−5.6% to −23.2%) and 25 years (−27.2% to −55.0%) across all the countries and the European aggregate compared with current practice (Figure 2). Cumulative CV events and deaths were also projected to decrease by −30.2% to −41.4% and −4.1% to −9.3% over 10 years and by −37.3% to −48.1% and −3.1% to −8.9% over 25 years, respectively (Figure 3, Figure 4). These results were further exemplified with the European aggregate projections, where the implementation of targeted screening of high-risk individuals in combination with improved adherence to GDMT projected decreases in clinical burdens for patients with CKD over 25 years (i.e., mortality [−4.6%], CV events [−43.8%], and dialysis [−43.8%]; Supplementary Table S14). Cumulative early-stage CKD prevalence was projected to increase by 1.4% to 2.2% over 10 years and 3.7% to 5.5% over 25 years across all the countries and the European aggregate compared with current practice (Supplementary Figure S1, Supplementary Table S14). Cumulative late-stage CKD was projected to generally decrease over 10 years, ranging from −0.1% to −1.9%; except for Brazil, The Netherlands, and UK, which were projected to increase slightly by 0.1% to 0.8% (Supplementary Figure S1, Supplementary Table S14). Over 25 years, the trend reverses, with a projected general increase by 0.2% to 2.6% across most countries, except for Australia and China, which were projected to incur a decrease of −3.8% and −1.4%, respectively (Supplementary Figure S1).

Cumulative KRT costs were projected to consistently decrease by −4.3% to −19.4% over 10 years and by −19.4% to −46.8% over 25 years across all the countries and the European aggregate (Figure 5). Cumulative non-KRT CKD costs were projected to generally increase by 0.8% to 4.0% over 10 years, except for Australia and The Netherlands, which decrease by −4.3% and −11.7%, respectively. Over 25 years, this trend remains, with a projected general increase of 1.5% to 6.6%, except for Australia, The Netherlands, and Spain, which decrease by −7.8%, −17.7%, and −0.5%, respectively (Supplementary Figure S4). Cumulative total costs (including non-KRT CKD, KRT, and treatment costs) were projected to consistently increase by 2.5% to 40.8% over 10 years across all the countries and the European aggregate compared with current practice. Over 25 years, the projected general increase remains, ranging from 3.9% to 40.6%, but Australia, The Netherlands, and Spain now see a projected decrease of −10.9%, −4.1%, and −3.1%, respectively (Supplementary Figure S5).

Environmental burdens because of KRT were projected to consistently decrease (freshwater consumption [−5.0% to −21.2% over 10 years, −22.2% to −51.0% over 25 years] [Supplementary Figure S2], fossil fuel depletion [−4.6% to -20.9% over 10 years, −18.0% to −49.5% over 25 years] [Supplementary Figure S3], and carbon footprint [−4.6% to −21.0% over 10 years, −18.7% over −49.5% over 25 years] [Figure 6]) across all the countries and the European aggregate compared with current practice.

Longer screening intervals (every 3 or 5 years) (Supplementary Figures S9–S21) yielded similar projection trends albeit to differing magnitudes, including lower magnitude reductions in cumulative dialysis prevalence, CV events, mortality, KRT costs, and environmental burden because of KRT compared with targeted screening every year, favoring annual screening. The opposite trend was observed for total costs and total environmental burden (because of CKD stages 1–5 and KRT), where a lower magnitude of increased costs and environmental burdens were generally projected with longer screening intervals.

## Discussion

Using the validated IMPACT CKD model, the clinical, economic, and environmental burdens were projected for current practice and compared with scenarios exploring increased diagnosis, screening, and GDMT adherence over a 25-year time period. Results provide key insights into the trajectory of the multidimensional burden of CKD if improvements in screening, diagnosis, and GDMT adherence are achieved. Specifically, the results show that increasing diagnosis without treatment is unlikely to influence total CKD or KRT prevalence, clinical events, or deaths, but does incur increases in cost and environmental burdens associated with shifting to a greater proportion of patients that are diagnosed.^24^^,^^25^ As expected, without changes to current treatment adherence, no changes in disease progression or adverse outcomes would occur; whereas the proportion of diagnosed patients which incur greater costs, increases. Contrary to the diagnosis-only scenario, all treatment scenarios predict substantial decreases in clinical events, deaths, patients requiring dialysis, KRT costs, and KRT-related environmental burden compared with current practice, with targeted screening combined with increased GDMT adherence showing the most improvements. Such major benefits incur the price of modest increases in non-KRT CKD prevalence, because improved diagnosis and management delay CKD progression and improve survival. This highlights the importance of regular screening of populations at high-risk for CKD, allowing for earlier CKD management. Furthermore, all treatment scenarios predict greater improvements in outcomes after 25 years compared with 10 years, underscoring the long-term cumulative impact of CKD, and highlighting the importance of early intervention. Although the prevalence of kidney transplantation increased over time for each country, there was no predicted difference between scenarios modelled, because organ availability is the key limiting factor for transplantation, which was assumed to increase proportionally with population growth, and was not affected across scenarios.

All scenarios, which include improved treatment adherence predict increases in CKD prevalence because of delayed disease progression to later stages and decrease in mortality. Notably, patients who would otherwise progress to later CKD stages or require KRT are kept in earlier stages, experience fewer clinical events and mortality, and are likely to experience better quality of life.^2^ Within stage 3 to 5 CKD, a shift toward CKD stage 3a–b and away from CKD stages 4 and 5 is projected across all the countries and the European aggregate from the delayed disease progression afforded by improved GDMT adherence.

Beyond the clinical benefits, as expected, total costs from overall CKD are generally projected to increase compared with current practice because of increases in the number of patients receiving care associated with improved survival. Total costs are driven by the interplay between the unit costs for KRT and treatments, where treatment costs outweigh KRT-related cost savings in many countries. Of note, Brazil is projected to have the most pronounced increases in total costs at 25 years, because the annual unit costs are relatively high for pharmacological treatments but low for KRT in Brazil, resulting in treatment costs that are not offset by the reductions in KRT costs over time. Furthermore, whereas costs are projected to increase in several countries, it is important to recognize that investments in appropriate treatment result in invaluable survival benefits for patients suffering from CKD. Although the threshold for the willingness-to-pay for therapies varies by health system, for context, the Institute for Clinical and Economic Review deems an intervention to be cost-effective below a threshold of $100,000 to $150,000 increased cost per quality-adjusted life-year (United States Dollars).^27^ In addition, the costs of treatment may be overestimated over the 25-year time period because the model projections consider no changes in CKD treatment costs, and it is possible that new generic options with reduced unit costs could become available.

Primarily driven by reductions in requirement for dialysis, KRT-related costs and environmental burden are projected to be reduced across all regions. Environmental burden among all patients with CKD vary by scenario compared with current practice; wherein improved GDMT adherence alone generally decreases environmental burden, increased diagnosis and GDMT adherence incurs marginal differences, and targeted screening with improved GDMT adherence generally increases environmental burden because of more patients being effectively diagnosed and treated leading to more diagnosed patients with improved life expectancy, which are associated with higher environmental tariffs.^24^^,^^25^

Significant societal benefits were predicted, including gained net workdays, gross domestic product contribution, full-time equivalents, and tax revenue over 10 and 25 years compared with current practice for all treatment scenarios. Given that patients with CKD and caregivers, particularly those receiving KRT, experience diminished quality of life, significant improvement can be anticipated here as well.^2^

Countries included in our study were selected for their differing population demographics and health care systems to provide a broad description of global projections. Therefore, some variations were expected between countries because of differences in key variables such as population characteristics, including age and presence of underlying comorbidities, access-to-care, health care systems, and lifestyles.^3^^,^^28^ Given the complexity of the IMPACT CKD model, single causative inputs are difficult to outline and the interpretation of data stopped at the conducted analyses. No further speculations were made when variances appeared in the patterns of outcomes between countries. Despite these variations, cumulative projections for patients requiring dialysis, clinical event occurrence, and KRT-related cost and environmental burden reductions are consistently reduced in considerable magnitude across all regions following increased diagnosis or targeted screening in combination with improved adherence to GDMT. Although the current projections could be extrapolated to countries outside of the present analysis with similarities in population demographics and health system archetypes, the validity of such generalizations is unclear. Furthermore, we acknowledge that other regions not included in the present analysis may be disproportionately affected by CKD; however, a lack of data prevents the conduct of analyses in some regions.

Improved screening, early diagnosis, and treatment adherence rely on successful policy action for CKD management to mitigate its projected holistic burden. Policy recommendations such as kidney guideline training for primary care physicians; improved coordination of care between patients, primary care physicians, and specialists; improved national CKD databases; implementation of financial incentives; strategies aimed at improving access to care; and promotion of environmentally sustainable practices in CKD management have been suggested to achieve such targets.^8^^,^^29^^,^^30^ In addition, because current financial incentives are centered around late-stage CKD, experts have noted the need to create financial incentives focused on early-stage CKD to improve early identification and treatment.^31^ Patients may be more likely to adhere to their medication with financial incentives.^32^ Based on the above-mentioned recommendations and the findings of this study, swift action toward policy implementation would be beneficial.

Key limitations of the present study are inherent to complex microsimulation models, including the need for many simplifying assumptions such as a lack of introduction of new CKD guidelines and/or CKD treatments within the time horizon and the potential underestimation of the impact of newer CKD medications creating uncertainties in the magnitude of outcomes. In addition, therapy effects on albuminuria were not considered for this work, potentially underestimating the extent of the benefits from increased GDMT compared with current practice. Moreover, though country-specific births and immigration were considered in the model to account for population growth, individuals entering the model because of birth or immigration did not have or develop CKD over the time horizon, likely underestimating the CKD prevalence. In addition, the IMPACT CKD model requires large amounts of data collected from a range of studies varying in source, methodology, population, quality, and time, affecting data certainty. Because of a lack of distribution information surrounding input parameters and the computational burden of the complex model, probabilistic sensitivity analyses were not feasible. In return, various treatment and screening scenarios have been included as sensitivity analyses. The model assumes that environmental impacts consider the continuation of current practices for care administration and does not account for potential improvements such as telemedicine. Similarly, changes to the environmental impact of CKD may be expected because efforts to decarbonize health care systems are ongoing.^33^ However, potential future changes in water, fossil fuel depletion, and carbon emission for CKD treatments were not accounted for (i.e., no discounting was applied for environmental outcomes) and the results reflect current conditions. Discounting was also not applied to the economic outcomes to keep consistency across outcomes, because some could not be discounted (e.g., clinical). Moreover, the effects of specific policy recommendations were difficult to assess and, therefore, could not be explored. The targets used for the purposes of the current analyses were validated with clinical experts but remain uncertain. Subsequently, because specific policy recommendations could not be explored, policies themselves could not be costed. Furthermore, though other lifestyle interventions may be prescribed to patients, only exercise was considered in the model because of the scarcity of data on the impact of other interventions on estimated glomerular filtration rate decline and clinical events. It was also assumed that exercise did not incur any costs because it would likely not cost health care systems. Altogether, many simplifying assumptions were made because of the lack of data on both the disease and the treatment effects on the disease. Lastly, when interpreting the results figures, though country curves may follow similar trends, it is important to note the variations in the scales of the y-axes because curvature magnitudes can differ.

The multidimensional population-level burden of CKD was projected comparing several diagnosis and treatment scenarios versus current practice over 25 years using the IMPACT CKD model to provide insights into the benefits of early diagnosis and improved GDMT adherence. Overall, improved diagnosis or targeted screening combined with improved treatment is projected to delay disease progression, resulting in fewer clinical events and deaths, as well as reductions in KRT prevalence and subsequent KRT-related costs and environmental burden across all individual countries and Europe. The results from the present study encourage urgent strategic action through policy implementation that would lead to improved detection and treatment of patients with CKD to mitigate the growing burdens of CKD on patients, caregivers, health care systems, and the environment alike.

## Disclosure

SP, AZ, and BRL are employees of EVERSANA; AstraZeneca contracted EVERSANA to complete this study. JC, NR, and C-EF are employees of AstraZeneca. JC is a minor stockholder of AstraZeneca. NT, BR, NG, ME, AFM, RA, FA, M-hZ, CW, and SC received honoraria for participating in consultant meetings for AstraZeneca. NT has also received grants or contracts from CIHR, NIH, KFOC, Bayer, AstraZeneca, Boehringer Ingelheim, Janssen Pharmaceuticals, Research Manitoba, Otsuka Pharmaceutical Co, Ltd, Tricida Inc., and Eli Lilly and Company. NT has received consulting fees and/or honoraria from Bayer, Boehringer Ingelheim, Janssen Pharmaceuticals, GSK, Otsuka Pharmaceutical Co, Ltd, ProKidney, Roche, Vera, Tricida Inc., and Eli Lilly and Company. NT has received equity from ProKidney and Klinrisk. NT has a patent pending for a microfluidic device for point of care detection of urine albumin. NT has a leadership or fiduciary role in AstraZeneca, Janssen, BI-Lilly, Otsuka, and the National Kidney Foundation. NT is a stockholder of ClinPredict, Klinrisk, Quanta, Marizyme, Mesentech, Renibus Therapeutics, Pulsedata, and Tricida. BR is employed as General Manager at Kidney Health Australia and was also involved in the development of CKD and CVD guidelines in Australia. BR’s institution has received honoraria from Boehringer Ingelheim for participation in panel discussions. BR’s organization has received funding grants from AstraZeneca, Boehringer Ingelheim, Baxter Healthcare, GSK, and CSL Seqirus. AFM has attended WCN’24 as a presenting author for AstraZeneca for a related topic. RA has also received honoraria from Aspen Pharmacare, Seqirus, Boehringer Ingelheim, Novartis, Novo Nordisk, Servier, Eli Lilly, BMS and Menarini. MHZ has also received honoraria and/or advisory fees from GSK, Roche, SanReno, Novartis and Beigene. CW has also received honoraria from Amgen, Amicus, Alexion, Bayer, Boehringer Ingelheim, CSL Vifor, Chiesi, GSK, Idorsia, Eli Lilly, MSD, Novartis, Novo Nordisk, Sanofi, and VeraTX. CW’s institution has received grants or contracts from Sanofi. SC has received consulting fees from Alexion for participation in a trial steering committee. SC has also received honoraria from Boehringer Ingelheim, GSK, Astellas, Novartis, and NovoNordisk.

## Supplementary Material

Supplementary File (PDF) **Sensitivity Analyses. Supplementary References. Figure S1.** Primary scenarios—change in CKD prevalence (excluding KRT) over 25 years. **Figure S2.** Primary scenarios—change in freshwater consumption because of KRT over 25 years. **Figure S3.** Primary scenarios—change in fossil fuel depletion because of KRT over 25 years. **Figure S4.** Primary scenarios—change in non-KRT CKD Cost over 25 years. **Figure S5.** Primary scenarios—change in total cost (including non-KRT CKD cost, KRT cost, and treatment cost) over 25 years. **Figure S6.** Primary scenarios—change in freshwater consumption because of all CKD over 25 years. **Figure S7.** Primary scenarios—change in fossil fuel depletion because of all CKD over 25 years. **Figure S8.** Primary scenarios—change in carbon footprint because of all CKD over 25 years. **Figure S9.** Sensitivity scenarios—change in patients with CKD requiring dialysis over 25 years. **Figure S10.** Sensitivity scenarios—change in cardiovascular events over 25 years. **Figure S11.** Sensitivity scenarios—change in mortality over 25 years. **Figure S12.** Sensitivity scenarios—change in CKD prevalence over 25 years. **Figure S13.** Sensitivity scenarios—change in non-KRT CKD cost over 25 years. **Figure S14.** Sensitivity scenarios—change in KRT cost over 25 years. **Figure S15.** Sensitivity scenarios—change in total cost over 25 years. **Figure S16.** Sensitivity scenarios—change in freshwater consumption because of all CKD over 25 years. **Figure S17.** Sensitivity scenarios—change in fossil fuel depletion because of all CKD over 25 years. **Figure S18.** Sensitivity scenarios—change in carbon footprint because of all CKD over 25 years. **Figure S19.** Sensitivity scenarios—change in freshwater consumption because of KRT over 25 years. **Figure S20.** Sensitivity scenarios—change in fossil fuel depletion because of KRT over 25 years. **Figure S21.** Sensitivity scenarios—change in carbon footprint because of KRT over 25 years. **Table S1.** Country-specific CKD therapy costs∗ for Australia. **Table S2.** Country-specific CKD therapy costs∗ for Brazil. **Table S3.** Country-specific CKD therapy costs∗ for China. **Table S4.** Country-specific CKD therapy costs∗ for Germany. **Table S5.** Country-specific CKD therapy costs∗ for The Netherlands. **Table S6.** Country-specific CKD therapy costs∗ for Spain. **Table S7.** Country-specific CKD therapy costs∗ for UK. **Table S8.** Country-specific CKD therapy costs∗ for US. **Table S9.** Impact of individual GDMT therapies on the annual rate of eGFR decline. **Table S10.** Impact of individual GDMT therapies on the annual rate of clinical events. **Table S11.** CKD burden over 10 and 25 years following a 25% increase of the diagnosed population compared with current practice. **Table S12.** CKD burden over 10 and 25 years following an increase of GDMT adherence to 75% compared with current practice. **Table S13.** CKD burden over 10 and 25 years following a 25% increase of diagnosed population and an increase of GDMT adherence to 75% compared with current practice. **Table S14.** CKD burden over 10 and 25 years following annual targeted screening of high-risk population and an increase to GDMT adherence to 75% compared wih current practice. **Table S15.** CKD burden over 10 and 25 years following an increase to 60% adherence to GDMT compared with current practice. **Table S16.** CKD burden over 10 and 25 years following an increase to 90% adherence to GDMT compared with current practice. **Table S17.** CKD burden over 10 and 25 years following targeted screening every 3 years for high-risk population and a 75% adherence to GDMT compared with current practice. **Table S18.** CKD burden over 10 and 25 years following targeted screening every 5 years for high-risk population and a 75% adherence to GDMT compared with current practice. **Table S19.** Societal CKD burden over 10 and 25 years following a 25% increase of the diagnosed population compared with current practice. **Table S20.** Societal CKD burden over 10 and 25 years following an increase of GDMT adherence to 75% compared with current practice. **Table S21.** Societal CKD burden over 10 and 25 years following a 25% increase of diagnosed population and an increase to GDMT adherence to 75% compared with current practice. **Table S22.** Societal CKD burden over 10 and 25 years following annual targeted screening of high-risk population and an increase to GDMT adherence to 75% compared with current practice. **Table S23.** Societal CKD burden over 10 and 25 years following an increase to 60% adherence to GDMT compared with current practice. **Table S24.** Societal CKD burden over 10 and 25 years following an increase to 90% adherence to GDMT compared with current practice. **Table S25.** Societal CKD burden over 10 and 25 years following targeted screening every 3 years for high-risk populations and a 75% adherence to GDMT compared with current practice. **Table S26.** Societal CKD burden over 10 and 25 years following targeted screening every 5 years for high-risk populations and a 75% adherence to GDMT compared with current practice. CHEERS Checklist.

## Footnotes

Supplementary File (PDF)
**Sensitivity Analyses**. **Supplementary References**. **Figure S1.** Primary scenarios—change in CKD prevalence (excluding KRT) over 25 years. **Figure S2.** Primary scenarios—change in freshwater consumption because of KRT over 25 years. **Figure S3.** Primary scenarios—change in fossil fuel depletion because of KRT over 25 years. **Figure S4.** Primary scenarios—change in non-KRT CKD Cost over 25 years. **Figure S5.** Primary scenarios—change in total cost (including non-KRT CKD cost, KRT cost, and treatment cost) over 25 years. **Figure S6.** Primary scenarios—change in freshwater consumption because of all CKD over 25 years. **Figure S7.** Primary scenarios—change in fossil fuel depletion because of all CKD over 25 years. **Figure S8.** Primary scenarios—change in carbon footprint because of all CKD over 25 years. **Figure S9.** Sensitivity scenarios—change in patients with CKD requiring dialysis over 25 years. **Figure S10.** Sensitivity scenarios—change in cardiovascular events over 25 years. **Figure S11.** Sensitivity scenarios—change in mortality over 25 years. **Figure S12.** Sensitivity scenarios—change in CKD prevalence over 25 years. **Figure S13.** Sensitivity scenarios—change in non-KRT CKD cost over 25 years. **Figure S14.** Sensitivity scenarios—change in KRT cost over 25 years. **Figure S15.** Sensitivity scenarios—change in total cost over 25 years. **Figure S16.** Sensitivity scenarios—change in freshwater consumption because of all CKD over 25 years. **Figure S17.** Sensitivity scenarios—change in fossil fuel depletion because of all CKD over 25 years. **Figure S18.** Sensitivity scenarios—change in carbon footprint because of all CKD over 25 years. **Figure S19.** Sensitivity scenarios—change in freshwater consumption because of KRT over 25 years. **Figure S20.** Sensitivity scenarios—change in fossil fuel depletion because of KRT over 25 years. **Figure S21.** Sensitivity scenarios—change in carbon footprint because of KRT over 25 years. **Table S1.** Country-specific CKD therapy costs∗ for Australia. **Table S2.** Country-specific CKD therapy costs∗ for Brazil. **Table S3.** Country-specific CKD therapy costs∗ for China. **Table S4.** Country-specific CKD therapy costs∗ for Germany. **Table S5.** Country-specific CKD therapy costs∗ for The Netherlands. **Table S6.** Country-specific CKD therapy costs∗ for Spain. **Table S7.** Country-specific CKD therapy costs∗ for UK. **Table S8.** Country-specific CKD therapy costs∗ for US. **Table S9.** Impact of individual GDMT therapies on the annual rate of eGFR decline. **Table S10.** Impact of individual GDMT therapies on the annual rate of clinical events. **Table S11.** CKD burden over 10 and 25 years following a 25% increase of the diagnosed population compared with current practice. **Table S12.** CKD burden over 10 and 25 years following an increase of GDMT adherence to 75% compared with current practice. **Table S13.** CKD burden over 10 and 25 years following a 25% increase of diagnosed population and an increase of GDMT adherence to 75% compared with current practice. **Table S14.** CKD burden over 10 and 25 years following annual targeted screening of high-risk population and an increase to GDMT adherence to 75% compared wih current practice. **Table S15.** CKD burden over 10 and 25 years following an increase to 60% adherence to GDMT compared with current practice. **Table S16.** CKD burden over 10 and 25 years following an increase to 90% adherence to GDMT compared with current practice. **Table S17.** CKD burden over 10 and 25 years following targeted screening every 3 years for high-risk population and a 75% adherence to GDMT compared with current practice. **Table S18.** CKD burden over 10 and 25 years following targeted screening every 5 years for high-risk population and a 75% adherence to GDMT compared with current practice. **Table S19.** Societal CKD burden over 10 and 25 years following a 25% increase of the diagnosed population compared with current practice. **Table S20.** Societal CKD burden over 10 and 25 years following an increase of GDMT adherence to 75% compared with current practice. **Table S21.** Societal CKD burden over 10 and 25 years following a 25% increase of diagnosed population and an increase to GDMT adherence to 75% compared with current practice. **Table S22.** Societal CKD burden over 10 and 25 years following annual targeted screening of high-risk population and an increase to GDMT adherence to 75% compared with current practice. **Table S23.** Societal CKD burden over 10 and 25 years following an increase to 60% adherence to GDMT compared with current practice. **Table S24.** Societal CKD burden over 10 and 25 years following an increase to 90% adherence to GDMT compared with current practice. **Table S25.** Societal CKD burden over 10 and 25 years following targeted screening every 3 years for high-risk populations and a 75% adherence to GDMT compared with current practice. **Table S26.** Societal CKD burden over 10 and 25 years following targeted screening every 5 years for high-risk populations and a 75% adherence to GDMT compared with current practice. CHEERS Checklist.
