# Supplementary material for: Impact of Improved Diagnosis and Treatment on Holistic CKD Burden
Source: Kidney Int Rep. 2025 Jun 6;10(8):2608–20. doi: 10.1016/j.ekir.2025.05.039 (PMC12348058; doi:10.1016/j.ekir.2025.05.039)
Supplement: Supplementary File (PDF) — Sensitivity Analyses. Supplementary References. Figure S1. Primary scenarios—change in CKD prevalence (excluding KRT) over 25 years. Figure S2. Primary scenarios—change in freshwater consumption because of KRT over 25 years. Figure S3. Primary scenarios—change in fossil fuel depletion because of KRT over 25 years. Figure S4. Primary scenarios—change in non-KRT CKD Cost over 25 years. Figure S5. Primary scenarios—change in total cost (including non-KRT CKD cost, KRT cost, and treatment cost) over 25 years. Figure S6. Primary scenarios—change in freshwater consumption because of all CKD over 25 years. Figure S7. Primary scenarios—change in fossil fuel depletion because of all CKD over 25 years. Figure S8. Primary scenarios—change in carbon footprint because of all CKD over 25 years. Figure S9. Sensitivity scenarios—change in patients with CKD requiring dialysis over 25 years. Figure S10. Sensitivity scenarios—change in cardiovascular events over 25 years. Figure S11. Sensitivity scenarios—change in mortality over 25 years. Figure S12. Sensitivity scenarios—change in CKD prevalence over 25 years. Figure S13. Sensitivity scenarios—change in non-KRT CKD cost over 25 years. Figure S14. Sensitivity scenarios—change in KRT cost over 25 years. Figure S15. Sensitivity scenarios—change in total cost over 25 years. Figure S16. Sensitivity scenarios—change in freshwater consumption because of all CKD over 25 years. Figure S17. Sensitivity scenarios—change in fossil fuel depletion because of all CKD over 25 years. Figure S18. Sensitivity scenarios—change in carbon footprint because of all CKD over 25 years. Figure S19. Sensitivity scenarios—change in freshwater consumption because of KRT over 25 years. Figure S20. Sensitivity scenarios—change in fossil fuel depletion because of KRT over 25 years. Figure S21. Sensitivity scenarios—change in carbon footprint because of KRT over 25 years. Table S1. Country-specific CKD therapy costs∗ for Australia. Table S2. Country-specific CKD the [file mmc1.pdf]

Supplementary Materials

Table of Contents

Sensitivity analyses. 4

Supplementary Table S1. Country-specific CKD therapy costs* for Australia 4

Supplementary Table S2. Country-specific CKD therapy costs* for Brazil. 5

Supplementary Table S3. Country-specific CKD therapy costs* for China. 5

Supplementary Table S4. Country-specific CKD therapy costs* for Germany..6

Supplementary Table S5. Country-specific CKD therapy costs* for Netherlands. 6

Supplementary Table S6. Country-specific CKD therapy costs* for Spain. 7

Supplementary Table S7. Country-specific CKD therapy costs* for UK. 7

Supplementary Table S8. Country-specific CKD therapy costs* for US...8

Supplementary Table S9. Impact of individual GDMT therapies on the annual rate of eGFR decline. 9

Supplementary Table S10. Impact of individual GDMT therapies on the annual rate of clinical events...........9

Supplementary Table S11. CKD burden over 10 and 25 years following a 25% increase of the diagnosed population compared to current practice. 11

Supplementary Table S12. CKD burden over 10 and 25 years following an increase of GDMT adherence to 75% compared to current practice. 15

Supplementary Table S13. CKD burden over 10 and 25 years following a 25% increase of diagnosed population and an increase to GDMT adherence to 75% compared to current practice. 20

Supplementary Table S14. CKD burden over 10 and 25 years following annual targeted screening of high-risk population and an increase to GDMT adherence to 75% compared to current practice. 26

Supplementary Table S15. CKD burden over 10 and 25 years following an increase to 60% adherence to GDMT compared to current practice. 32

Supplementary Table S16. CKD burden over 10 and 25 years following an increase to 90% adherence to GDMT compared to current practice. 38

Supplementary Table S17. CKD burden over 10 and 25 years following targeted screening every 3 years for high-risk population and a 75% adherence to GDMT compared to current practice. 44

Supplementary Table S18. CKD burden over 10 and 25 years following targeted screening every 5 years for high-risk population and a 75% adherence to GDMT compared to current practice. 52

Supplementary Table S19. Societal CKD burden over 10 and 25 years following a 25% increase of the diagnosed population compared to current practice. 60

Supplementary Table S20. Societal CKD burden over 10 and 25 years following an increase of GDMT adherence to 75% compared to current practice...62

Supplementary Table S21. Societal CKD burden over 10 and 25 years following a 25% increase of diagnosed population and an increase to GDMT adherence to 75% compared to current practice. 64

Supplementary Table S22. Societal CKD burden over 10 and 25 years following annual targeted screening of high-risk population and an increase to GDMT adherence to 75% compared to current practice. 67

Supplementary Table S23. Societal CKD burden over 10 and 25 years following an increase to 60% adherence to GDMT compared to current practice. 70

Supplementary Table S24. Societal CKD burden over 10 and 25 years following an increase to 90% adherence to GDMT compared to current practice. 72

Supplementary Table S25. Societal CKD burden over 10 and 25 years following targeted screening every 3 years for high-risk populations and a 75% adherence to GDMT compared to current practice. 74

Supplementary Table S26. Societal CKD burden over 10 and 25 years following targeted screening every 5 years for high-risk populations and a 75% adherence to GDMT compared to current practice. 78

Supplementary Figure S1. Primary Scenarios - Change in CKD Prevalence (excl. KRT) over 25 years. 82

Supplementary Figure S2. Primary Scenarios - Change in Freshwater Consumption due to KRT over 25 years 83

Supplementary Figure S3. Primary Scenarios - Change in Fossil Fuel Depletion due to KRT over 25 years. .84

Supplementary Figure S4. Primary Scenarios - Change in non-KRT CKD Cost over 25 years. 85

Supplementary Figure S5. Primary Scenarios - Change in Total Cost (Incl. non-KRT CKD Cost, KRT Cost, Treatment Cost) over 25 years. 86

Supplementary Figure S6. Primary Scenarios - Change in Freshwater Consumption due to all CKD over 25 years. 87

Supplementary Figure S7. Primary Scenarios - Change in Fossil Fuel Depletion due to all CKD over 25 years. 88

Supplementary Figure S8. Primary Scenarios - Change in Carbon Footprint due to all CKD over 25 years. .89

Supplementary Figure S9. Sensitivity Scenarios - Change in CKD Patients requiring dialysis over 25 years. 90

Supplementary Figure S10. Sensitivity Scenarios - Change in Cardiovascular Events over 25 years. 91

Supplementary Figure S11. Sensitivity Scenarios - Change in Mortality over 25 years. 92

Supplementary Figure S12. Sensitivity Scenarios - Change in CKD Prevalence over 25 years. 93

Supplementary Figure S13. Sensitivity Scenarios - Change in non-KRT CKD Cost over 25 years. 94

Supplementary Figure S14. Sensitivity Scenarios - Change in KRT Cost over 25 years. 95

Supplementary Figure S15. Sensitivity Scenarios - Change in Total Cost over 25 years. 96

Supplementary Figure S16. Sensitivity Scenarios - Change in Freshwater Consumption due to all CKD over 25 years. 97

Supplementary Figure S17. Sensitivity Scenarios - Change in Fossil Fuel Depletion due to all CKD over 25 years. 98

Supplementary Figure S18. Sensitivity Scenarios - Change in Carbon Footprint due to all CKD over 25 years. 99

Supplementary Figure S19. Sensitivity Scenarios - Change in Freshwater Consumption due to KRT over 25 years. 100

Supplementary Figure S20. Sensitivity Scenarios - Change in Fossil Fuel Depletion due to KRT over 25 years. 101

Supplementary Figure S21. Sensitivity Scenarios - Change in Carbon Footprint due to KRT over 25 years. 102

Supplementary References 103

CHEERS Checklist..........106

Sensitivity analyses

In addition to the primary analyses that were conducted, the following sensitivity analyses were simulated: 1) 60% adherence to guideline-directed medical therapy (GDMT), 2) 90% adherence to GDMT, 3) targeted chronic kidney disease (CKD) screening every 3 years for patients with comorbidities (including type 2 diabetes [T2D], hypertension [HTN], heart failure [HF], history of myocardial infarction [MI], and history of stroke), and over the age of 45 years combined with 75% adherence to GDMT, 4) targeted CKD screening every 5 years for patients with comorbidities (including T2D, HTN, HF, history of MI, and history of stroke), and over the age of 45 years combined with 75% adherence to GDMT. Additionally, to explore the long-term impact of improved diagnosis, screening, and GDMT adherence, the time horizon was also extended to 25 years.

Supplementary Tables

Supplementary Table S1. Country-specific CKD therapy costs* for Australia.

Intervention	Pharmacological Therapy Costed	Details	Unit cost- AUD	Annual cost-AUD	Reference	Reference description
SGLT-2i	Dapagliflozin	10 mg OD	57.82	754.24	PBS.gov[S1]	Formulary
RASi	Irbesartan	75-300 mg OD[a]	16.05	195.41	PBS.gov[S2]	Formulary
MRA	Finerenone	20 mg OD	84.04	1096.27	PBS.gov[S3]	Formulary
GLP-1ra	Semaglutide	0.5/1.0 mg injection QW	31.6	1643.2	PBS.gov[S4]	Formulary
Statin	Simvastatin plus ezetimibe	20 mg + 10 mg OD	23.52	286.36	PBS.gov[S5]	Formulary

*Lifestyle intervention was assumed to have a cost of $0.

[a]Average between 75 mg, 150 mg, and 300 mg unit costs given range of dosing used in trial that informed eGFR treatment benefit.[S6]

Abbreviations: ACEi = angiotensin-converting enzyme inhibitor; ARB = angiotensin 2 receptor blockers; AUD = Australian dollar; CKD = chronic kidney disease; eGFR = estimated glomerular filtration rate; GLP-1ra = glucagon-like peptide 1 receptor agonist; MRA = mineralocorticoid receptor antagonist; OD = once daily; PBS = Pharmaceutical Benefits Scheme; QW = once weekly; RASi = renin-angiotensin system inhibitor; SGLT-2i = sodium-glucose cotransporter 2 inhibitor.

Supplementary Table S2. Country-specific CKD therapy costs* for Brazil.

Intervention	Pharmacological Therapy Costed	Details	Unit cost- BRL	Annual cost - BRL	Reference	Reference description
SGLT-2i	Dapagliflozin	10 mg OD	117.78	1433.97	CMED medicine prices[S7]	Formulary
RASi	Irbesartan	150-300 mg OD[a]	129.41	1575.75	CMED medicine prices[S7]	Formulary
MRA	Finerenone	20 mg OD	166.29	2169.19	CMED medicine prices[S7]	Formulary
GLP-1ra	Semaglutide	14 mg OD or 0.5/1.0 mg injection QW[b]	1065.03 (14 mg); 994.03 (0.5/1.0 mg)	12438.41	CMED medicine prices[S7]	Formulary
Statin	Simvastatin plus ezetimibe	20 mg + 10 mg OD	147.60	1925.39	CMED medicine prices[S7]	Formulary

[*]Lifestyle intervention was assumed to have a cost of R$0.
[a]Average between 150 mg, and 300 mg unit costs given range of dosing used in trial that informed eGFR treatment benefit.[S6]
[b]Weighted average performed with the patient counts from studies that informed eGFR treatment effect (14 mg OD, N = 1591; 0.5 mg QW, N = 826 patients; 1 mg QW, N = 822 patients).[S8]
Abbreviations: ACEi = angiotensin-converting enzyme inhibitor; ARB = angiotensin 2 receptor blockers; BRL = Brazilian Real; CKD = chronic kidney disease; CMED = Brazilian Medicines Market Regulation Chamber; eGFR = estimated glomerular filtration rate; GLP-1ra = glucagon-like peptide 1 receptor agonist; MRA = mineralocorticoid receptor antagonist; OD = once daily; QW = once weekly; RASi = renin-angiotensin system inhibitor; SGLT-2i = sodium-glucose cotransporter 2 inhibitor.

Supplementary Table S3. Country-specific CKD therapy costs* for China.

Intervention	Pharmacological Therapy Costed	Details	Unit cost- CNY	Annual cost- CNY	Reference	Reference description
SGLT-2i	Dapagliflozin	10 mg OD	-	7128.38	Literature[S9]	CEA of patients with T2D
RASi	Irbesartan	75-300 mg OD[a]	-	1572.46	Literature[S10,11]	Cost comparison of antihypertensive drugs in China vs. the US/CEA of patients with T2D, hypertension and nephropathy
MRA	Finerenone	20 mg OD	13.94	5091.59	Other[S12]	CEA treating T2D and CKD
GLP-1ra	Semaglutide	14 mg OD or 0.5/1.0 mg injection QW[b]	NR (14 mg); 478.8 (0.5/1.0 mg)	12448.80	Literature[S13,14]	CEA of patients with T2D
Statin	Rosuvastatin (proxy for simvastatin) plus ezetimibe	10 mg + 10 mg OD	27.19	9931.15	Literature[S15]	CEA of patients with CVD

[*]Lifestyle intervention was assumed to have a cost of ¥0.
[a]Average between 75 mg and 300 mg unit costs given range of dosing used in trial that informed eGFR treatment benefit.[S6]
[b]Weighted average performed with the patient counts from studies that informed eGFR treatment effect (14 mg OD, N = 1591; 0.5 mg QW, N = 826 patients; 1 mg QW, N = 822 patients).[S8]
Abbreviations: ACEi = angiotensin-converting enzyme inhibitor; ARB = angiotensin 2 receptor blockers; CEA = cost-effectiveness analysis; CKD = chronic kidney disease; CNY = Chinese Yuan; CVD = cardiovascular disease; eGFR = estimated glomerular filtration rate; GLP-1ra = glucagon-like peptide 1 receptor agonist; MRA = mineralocorticoid receptor antagonist; NR = not reported; OD = once daily; QW = once weekly; RASi = renin-angiotensin system inhibitor; SGLT-2i = sodium-glucose cotransporter 2 inhibitor; T2D = type 2 diabetes; US = United States.

Supplementary Table S4. Country-specific CKD therapy costs* for Germany.

Intervention	Pharmacological Therapy Costed	Details	Unit cost – Euro	Annual cost – Euro	Reference	Reference description
SGLT-2i	Dapagliflozin	10 mg OD	76.46	997.39	NAVLIN 2023[S16]	NAVLIN
RASi	Irbesartan	75-300 mg OD[a]	14.78	192.76	Federal Institute for Drugs and Medical Devices 2024[S17,18]	HTA report
MRA	Finerenone	20 mg OD	355.76	1325.93	Online Pharmacy 2024[S19,20]	Online store for doctors, patients, health insurance prescriptions etc.
GLP-1ra	Semaglutide	0.5/1.0 mg injection QW	216.7	3756.13	Online Pharmacy 2024[S21,22]	Online store for doctors, patients, health insurance prescriptions etc.
Statin	Simvastatin plus ezetimibe	20 mg + 10 mg OD	57.3	209.29	Federal Institute for Drugs and Medical Devices 2024[S17,23]	HTA report

*Lifestyle intervention was assumed to have a cost of €0.
[a]Average between 75 mg, 150 mg, and 300 mg unit costs given range of dosing used in trial that informed eGFR treatment benefit.[S6]
Abbreviations: ACEi = angiotensin-converting enzyme inhibitor; ARB = angiotensin 2 receptor blockers; CKD = chronic kidney disease; eGFR = estimated glomerular filtration rate; HTA = health technology assessment; GLP-1ra = glucagon-like peptide 1 receptor agonist; MRA = mineralocorticoid receptor antagonist; OD = once daily; QW = once weekly; RASi = renin-angiotensin system inhibitor; SGLT-2i = sodium-glucose cotransporter 2 inhibitor.

Supplementary Table S5. Country-specific CKD therapy costs* for Netherlands.

Intervention	Pharmacological Therapy Costed	Details	Unit cost – Euro	Annual cost – Euro	Reference	Reference description
SGLT-2i	Dapagliflozin	10 mg OD	1.47	536.92	Healthcare Institute of the Netherlands 2024[S24]	Formulary
RASi	Irbesartan	75-300 mg OD[a]	0.053	19.48	Healthcare Institute of the Netherlands 2024[S25-27]	Formulary
MRA	Finerenone	25 mg OD	2.15	785.29	Healthcare Institute of the Netherlands 2024[S28]	Formulary
GLP-1ra	Semaglutide	14 mg OD or 0.5/1.0 mg injection QW[b]	4.01 (14 mg); 94.78 (0.5/1.0 mg)	3235.70	Healthcare Institute of the Netherlands 2024[S21,22]	Formulary
Statin	Simvastatin plus ezetimibe	20 mg + 10 mg OD	0.48	175.32	Healthcare Institute of the Netherlands 2024[S29]	Formulary

*Lifestyle intervention was assumed to have a cost of €0.
[a]Average between 75 mg, 150 mg, and 300 mg unit costs given range of dosing used in trial that informed eGFR treatment benefit.[S6]
[b]Weighted average performed with the patient counts from studies that informed eGFR treatment effect (14 mg OD, N = 1591; 0.5 mg QW, N = 826 patients; 1 mg QW, N = 822 patients).[S8]
Abbreviations: ACEi = angiotensin-converting enzyme inhibitor; ARB = angiotensin 2 receptor blockers; CKD = chronic kidney disease; eGFR = estimated glomerular filtration rate; GLP-1ra = glucagon-like peptide 1 receptor agonist; MRA = mineralocorticoid receptor antagonist; OD = once daily; QW = once weekly; RASi = renin-angiotensin system inhibitor; SGLT-2i = sodium-glucose cotransporter 2 inhibitor.

Supplementary Table S6. Country-specific CKD therapy costs* for Spain.

Intervention	Pharmacological Therapy Costed	Details	Unit cost – Euro	Annual cost – Euro	Reference	Reference description
SGLT-2i	Dapagliflozin	10 mg OD	46.02	600.31	Spanish Ministry of Health 2024[S30]	Formulary
RASi	Irbesartan	75-300 mg OD[a]	9.03	117.84	Spanish Ministry of Health 2024[S30]	Formulary
MRA	Spironolactone (proxy for Finerenone)	25 mg OD	2.5	18.26	Spanish Ministry of Health 2024[S30]	Formulary
GLP-1ra	Semaglutide	14 mg OD or 0.5/1.0 mg injection QW[b]	132.77 (14 mg); 128.15 (0.5/1.0 mg)	4196.19	Spanish Ministry of Health 2024[S30]	Formulary
Statin	Simvastatin plus ezetimibe	20 mg + 10 mg OD	32.72	426.82	Spanish Ministry of Health 2024[S30]	Formulary

[*]Lifestyle intervention was assumed to have a cost of €0.
[a]Average between 75 mg, 150 mg, and 300 mg unit costs given range of dosing used in trial that informed eGFR treatment benefit. [S6]
[b]Weighted average performed with the patient counts from studies that informed eGFR treatment effect (14 mg OD, N = 1591; 0.5 mg QW, N = 826 patients; 1 mg QW, N = 822 patients).[S8]
Abbreviations: ACEi = angiotensin-converting enzyme inhibitor; ARB = angiotensin 2 receptor blockers; CKD = chronic kidney disease; eGFR = estimated glomerular filtration rate; GLP-1ra = glucagon-like peptide 1 receptor agonist; MRA = mineralocorticoid receptor antagonist; OD = once daily; QW = once weekly; RASi = renin-angiotensin system inhibitor; SGLT-2i = sodium-glucose cotransporter 2 inhibitor.

Supplementary Table S7. Country-specific CKD therapy costs* for UK.

Intervention	Pharmacological Therapy Costed	Details	Unit cost – GBP	Annual cost – GBP	Reference	Reference description
SGLT-2i	Dapagliflozin	10 mg OD	36.59	477.30	NICE 2023[S31]	HTA report
RASi	Irbesartan	75-300 mg OD[a]	10.61	138.40	BNF 2020[S32]	Formulary
MRA	Finerenone	20 mg OD	36.68	478.48	NICE 2023[S33]	HTA report
GLP-1ra	Semaglutide	14 mg OD or 0.5/1.0 mg injection QW[b]	78.48 (14 mg); 73.25 (0.5/1.0 mg)	2413.68	NHS 2020[S8,34]	HTA report
Statin	Simvastatin plus ezetimibe	20 mg + 10 mg OD	33.42	435.95	BNF 2020[S32,35]	Formulary

[*]Lifestyle intervention was assumed to have a cost of £0.
[a]Average between 75 mg, 150 mg, and 300 mg unit costs given range of dosing used in trial that informed eGFR treatment benefit.[S6]
[b]Weighted average performed with the patient counts from studies that informed eGFR treatment effect (14 mg OD, N = 1591; 0.5 mg QW, N = 826 patients; 1 mg QW, N = 822 patients).[S8]
Abbreviations: ACEi = angiotensin-converting enzyme inhibitor; ARB = angiotensin 2 receptor blockers; BNF = British National Formulary; CKD = chronic kidney disease; eGFR = estimated glomerular filtration rate; GBP = Great Britain Pounds; GLP-1ra = glucagon-like peptide 1 receptor agonist; HTA = health technology assessment; MRA = mineralocorticoid receptor antagonist; NHS = National Health Services; NICE = The National Institute for Health and Care Excellence; OD = once daily; QW = once weekly; RASi = renin-angiotensin system inhibitor; SGLT-2i = sodium-glucose cotransporter 2 inhibitor; UK = United Kingdoms.

Supplementary Table S8. Country-specific CKD therapy costs* for US.

Intervention	Pharmacological Therapy Costed	Details	Unit cost- USD	Annual cost-USD	Reference	Reference description
SGLT-2i	Dapagliflozin	10 mg OD	409.84	4918.08	Literature[S36]	Drug review
RASi	Irbesartan	300 mg OD	-	1200.93	Literature[S37]	Screening/treating nephropathy in T2D and hypertension
MRA	Finerenone	20 mg OD	18.97	6928.79	Literature[S38]	Bayer ISPOR poster for Finerenone (KERENDIA)
GLP-1ra	Semaglutide	14 mg OD or 0.5/1.0 mg injection QW[a]	847.04 (14 mg); 892.06 (0.5/1.0 mg)	10984.20	Literature[S39,40]	CEA of semaglutide in T2D
Statin	Simvastatin plus ezetimibe	20 mg + 10 mg OD	2.23	814.51	Literature[S41]	CEA of statins in CKD

*Lifestyle intervention was assumed to have a cost of $0.

[a]Weighted average performed with the patient counts from studies that informed eGFR treatment effect (14 mg OD, N = 1591; 0.5 mg QW, N = 826 patients; 1 mg QW, N = 822 patients).[S8]

Abbreviations: ACEi = angiotensin-converting enzyme inhibitor; ARB = angiotensin 2 receptor blockers; CEA = cost-effectiveness analysis; CKD = chronic kidney disease; eGFR = estimated glomerular filtration rate; GLP-1ra = glucagon-like peptide 1 receptor agonist; ISPOR = The Professional Society for Health Economics and Outcomes Research; MRA = mineralocorticoid receptor antagonist; OD = once daily; QW = once weekly; RASi = renin-angiotensin system inhibitor; SGLT-2i = sodium-glucose cotransporter 2 inhibitor; T2D = type 2 diabetes; USD = United States Dollar; US = United States.

Supplementary Table S9. Impact of individual GDMT therapies on the annual rate of eGFR decline.

Intervention	Relative reduction in rate of eGFR decline (intervention vs control)	Relative rate of eGFR decline[a]	Reference	Additional Notes
RASi (ACEi and ARB)	-33.6%	0.66	Evans, 2011[S6]	Irbesartan (ARB) as proxy for class
MRAs	-32.3%	0.68	Ruilope, 2023[S42]	Finerenone as proxy for drug class
SGLT-2i	-53.5%	0.47	Heerspink, 2020[S43]	Dapagliflozin as a proxy for drug class.
GLP-1ra	-37.8%	0.62	Tuttle, 2023[S8]	Semaglutide as a proxy for drug class
Lifestyle (optimal exercise)	-29.8%	0.70	Robinson-Cohen, 2014[S44]	Based on physical activity (>150 min per week
Statins	-9.3%	0.91	Haynes, 2015[S35]	Simvastatin plus ezetimibe as proxy for statins.

[a]Calculated as one minus the relative reduction in rate of eGFR decline.

Note: All treatment effects are assumed to be multiplicative (i.e., if a patient is assigned to multiple different treatments, the treatment factors will be multiplied together for an overall treatment factor).

Abbreviations: ACEi = angiotensin-converting enzyme inhibitor; ARB = angiotensin 2 receptor blockers; eGFR = estimated glomerular filtration rate; GDMT = guideline-directed medical therapies; GLP-1ra = glucagon-like peptide 1 receptor agonist; MRA = mineralocorticoid receptor antagonist; RASi = renin-angiotensin system inhibitor; SGLT-2i = sodium-glucose cotransporter 2 inhibitor.

Supplementary Table S10. Impact of individual GDMT therapies on the annual rate of clinical events.

Intervention	Hazard ratio	Note	Reference
**Impact of intervention on MI occurrence**			
RASi (ACEi and ARB)	0.725	Average between ACE and ARB treatment effects.<br><br>ACE = RR: 0.73 (95%CI 0.42, 1.28) Based on data from 1926 patients in 8 studies. Follow up Mean 37 months.<br><br>ARB = RR: 0.72 (95% CI 0.36, 1.44) Based on data from 1498 patients in 5 studies. Follow up Mean 31 months.	KDIGO 2021 BP[12]
MRAs	0.91	Non-fatal only; CKD + T2DM: RR: 0.91 (0.74-1.12); Finenerone as a proxy for MRAs	Agarwal 2022[S45]
SGLT-2	0.76	CKD +/- Diabetes; RR 0.76 (0.62-0.92)	Staplin 2021[S46]
GLP-1	0.85	CKD + Diabetes; (OR 0.85; 95% CI, 0.63–1.15; p = 0.30)	Kelly 2022[S47]
Lifestyle	1.00		
Statins	0.55	CKD, RR 0.55, 95% CI 0.42 to 0.73; I^2 = 0%;	Tunnicliffe 2023[S48]
**Impact of intervention on stroke occurrence**			
RASi (ACEi and ARB)	0.75	ACE =RR: 0.29 (95%CI 0.10, 0.83) vs. PBO or SoC. Based on data from 1480 patients in 4 studies. Follow up mean 48 months.	KDIGO 2021 BP[12]
MRAs	0.99	Non-fatal only; CKD + T2DM; RR: 0.99 (0.82-1.21); Finanerone as a proxy for MRAs	Agarwal 2022[S45]
SGLT-2	0.94	CKD +/- Diabetes; 0.94 (0.85-1.04)	Staplin 2021[S46]
GLP-1	0.78	CKD + Diabetes; (OR 0.78; 95% CI, 0.33–1.86; p = 0.58)	Kelly 2022[S47]
Lifestyle	1.00		
Statins	0.64	CKD; RR 0.64, 95% CI 0.37 to 1.08	Tunnicliffe 2023[S48]

Intervention	Hazard ratio	Note	Reference
**Impact of intervention on HHF occurrence**			
RASi (ACEi and ARB)	0.68	CKD + T2DM; Average between RENAAL (RR = 0.68) and IDNT (RR = 0.72)	Brenner 2001[S49] Berl 2003[S50]
MRAs	0.78	CKD + T2DM; 0.78 (0.66-0.92)	Agarwal 2022[S45]
SGLT-2	0.63	CKD +/- Diabetes; 0.63 (0.54-0.73)	Staplin 2021[S46]
GLP-1	1.00		
Lifestyle	1.00		
Statins	1.00	MA noted too few events in included studies to evaluate effect on hospitalization due to heart failure	Tunnicliffe 2023[S48]
**Impact of intervention on AKI occurrence**			
RASi (ACEi and ARB)	1.00	There were too few who experienced AKI, to determine whether ACEi/ARB made a difference.	KDIGO 2021 BP[12]
MRAs	1.00	Relative risk: 0.94 (95% CI 0.78 - 1.12); Assumed null effect	KDIGO 2022 Diabetes[14]
SGLT-2	0.82	CKD + Diabetes; RR 0.82 (95% CI 0.72–0.93; P = 0.003)	Mavrakanas 2023[S51]
GLP-1	1.00	0.98 (95%CI: 0.83-1.2); Assumed null effect	Yang 2022[S52]
Lifestyle	1.00		
Statins	1.00		

Note: All treatment effects are assumed to be multiplicative (i.e., if a patient is assigned to multiple different treatments, the treatment factors are multiplied together for an overall treatment factor).

Abbreviations: ACEi = angiotensin-converting enzyme inhibitor; AKI = acute kidney injury; ARB = angiotensin 2 receptor blockers; CI = confidence interval; CKD = chronic kidney disease; eGFR = estimated glomerular filtration rate; GDMT = guideline-directed medical therapies; GLP-1 = glucagon-like peptide 1; HHF = hospitalization due to heart failure; IDNT = Irbesartan Diabetic Nephropathy Trial; I^2 = I-square; MA = meta-analysis; MI = myocardial infarction; MRAs = mineralocorticoid receptor antagonist; OR = odds ratio; PBO = placebo; RASi = renin-angiotensin system inhibitor; RENAAL = Reduction of Endpoints in NIDDM with the Angiotensin II Antagonist Losartan; RR = risk reduction; SGLT-2i = sodium-glucose cotransporter 2 inhibitor; SoC = standard of care; T2DM = type 2 diabetes mellitus.

Supplementary Table S11. CKD burden over 10 and 25 years following a 25% increase of the diagnosed population compared to current practice.

Outcome	Time Horizon	Scenario	EUR	Germany	Netherlands	Spain	UK	Australia	Brazil	China	US
**Clinical Burden - Cumulative CKD and KRT prevalence**											
CKD stage 1-2	25 Years	Current practice	396.6 M	151.4 M	32.4 M	96.1 M	116.6 M	54.2 M	394.4 M	3.5 B	838.8 M
		25% increased diagnosis	396.6 M	151.4 M	32.4 M	96.1 M	116.6 M	54.2 M	394.4 M	3.5 B	838.8 M
		% Change	**0.0%**	**0.0%**	**0.0%**	**0.0%**	**0.0%**	**0.0%**	**0.0%**	**0.0%**	**0.0%**
	10 Years	Current practice	178.6 M	70.6 M	14.2 M	42.5 M	51.2 M	21.3 M	159.6 M	1.4 B	360.9 M
		25% increased diagnosis	178.6 M	70.6 M	14.2 M	42.5 M	51.2 M	21.3 M	159.6 M	1.4 B	360.9 M
		% Change	**0.0%**	**0.0%**	**0.0%**	**0.0%**	**0.0%**	**0.0%**	**0.0%**	**0.0%**	**0.0%**
CKD stage 3-5 (incl. KRT)	25 Years	Current practice	391.5 M	151.7 M	35.1 M	93.4 M	111.3 M	31.2 M	326.1 M	1.8 B	649.0 M
		25% increased diagnosis	391.5 M	151.7 M	35.1 M	93.4 M	111.3 M	31.2 M	326.1 M	1.8 B	649.0 M
		% Change	**0.0%**	**0.0%**	**0.0%**	**0.0%**	**0.0%**	**0.0%**	**0.0%**	**0.0%**	**0.0%**
	10 Years	Current practice	145.3 M	57.0 M	12.7 M	31.8 M	43.9 M	11.6 M	136.4 M	695.4 M	265.9 M
		25% increased diagnosis	145.3 M	57.0 M	12.7 M	31.8 M	43.9 M	11.6 M	136.4 M	695.4 M	265.9 M
		% Change	**0.0%**	**0.0%**	**0.0%**	**0.0%**	**0.0%**	**0.0%**	**0.0%**	**0.0%**	**0.0%**
Dialysis	25 Years	Current practice	8.5 M	4.9 M	0.2 M	1.8 M	1.6 M	0.7 M	10.1 M	59.5 M	26.9 M
		25% increased diagnosis	8.5 M	4.9 M	0.2 M	1.8 M	1.6 M	0.7 M	10.1 M	59.5 M	26.9 M
		% Change	**0.0%**	**0.0%**	**0.0%**	**0.0%**	**0.0%**	**0.0%**	**0.0%**	**0.0%**	**0.0%**
	10 Years	Current practice	2.7 M	1.5 M	0.1 M	0.5 M	0.5 M	0.2 M	2.8 M	14.3 M	9.8 M
		25% increased diagnosis	2.7 M	1.5 M	0.1 M	0.5 M	0.5 M	0.2 M	2.8 M	14.3 M	9.8 M
		% Change	**0.0%**	**0.0%**	**0.0%**	**0.0%**	**0.0%**	**0.0%**	**0.0%**	**0.0%**	**0.0%**
Transplant	25 Years	Current practice	5.8 M	2.0 M	0.5 M	1.5 M	1.8 M	0.5 M	3.3 M	8.6 M	12.1 M
		25% increased diagnosis	5.8 M	2.0 M	0.5 M	1.5 M	1.8 M	0.5 M	3.3 M	8.6 M	12.1 M
		% Change	**0.0%**	**0.0%**	**0.0%**	**0.0%**	**0.0%**	**0.0%**	**0.0%**	**0.0%**	**0.0%**
	10 Years	Current practice	1.9 M	0.6 M	0.2 M	0.5 M	0.6 M	0.2 M	1.0 M	2.8 M	4.0 M
		25% increased diagnosis	1.9 M	0.6 M	0.2 M	0.5 M	0.6 M	0.2 M	1.0 M	2.8 M	4.0 M
		% Change	**0.0%**	**0.0%**	**0.0%**	**0.0%**	**0.0%**	**0.0%**	**0.0%**	**0.0%**	**0.0%**

Outcome	Time Horizon	Scenario	EUR	Germany	Netherlands	Spain	UK	Australia	Brazil	China	US
**Clinical Burden – Cumulative number of clinical events**											
CV events (MI, stroke, HHF)	25 Years	Current practice	12.2 M	5.1 M	1.1 M	2.3 M	3.7 M	2.1 M	11.6 M	59.6 M	41.2 M
		25% increased diagnosis	12.2 M	5.1 M	1.1 M	2.3 M	3.7 M	2.1 M	11.6 M	59.6 M	41.2 M
		% Change	**0.0%**	**0.0%**	**0.0%**	**0.0%**	**0.0%**	**0.0%**	**0.0%**	**0.0%**	**0.0%**
	10 Years	Current practice	4.1 M	1.8 M	0.3 M	0.7 M	1.3 M	0.5 M	3.3 M	17.5 M	10.9 M
		25% increased diagnosis	4.1 M	1.8 M	0.3 M	0.7 M	1.3 M	0.5 M	3.3 M	17.5 M	10.9 M
		% Change	**0.0%**	**0.0%**	**0.0%**	**0.0%**	**0.0%**	**0.0%**	**0.0%**	**0.0%**	**0.0%**
Death Events	25 Years	Current practice	27.6 M	12.2 M	2.0 M	5.2 M	8.2 M	1.8 M	18.8 M	112.9 M	38.7 M
		25% increased diagnosis	27.6 M	12.2 M	2.0 M	5.2 M	8.2 M	1.8 M	18.8 M	112.9 M	38.7 M
		% Change	**0.0%**	**0.0%**	**0.0%**	**0.0%**	**0.0%**	**0.0%**	**0.0%**	**0.0%**	**0.0%**
	10 Years	Current practice	10.2 M	5.0 M	0.6 M	1.6 M	3.0 M	0.5 M	6.0 M	34.3 M	12.6 M
		25% increased diagnosis	10.2 M	5.0 M	0.6 M	1.6 M	3.0 M	0.5 M	6.0 M	34.3 M	12.6 M
		% Change	**0.0%**	**0.0%**	**0.0%**	**0.0%**	**0.0%**	**0.0%**	**0.0%**	**0.0%**	**0.0%**
**Cumulative Economic Burden[a]**											
Non-KRT CKD Costs[b]	25 Years	Current practice	€989.2 B	€620.6 B	€65.2 B	€190.7 B	£96.2 B	$154.1 B	R$1.1 T	¥73.0 T	$7.8 T
		25% increased diagnosis	€1.0 T	€631.2 B	€66.2 B	€194.6 B	£98.0 B	$157.3 B	R$1.1 T	¥74.3 T	$8.0 T
		% Change	**1.8%**	**1.7%**	**1.6%**	**2.1%**	**1.9%**	**2.1%**	**2.0%**	**1.8%**	**1.9%**
	10 Years	Current practice	€376.0 B	€240.1 B	€21.9 B	€69.1 B	£38.3 B	$57.2 B	R$468.4 B	¥27.7 T	$3.2 T
		25% increased diagnosis	€383.7 B	€244.7 B	€22.3 B	€70.8 B	£39.2 B	$58.6 B	R$479.0 B	¥28.3 T	$3.3 T
		% Change	**2.0%**	**1.9%**	**2.1%**	**2.5%**	**2.1%**	**2.4%**	**2.3%**	**2.0%**	**2.2%**
CKD Treatment Costs	25 Years	Incremental Treatment Costs	€0	€0	€0	€0	£0	$0	R$0	¥0	$0
	10 Years	Incremental Treatment Costs	€0	€0	€0	€0	£0	$0	R$0	¥0	$0
KRT costs[b]	25 Years	Current practice	€451.7 B	€262.5 B	€21.0 B	€106.2 B	£52.9 B	$78.0 B	R$469.2 B	¥6.5 T	$3.4 T
		25% increased diagnosis	€451.7 B	€262.5 B	€21.0 B	€106.2 B	£52.9 B	$78.0 B	R$469.2 B	¥6.5 T	$3.4 T
		% Change	**0.0%**	**0.0%**	**0.0%**	**0.0%**	**0.0%**	**0.0%**	**0.0%**	**0.0%**	**0.0%**
	10 Years	Current practice	€145.7 B	€83.7 B	€8.6 B	€32.2 B	£18.2 B	$27.3 B	R$132.9 B	¥1.6 T	$1.2 T
		25% increased diagnosis	€145.7 B	€83.7 B	€8.6 B	€32.2 B	£18.2 B	$27.3 B	R$132.9 B	¥1.6 T	$1.2 T
		% Change	**0.0%**	**0.0%**	**0.0%**	**0.0%**	**0.0%**	**0.0%**	**0.0%**	**0.0%**	**0.0%**

Outcome	Time Horizon	Scenario	EUR	Germany	Netherlands	Spain	UK	Australia	Brazil	China	US
Total Costs of non-KRT CKD & KRT[b]	25 Years	Current practice	€1.4 T	€883.1 B	€86.1 B	€296.9 B	£149.1 B	$232.0 B	R$1.6 T	¥79.4 T	$11.2 T
		25% increased diagnosis	€1.5 T	€893.7 B	€87.2 B	€300.8 B	£150.8 B	$235.2 B	R$1.6 T	¥80.7 T	$11.3 T
		% Change	**1.2%**	**1.2%**	**1.2%**	**1.3%**	**1.2%**	**1.4%**	**1.4%**	**1.6%**	**1.3%**
	10 Years	Current practice	€521.8 B	€323.8 B	€30.5 B	€101.2 B	£56.5 B	$84.6 B	R$601.3 B	¥29.3 T	$4.4 T
		25% increased diagnosis	€529.4 B	€328.4 B	€30.9 B	€102.9 B	£57.3 B	$85.9 B	R$611.9 B	¥29.9 T	$4.5 T
		% Change	**1.5%**	**1.4%**	**1.5%**	**1.7%**	**1.5%**	**1.6%**	**1.8%**	**1.9%**	**1.6%**
Total Costs of non-KRT CKD & KRT Including Treatment Costs[c]	25 Years	Current practice	€1.4 T	€883.1 B	€86.1 B	€296.9 B	£149.1 B	$232.0 B	R$1.6 T	¥79.4 T	$11.2 T
		25% increased diagnosis	€1.5 T	€893.7 B	€87.2 B	€300.8 B	£150.8 B	$235.2 B	R$1.6 T	¥80.7 T	$11.3 T
		% Change	**1.2%**	**1.2%**	**1.2%**	**1.3%**	**1.2%**	**1.4%**	**1.4%**	**1.6%**	**1.3%**
	10 Years	Current practice	€521.8 B	€323.8 B	€30.5 B	€101.2 B	£56.5 B	$84.6 B	R$601.3 B	¥29.3 T	$4.4 T
		25% increased diagnosis	€529.4 B	€328.4 B	€30.9 B	€102.9 B	£57.3 B	$85.9 B	R$611.9 B	¥29.9 T	$4.5 T
		% Change	**1.5%**	**1.4%**	**1.5%**	**1.7%**	**1.5%**	**1.6%**	**1.8%**	**1.9%**	**1.6%**
**Cumulative Environmental Burden**											
All CKD (incl. KRT) Freshwater Consumption (m^3)	25 Years	Current practice	2.5 B	969.5 M	210.5 M	732.9 M	576.2 M	283.4 M	10.6 B	18.9 B	87.7 B
		25% increased diagnosis	2.5 B	978.1 M	213.1 M	740.8 M	582.7 M	286.2 M	10.7 B	19.1 B	88.6 B
		% Change	**1.0%**	**0.9%**	**1.2%**	**1.1%**	**1.1%**	**1.0%**	**1.1%**	**0.8%**	**1.0%**
	10 Years	Current practice	960.9 M	375.7 M	81.9 M	270.2 M	233.0 M	107.0 M	4.2 B	7.0 B	36.4 B
		25% increased diagnosis	971.8 M	379.5 M	83.0 M	273.3 M	235.9 M	108.1 M	4.2 B	7.0 B	36.8 B
		% Change	**1.1%**	**1.0%**	**1.3%**	**1.2%**	**1.3%**	**1.1%**	**1.3%**	**0.9%**	**1.2%**
All CKD (incl. KRT) Fossil Fuel Depletion (kg oil eq)	25 Years	Current practice	192.5 B	71.1 B	14.8 B	53.3 B	53.4 B	76.4 B	176.7 B	1.7 T	1.3 T
		25% increased diagnosis	194.6 B	71.8 B	14.9 B	53.9 B	54.0 B	77.2 B	178.6 B	1.7 T	1.3 T
		% Change	**1.1%**	**1.0%**	**1.3%**	**1.1%**	**1.2%**	**1.1%**	**1.1%**	**0.9%**	**1.1%**
	10 Years	Current practice	76.0 B	28.5 B	5.7 B	19.9 B	21.8 B	29.0 B	70.0 B	639.4 B	530.9 B
		25% increased diagnosis	76.9 B	28.9 B	5.8 B	20.2 B	22.1 B	29.3 B	70.9 B	645.5 B	537.2 B
		% Change	**1.2%**	**1.1%**	**1.4%**	**1.2%**	**1.3%**	**1.2%**	**1.3%**	**1.0%**	**1.2%**

Outcome	Time Horizon	Scenario	EUR	Germany	Netherlands	Spain	UK	Australia	Brazil	China	US
All CKD (incl. KRT) Carbon Footprint (kg CO_2 eq)	25 Years	Current practice	467.2 B	186.3 B	37.4 B	122.7 B	120.7 B	25.9 B	496.6 B	4.7 T	3.4 T
		25% increased diagnosis – 25 years	472.4 B	188.2 B	37.9 B	124.1 B	122.1 B	26.2 B	501.8 B	4.7 T	3.5 T
		% Change	**1.1%**	**1.0%**	**1.3%**	**1.1%**	**1.2%**	**1.1%**	**1.1%**	**0.9%**	**1.1%**
	10 Years	Current practice	184.5 B	74.7 B	14.5 B	46.0 B	49.3 B	9.8 B	196.3 B	1.7 T	1.4 T
		25% increased diagnosis	186.7 B	75.5 B	14.7 B	46.5 B	50.0 B	9.9 B	198.7 B	1.8 T	1.4 T
		% Change	**1.2%**	**1.1%**	**1.4%**	**1.2%**	**1.3%**	**1.2%**	**1.3%**	**1.0%**	**1.3%**
KRT only Freshwater Consumption (m^3)	25 Years	Current practice	486.4 M	275.3 M	17.8 M	112.4 M	80.9 M	50.2 M	1.7 B	2.7 B	6.9 B
		25% increased diagnosis	486.4 M	275.3 M	17.8 M	112.4 M	80.9 M	50.2 M	1.7 B	2.7 B	6.9 B
		% Change	**0.0%**	**0.0%**	**0.0%**	**0.0%**	**0.0%**	**0.0%**	**0.0%**	**0.0%**	**0.0%**
	10 Years	Current practice	156.6 M	87.2 M	7.5 M	34.0 M	28.0 M	17.6 M	461.4 M	665.0 M	2.5 B
		25% increased diagnosis	156.6 M	87.2 M	7.5 M	34.0 M	28.0 M	17.6 M	461.4 M	665.0 M	2.5 B
		% Change	**0.0%**	**0.0%**	**0.0%**	**0.0%**	**0.0%**	**0.0%**	**0.0%**	**0.0%**	**0.0%**
KRT only Fossil Fuel Depletion (kg oil eq)	25 Years	Current practice	21.9 B	12.0 B	649.4 M	5.0 B	4.2 B	8.5 B	23.3 B	171.3 B	95.7 B
		25% increased diagnosis	21.9 B	12.0 B	649.4 M	5.0 B	4.2 B	8.5 B	23.3 B	171.3 B	95.7 B
		% Change	**0.0%**	**0.0%**	**0.0%**	**0.0%**	**0.0%**	**0.0%**	**0.0%**	**0.0%**	**0.0%**
	10 Years	Current practice	7.1 B	3.8 B	261.1 M	1.5 B	1.5 B	3.0 B	6.5 B	41.8 B	34.6 B
		25% increased diagnosis	7.1 B	3.8 B	261.1 M	1.5 B	1.5 B	3.0 B	6.5 B	41.8 B	34.6 B
		% Change	**0.0%**	**0.0%**	**0.0%**	**0.0%**	**0.0%**	**0.0%**	**0.0%**	**0.0%**	**0.0%**
KRT only Carbon Footprint (kg CO_2 eq)	25 Years	Current practice	56.3 B	33.0 B	1.8 B	12.0 B	9.5 B	2.8 B	68.5 B	485.0 B	231.2 B
		25% increased diagnosis	56.3 B	33.0 B	1.8 B	12.0 B	9.5 B	2.8 B	68.5 B	485.0 B	231.2 B
		% Change	**0.0%**	**0.0%**	**0.0%**	**0.0%**	**0.0%**	**0.0%**	**0.0%**	**0.0%**	**0.0%**
	10 Years	Current practice	18.1 B	10.5 B	714.9 M	3.7 B	3.3 B	1.0 B	19.0 B	118.2 B	83.8 B
		25% increased diagnosis	18.1 B	10.5 B	714.9 M	3.7 B	3.3 B	1.0 B	19.0 B	118.2 B	83.8 B
		% Change	**0.0%**	**0.0%**	**0.0%**	**0.0%**	**0.0%**	**0.0%**	**0.0%**	**0.0%**	**0.0%**

[a]Currency conversion for the UK from GBP (£) to euro (€) was performed prior to aggregation across European countries using the 2022 annual average from ECB.[26] Values used in conversions were as follows: £1.0 = €1.173.

[b]Does not include costs associated with CKD treatment or screening.

[c]Does not include costs associated with CKD screening.

Abbreviations: B = billion; CKD = chronic kidney disease; CV = cardiovascular; eq = equivalent; ECB = European Central Bank; EUR = Europe; GBP = pound sterling; HHF = hospitalization from heart failure; incl. = including; KRT = kidney replacement therapy; M = million; MI = myocardial infarction; T = trillion; UK = United Kingdoms; US = United States.

Supplementary Table S12. CKD burden over 10 and 25 years following an increase of GDMT adherence to 75% compared to current practice.

Outcome	Time Horizon	Scenario	EUR	Germany	Netherlands	Spain	UK	Australia	Brazil	China	US
**Clinical Burden - Cumulative CKD and KRT prevalence**											
CKD stage 1-2	25 Years	Current practice	396.6 M	151.4 M	32.4 M	96.1 M	116.6 M	54.2 M	394.4 M	3.5 B	838.8 M
		75% adherence to GDMT	400.6 M	152.9 M	32.8 M	97.3 M	117.6 M	54.8 M	397.9 M	3.5 B	848.4 M
		% Change	**1.0%**	**1.0%**	**1.2%**	**1.2%**	**0.9%**	**1.1%**	**0.9%**	**0.8%**	**1.1%**
	10 Years	Current practice	178.6 M	70.6 M	14.2 M	42.5 M	51.2 M	21.3 M	159.6 M	1.4 B	360.9 M
		75% adherence to GDMT	179.4 M	71.0 M	14.3 M	42.8 M	51.4 M	21.4 M	160.3 M	1.4 B	362.9 M
		% Change	**0.5%**	**0.5%**	**0.5%**	**0.5%**	**0.4%**	**0.5%**	**0.5%**	**0.4%**	**0.6%**
CKD stage 3-5 (incl. KRT)	25 Years	Current practice	391.5 M	151.7 M	35.1 M	93.4 M	111.3 M	31.2 M	326.1 M	1.8 B	649.0 M
		75% adherence to GDMT	399.9 M	154.9 M	36.0 M	95.8 M	113.3 M	31.5 M	338.4 M	1.9 B	668.5 M
		% Change	**2.1%**	**2.1%**	**2.5%**	**2.5%**	**1.7%**	**1.0%**	**3.8%**	**1.6%**	**3.0%**
	10 Years	Current practice	145.3 M	57.0 M	12.7 M	31.8 M	43.9 M	11.6 M	136.4 M	695.4 M	265.9 M
		75% adherence to GDMT	146.5 M	57.4 M	12.8 M	32.1 M	44.2 M	11.6 M	138.1 M	697.3 M	267.7 M
		% Change	**0.8%**	**0.8%**	**1.0%**	**0.9%**	**0.8%**	**-0.1%**	**1.2%**	**0.3%**	**0.7%**
Dialysis	25 Years	Current practice	8.5 M	4.9 M	0.2 M	1.8 M	1.6 M	0.7 M	10.1 M	59.5 M	26.9 M
		75% adherence to GDMT	5.4 M	3.0 M	0.2 M	1.1 M	1.1 M	0.4 M	6.0 M	35.2 M	18.4 M
		% Change	**-36.0%**	**-37.9%**	**-20.1%**	**-40.4%**	**-26.6%**	**-48.2%**	**-40.5%**	**-40.8%**	**-31.5%**
	10 Years	Current practice	2.7 M	1.5 M	0.1 M	0.5 M	0.5 M	0.2 M	2.8 M	14.3 M	9.8 M
		75% adherence to GDMT	2.5 M	1.4 M	0.1 M	0.5 M	0.5 M	0.2 M	2.4 M	11.9 M	9.0 M
		% Change	**-9.1%**	**-8.8%**	**-19.5%**	**-14.4%**	**-3.2%**	**-12.8%**	**-13.3%**	**-17.2%**	**-8.2%**
Transplant	25 Years	Current practice	5.8 M	2.0 M	0.5 M	1.5 M	1.8 M	0.5 M	3.3 M	8.6 M	12.1 M
		75% adherence to GDMT	5.8 M	2.0 M	0.5 M	1.5 M	1.8 M	0.5 M	3.3 M	8.6 M	12.1 M
		% Change	**0.0%**	**0.0%**	**-0.4%**	**0.7%**	**-0.5%**	**0.1%**	**0.8%**	**0.3%**	**-0.2%**
	10 Years	Current practice	1.9 M	0.6 M	0.2 M	0.5 M	0.6 M	0.2 M	1.0 M	2.8 M	4.0 M
		75% adherence to GDMT	1.9 M	0.6 M	0.2 M	0.5 M	0.6 M	0.2 M	1.0 M	2.8 M	4.0 M
		% Change	**-0.1%**	**0.0%**	**-0.1%**	**-0.1%**	**-0.3%**	**-0.1%**	**0.0%**	**0.0%**	**-0.2%**

Outcome	Time Horizon	Scenario	EUR	Germany	Netherlands	Spain	UK	Australia	Brazil	China	US
**Clinical Burden – Cumulative number of clinical events**											
CV events (MI, stroke, HHF)	25 Years	Current practice	12.2 M	5.1 M	1.1 M	2.3 M	3.7 M	2.1 M	11.6 M	59.6 M	41.2 M
		75% adherence to GDMT	9.5 M	4.0 M	0.8 M	1.8 M	2.8 M	1.6 M	8.9 M	51.1 M	32.1 M
		% Change	**-22.4%**	**-21.4%**	**-24.4%**	**-21.4%**	**-23.9%**	**-22.1%**	**-23.4%**	**-14.2%**	**-22.1%**
	10 Years	Current practice	4.1 M	1.8 M	0.3 M	0.7 M	1.3 M	0.5 M	3.3 M	17.5 M	10.9 M
		75% adherence to GDMT	3.3 M	1.5 M	0.3 M	0.6 M	1.0 M	0.4 M	2.6 M	15.4 M	8.7 M
		% Change	**-19.5%**	**-19.8%**	**-19.8%**	**-17.7%**	**-20.0%**	**-18.8%**	**-21.0%**	**-12.2%**	**-19.7%**
Death Events	25 Years	Current practice	27.6 M	12.2 M	2.0 M	5.2 M	8.2 M	1.8 M	18.8 M	112.9 M	38.7 M
		75% adherence to GDMT	26.9 M	11.9 M	1.9 M	4.9 M	8.1 M	1.7 M	17.8 M	108.5 M	36.7 M
		% Change	**-2.5%**	**-2.0%**	**-3.7%**	**-4.5%**	**-1.8%**	**-4.0%**	**-5.0%**	**-3.9%**	**-5.2%**
	10 Years	Current practice	10.2 M	5.0 M	0.6 M	1.6 M	3.0 M	0.5 M	6.0 M	34.3 M	12.6 M
		75% adherence to GDMT	9.9 M	4.9 M	0.6 M	1.5 M	2.9 M	0.5 M	5.6 M	33.3 M	11.9 M
		% Change	**-2.9%**	**-2.4%**	**-5.4%**	**-5.0%**	**-2.3%**	**-3.5%**	**-5.6%**	**-3.2%**	**-5.3%**
**Cumulative Economic Burden[a]**											
Non-KRT CKD Costs[b]	25 Years	Current practice	€989.2 B	€620.6 B	€65.2 B	€190.7 B	£96.2 B	$154.1 B	R$1.1 T	¥73.0 T	$7.8 T
		75% adherence to GDMT	€988.7 B	€629.4 B	€56.9 B	€190.6 B	£95.2 B	$147.8 B	R$1.1 T	¥73.1 T	$8.2 T
		% Change	**-0.1%**	**1.4%**	**-12.7%**	**0.0%**	**-1.0%**	**-4.1%**	**3.6%**	**0.2%**	**4.5%**
	10 Years	Current practice	€376.0 B	€240.1 B	€21.9 B	€69.1 B	£38.3 B	$57.2 B	R$468.4 B	¥27.7 T	$3.2 T
		75% adherence to GDMT	€369.0 B	€236.8 B	€19.8 B	€68.1 B	£37.8 B	$54.3 B	R$466.7 B	¥27.3 T	$3.2 T
		% Change	**-1.9%**	**-1.4%**	**-9.6%**	**-1.4%**	**-1.4%**	**-5.2%**	**-0.4%**	**-1.7%**	**0.5%**
CKD Treatment Costs	25 Years	Incremental Treatment Costs	€107.2 B	€54.3 B	€7.2 B	€19.9 B	£22.0 B	$9.6 B	R$342.9 B	¥4.3 T	$997.3 B
	10 Years	Incremental Treatment Costs	€38.8 B	€19.3 B	€2.7 B	€6.9 B	£8.5 B	$3.6 B	R$121.4 B	¥1.7 T	$405.9 B

Outcome	Time Horizon	Scenario	EUR	Germany	Netherlands	Spain	UK	Australia	Brazil	China	US
KRT costs[b]	25 Years	Current practice	€451.7 B	€262.5 B	€21.0 B	€106.2 B	£52.9 B	$78.0 B	R$469.2 B	¥6.5 T	$3.4 T
		75% adherence to GDMT	€310.1 B	€174.6 B	€18.0 B	€68.0 B	£42.3 B	$46.3 B	R$307.4 B	¥4.1 T	$2.5 T
		% Change	**-31.3%**	**-33.5%**	**-14.4%**	**-36.0%**	**-20.1%**	**-40.6%**	**-34.5%**	**-37.3%**	**-25.5%**
	10 Years	Current practice	€145.7 B	€83.7 B	€8.6 B	€32.2 B	£18.2 B	$27.3 B	R$132.9 B	¥1.6 T	$1.2 T
		75% adherence to GDMT	€133.5 B	€77.3 B	€7.3 B	€28.1 B	£17.7 B	$24.4 B	R$118.2 B	¥1.4 T	$1.1 T
		% Change	**-8.4%**	**-7.7%**	**-14.9%**	**-12.6%**	**-2.5%**	**-10.8%**	**-11.0%**	**-15.3%**	**-6.7%**
Total Costs of non-KRT CKD & KRT[b]	25 Years	Current practice	€1.4 T	€883.1 B	€86.1 B	€296.9 B	£149.1 B	$232.0 B	R$1.6 T	¥79.4 T	$11.2 T
		75% adherence to GDMT	€1.3 T	€804.0 B	€74.9 B	€258.6 B	£137.5 B	$194.2 B	R$1.5 T	¥77.2 T	$10.7 T
		% Change	**-9.9%**	**-9.0%**	**-13.1%**	**-12.9%**	**-7.8%**	**-16.3%**	**-7.8%**	**-2.8%**	**-4.5%**
	10 Years	Current practice	€521.8 B	€323.8 B	€30.5 B	€101.2 B	£56.5 B	$84.6 B	R$601.3 B	¥29.3 T	$4.4 T
		75% adherence to GDMT	€502.5 B	€314.1 B	€27.1 B	€96.2 B	£55.5 B	$78.7 B	R$584.9 B	¥28.6 T	$4.4 T
		% Change	**-3.7%**	**-3.0%**	**-11.1%**	**-5.0%**	**-1.8%**	**-7.0%**	**-2.7%**	**-2.4%**	**-1.5%**
Total Costs of non-KRT CKD & KRT Including Treatment Costs[c]	25 Years	Current practice	€1.4 T	€883.1 B	€86.1 B	€296.9 B	£149.1 B	$232.0 B	R$1.6 B	¥79.4 T	$11.2 T
		75% adherence to GDMT	€1.4 T	€858.3 B	€82.1 B	€278.5 B	£159.5 B	$203.8 B	R$1.8 B	¥81.5 T	$11.7 T
		% Change	**-2.4%**	**-2.8%**	**-4.7%**	**-6.2%**	**7.0%**	**-12.2%**	**14.0%**	**2.6%**	**4.4%**
	10 Years	Current practice	€521.8 B	€323.8 B	€30.5 B	€101.2 B	£56.5 B	$84.6 B	R$601.3 B	¥29.3 T	$4.4 T
		75% adherence to GDMT	€541.3 B	€333.4 B	€29.8 B	€103.1 B	£64.0 B	$82.3 B	R$706.3 B	¥30.3 T	$4.8 T
		% Change	**3.7%**	**3.0%**	**-2.2%**	**1.8%**	**13.2%**	**-2.7%**	**17.5%**	**3.3%**	**7.7%**
**Cumulative Environmental Burden**											
All CKD (incl. KRT) Freshwater Consumption (m^3)	25 Years	Current practice	2.5 B	969.5 M	210.5 M	732.9 M	576.2 M	283.4 M	10.6 B	18.9 B	87.7 B
		75% adherence to GDMT	2.4 B	881.0 M	209.5 M	700.0 M	561.2 M	262.8 M	10.2 B	18.0 B	88.4 B
		% Change	**-5.5%**	**-9.1%**	**-0.5%**	**-4.5%**	**-2.6%**	**-7.3%**	**-3.8%**	**-4.7%**	**0.8%**
	10 Years	Current practice	960.9 M	375.7 M	81.9 M	270.2 M	233.0 M	107.0 M	4.2 B	7.0 B	36.4 B
		75% adherence to GDMT	947.0 M	367.8 M	81.0 M	266.0 M	232.2 M	104.5 M	4.1 B	6.9 B	36.5 B
		% Change	**-1.4%**	**-2.1%**	**-1.2%**	**-1.6%**	**-0.3%**	**-2.4%**	**-1.3%**	**-1.6%**	**0.2%**

Outcome	Time Horizon	Scenario	EUR	Germany	Netherlands	Spain	UK	Australia	Brazil	China	US
All CKD (incl. KRT) Fossil Fuel Depletion (kg oil eq)	25 Years	Current practice	192.5 B	71.1 B	14.8 B	53.3 B	53.4 B	76.4 B	176.7 B	1.7 T	1.3 T
		75% adherence to GDMT	187.8 B	67.9 B	14.8 B	52.2 B	52.9 B	73.3 B	171.9 B	1.7 T	1.3 T
		% Change	**-2.4%**	**-4.4%**	**0.3%**	**-2.0%**	**-1.0%**	**-4.1%**	**-2.7%**	**-2.9%**	**1.0%**
	10 Years	Current practice	76.0 B	28.5 B	5.7 B	19.9 B	21.8 B	29.0 B	70.0 B	639.4 B	530.9 B
		75% adherence to GDMT	75.5 B	28.2 B	5.7 B	19.8 B	21.8 B	28.5 B	69.4 B	632.3 B	532.6 B
		% Change	**-0.7%**	**-1.1%**	**-0.3%**	**-0.9%**	**-0.2%**	**-1.7%**	**-0.9%**	**-1.1%**	**0.3%**
All CKD (incl. KRT) Carbon Footprint (kg CO_2 eq)	25 Years	Current practice	467.2 B	186.3 B	37.4 B	122.7 B	120.7 B	25.9 B	496.6 B	4.7 T	3.4 T
		75% adherence to GDMT	454.7 B	177.4 B	37.5 B	120.1 B	119.6 B	24.9 B	481.7 B	4.5 T	3.4 T
		% Change	**-2.7%**	**-4.8%**	**0.3%**	**-2.2%**	**-0.9%**	**-4.0%**	**-3.0%**	**-3.0%**	**-0.7%**
	10 Years	Current practice	184.5 B	74.7 B	14.5 B	46.0 B	49.3 B	9.8 B	196.3 B	1.7 T	1.4 T
		75% adherence to GDMT	183.2 B	73.9 B	14.5 B	45.6 B	49.3 B	9.7 B	194.3 B	1.7 T	1.4 T
		% Change	**-0.7%**	**-1.1%**	**-0.3%**	**-0.9%**	**-0.2%**	**-1.6%**	**-1.0%**	**-1.1%**	**-1.6%**
KRT only Freshwater Consumption (m^3)	25 Years	Current practice	486.4 M	275.3 M	17.8 M	112.4 M	80.9 M	50.2 M	1.7 B	2.7 B	6.9 B
		75% adherence to GDMT	322.9 M	175.1 M	14.9 M	71.2 M	61.7 M	27.8 M	1.0 B	1.6 B	4.9 B
		% Change	**-33.6%**	**-36.4%**	**-16.5%**	**-36.6%**	**-23.8%**	**-44.6%**	**-38.4%**	**-39.9%**	**-28.3%**
	10 Years	Current practice	156.6 M	87.2 M	7.5 M	34.0 M	28.0 M	17.6 M	461.4 M	665.0 M	2.5 B
		75% adherence to GDMT	142.9 M	79.9 M	6.2 M	29.6 M	27.2 M	15.5 M	403.5 M	553.7 M	2.3 B
		% Change	**-8.8%**	**-8.4%**	**-16.7%**	**-12.9%**	**-2.9%**	**-11.9%**	**-12.6%**	**-16.7%**	**-7.4%**
KRT only Fossil Fuel Depletion (kg oil eq)	25 Years	Current practice	21.9 B	12.0 B	649.4 M	5.0 B	4.2 B	8.5 B	23.3 B	171.3 B	95.7 B
		75% adherence to GDMT	14.9 B	7.7 B	562.3 M	3.3 B	3.3 B	4.9 B	14.5 B	103.6 B	68.8 B
		% Change	**-31.9%**	**-35.4%**	**-13.4%**	**-34.2%**	**-21.7%**	**-42.2%**	**-37.9%**	**-39.5%**	**-28.1%**
	10 Years	Current practice	7.1 B	3.8 B	261.1 M	1.5 B	1.5 B	3.0 B	6.5 B	41.8 B	34.6 B
		75% adherence to GDMT	6.5 B	3.5 B	224.3 M	1.4 B	1.4 B	2.7 B	5.7 B	34.9 B	32.1 B
		% Change	**-8.1%**	**-8.2%**	**-14.1%**	**-11.9%**	**-2.7%**	**-11.3%**	**-12.4%**	**-16.5%**	**-7.4%**

Outcome	Time Horizon	Scenario	EUR	Germany	Netherlands	Spain	UK	Australia	Brazil	China	US
KRT only Carbon Footprint (kg CO_2 eq)	25 Years	Current practice	56.3 B	33.0 B	1.8 B	12.0 B	9.5 B	2.8 B	68.5 B	485.0 B	231.2 B
		75% adherence to GDMT	38.1 B	21.3 B	1.5 B	7.9 B	7.5 B	1.6 B	42.4 B	293.2 B	166.1 B
		% Change	**-32.3%**	**-35.6%**	**-13.9%**	**-34.4%**	**-21.7%**	**-42.0%**	**-38.1%**	**-39.5%**	**-28.2%**
	10 Years	Current practice	18.1 B	10.5 B	714.9 M	3.7 B	3.3 B	991.2 M	19.0 B	118.2 B	83.8 B
		75% adherence to GDMT	16.6 B	9.6 B	611.3 M	3.2 B	3.2 B	879.5 M	16.7 B	98.6 B	77.6 B
		% Change	**-8.2%**	**-8.2%**	**-14.5%**	**-12.0%**	**-2.7%**	**-11.3%**	**-12.4%**	**-16.6%**	**-7.4%**

[a]Currency conversion for the UK from GBP (£) to euro (€) was performed prior to aggregation across European countries using the 2022 annual average from ECB.[26] Values used in conversions were as follows: £1.0 = €1.173.

[b]Does not include costs associated with CKD treatment or screening.

[c]Does not include costs associated with CKD screening.

Abbreviations: B = billion; CKD = chronic kidney disease; CV = cardiovascular; eq = equivalent; ECB = European Central Bank; EUR = Europe; GBP = pound sterling; GDMT = guideline-directed medical therapy; HHF = hospitalization from heart failure; incl. = including; KRT = kidney replacement therapy; M = million; MI = myocardial infarction; T = trillion; UK = United Kingdoms; US = United States.

Supplementary Table S13. CKD burden over 10 and 25 years following a 25% increase of diagnosed population and an increase to GDMT adherence to 75% compared to current practice.

Outcome	Time Horizon	Scenario	EUR	Germany	Netherlands	Spain	UK	Australia	Brazil	China	US
**Clinical Burden - Cumulative CKD and KRT prevalence**											
CKD stage 1-2	25 Years	Current practice	396.6 M	151.4 M	32.4 M	96.1 M	116.6 M	54.2 M	394.4 M	3.5 B	838.8 M
		25% increased diagnosis + 75% adherence to GDMT	401.6 M	153.2 M	32.9 M	97.6 M	117.9 M	55.0 M	398.6 M	3.5 B	850.7 M
		% Change	**1.3%**	**1.2%**	**1.4%**	**1.5%**	**1.1%**	**1.4%**	**1.1%**	**1.0%**	**1.4%**
	10 Years	Current practice	178.6 M	70.6 M	14.2 M	42.5 M	51.2 M	21.3 M	159.6 M	1.4 B	360.9 M
		25% increased diagnosis + 75% adherence to GDMT	179.7 M	71.1 M	14.3 M	42.8 M	51.5 M	21.4 M	160.5 M	1.4 B	363.4 M
		% Change	**0.6%**	**0.6%**	**0.7%**	**0.7%**	**0.5%**	**0.6%**	**0.6%**	**0.5%**	**0.7%**
CKD stage 3-5 (incl. KRT)	25 Years	Current practice	391.5 M	151.7 M	35.1 M	93.4 M	111.3 M	31.2 M	326.1 M	1.8 B	649.0 M
		25% increased diagnosis + 75% adherence to GDMT	401.0 M	155.3 M	36.1 M	96.1 M	113.5 M	31.5 M	340.3 M	1.9 B	671.2 M
		% Change	**2.4%**	**2.4%**	**2.8%**	**2.9%**	**2.0%**	**1.1%**	**4.3%**	**1.8%**	**3.4%**
	10 Years	Current practice	145.3 M	57.0 M	12.7 M	31.8 M	43.9 M	11.6 M	136.4 M	695.4 M	265.9 M
		25% increased diagnosis + 75% adherence to GDMT	146.7 M	57.5 M	12.8 M	32.1 M	44.3 M	11.6 M	138.4 M	697.7 M	267.9 M
		% Change	**1.0%**	**0.9%**	**1.2%**	**1.0%**	**0.9%**	**-0.1%**	**1.4%**	**0.3%**	**0.8%**
Dialysis	25 Years	Current practice	8.5 M	4.9 M	0.2 M	1.8 M	1.6 M	0.7 M	10.1 M	59.5 M	26.9 M
		25% increased diagnosis + 75% adherence to GDMT	4.9 M	2.8 M	0.1 M	1.0 M	1.1 M	0.3 M	5.5 M	31.3 M	17.3 M
		% Change	**-41.5%**	**-43.2%**	**-25.5%**	**-46.6%**	**-32.5%**	**-53.5%**	**-45.8%**	**-47.3%**	**-35.8%**
	10 Years	Current practice	2.7 M	1.5 M	0.1 M	0.5 M	0.5 M	0.2 M	2.8 M	14.3 M	9.8 M
		25% increased diagnosis + 75% adherence to GDMT	2.4 M	1.4 M	0.1 M	0.5 M	0.5 M	0.2 M	2.3 M	11.2 M	8.9 M
		% Change	**-10.9%**	**-10.4%**	**-22.9%**	**-15.9%**	**-5.5%**	**-14.5%**	**-16.7%**	**-21.7%**	**-9.9%**

Outcome	Time Horizon	Scenario	EUR	Germany	Netherlands	Spain	UK	Australia	Brazil	China	US
Transplant	25 Years	Current practice	5.8 M	2.0 M	0.5 M	1.5 M	1.8 M	0.5 M	3.3 M	8.6 M	12.1 M
		25% increased diagnosis + 75% adherence to GDMT	5.8 M	2.0 M	0.5 M	1.5 M	1.8 M	0.5 M	3.3 M	8.6 M	12.1 M
		% Change	**-0.2%**	**0.0%**	**-0.4%**	**0.3%**	**-0.7%**	**0.1%**	**0.5%**	**0.1%**	**-0.4%**
	10 Years	Current practice	1.9 M	0.6 M	0.2 M	0.5 M	0.6 M	0.2 M	1.0 M	2.8 M	4.0 M
		25% increased diagnosis + 75% adherence to GDMT	1.9 M	0.6 M	0.2 M	0.5 M	0.6 M	0.2 M	1.0 M	2.8 M	4.0 M
		% Change	**-0.2%**	**0.0%**	**-0.1%**	**-0.3%**	**-0.4%**	**-0.1%**	**0.0%**	**0.0%**	**-0.3%**
**Clinical Burden – Cumulative number of clinical events**											
CV events (MI, stroke, HHF)	25 Years	Current practice	12.2 M	5.1 M	1.1 M	2.3 M	3.7 M	2.1 M	11.6 M	59.6 M	41.2 M
		25% increased diagnosis + 75% adherence to GDMT	9.0 M	3.8 M	0.8 M	1.7 M	2.7 M	1.5 M	8.4 M	49.5 M	30.5 M
		% Change	**-26.6%**	**-25.5%**	**-29.0%**	**-25.4%**	**-28.3%**	**-26.6%**	**-27.8%**	**-17.0%**	**-26.0%**
	10 Years	Current practice	4.1 M	1.8 M	0.3 M	0.7 M	1.3 M	0.5 M	3.3 M	17.5 M	10.9 M
		25% increased diagnosis + 75% adherence to GDMT	3.2 M	1.4 M	0.2 M	0.6 M	1.0 M	0.4 M	2.5 M	15.0 M	8.3 M
		% Change	**-23.4%**	**-23.6%**	**-24.0%**	**-21.3%**	**-24.1%**	**-22.9%**	**-25.3%**	**-14.6%**	**-23.5%**
Death Events	25 Years	Current practice	27.6 M	12.2 M	2.0 M	5.2 M	8.2 M	1.8 M	18.8 M	112.9 M	38.7 M
		25% increased diagnosis + 75% adherence to GDMT	26.7 M	11.9 M	1.9 M	4.9 M	8.0 M	1.7 M	17.7 M	107.8 M	36.4 M
		% Change	**-3.0%**	**-2.4%**	**-4.4%**	**-5.3%**	**-2.1%**	**-4.7%**	**-5.8%**	**-4.5%**	**-6.0%**
	10 Years	Current practice	10.2 M	5.0 M	0.6 M	1.6 M	3.0 M	0.5 M	6.0 M	34.3 M	12.6 M
		25% increased diagnosis + 75% adherence to GDMT	9.8 M	4.8 M	0.6 M	1.5 M	2.9 M	0.5 M	5.6 M	33.0 M	11.8 M
		% Change	**-3.6%**	**-2.8%**	**-6.5%**	**-6.0%**	**-2.9%**	**-4.3%**	**-6.8%**	**-3.8%**	**-6.2%**

Outcome	Time Horizon	Scenario	EUR	Germany	Netherlands	Spain	UK	Australia	Brazil	China	US
**Cumulative Economic Burden[a]**											
Non-KRT CKD Costs[b]	25 Years	Current practice	€989.2 B	€620.6 B	€65.2 B	€190.7 B	£96.2 B	$154.1 B	R$1.1 T	¥73.0 T	$7.8 T
		25% increased diagnosis + 75% adherence to GDMT	€1.0 T	€641.0 B	€56.2 B	€193.9 B	£97.1 B	$148.7 B	R$1.2 T	¥74.7 T	$8.4 T
		% Change	**1.6%**	**3.3%**	**-13.8%**	**1.7%**	**0.9%**	**-3.5%**	**6.0%**	**2.3%**	**6.9%**
	10 Years	Current practice	€376.0 B	€240.1 B	€21.9 B	€69.1 B	£38.3 B	$57.2 B	R$468.4 B	¥27.7 T	$3.2 T
		25% increased diagnosis + 75% adherence to GDMT	€375.6 B	€241.1 B	€19.7 B	€69.5 B	£38.6 B	$54.9 B	R$477.5 B	¥27.8 T	$3.3 T
		% Change	**-0.1%**	**0.4%**	**-9.8%**	**0.7%**	**0.6%**	**-4.0%**	**1.9%**	**0.4%**	**2.8%**
CKD Treatment Costs	25 Years	Incremental Treatment Costs	€128.1 B	€64.8 B	€8.6 B	€23.7 B	£26.4 B	$11.6 B	R$407.3 B	¥5.2 T	$1.2 T
	10 Years	Incremental Treatment Costs	€47.2 B	€23.5 B	€3.3 B	€8.4 B	£10.3 B	$4.4 B	R$146.9 B	¥2.0 T	$491.2 B
KRT costs[b]	25 Years	Current practice	€451.7 B	€262.5 B	€21.0 B	€106.2 B	£52.9 B	$78.0 B	R$469.2 B	¥6.5 T	$3.4 T
		25% increased diagnosis + 75% adherence to GDMT	€288.6 B	€162.5 B	€17.2 B	€62.1 B	£39.9 B	$42.8 B	R$286.1 B	¥3.7 T	$2.4 T
		% Change	**-36.1%**	**-38.1%**	**-18.2%**	**-41.5%**	**-24.5%**	**-45.1%**	**-39.0%**	**-43.4%**	**-28.9%**
	10 Years	Current practice	€145.7 B	€83.7 B	€8.6 B	€32.2 B	£18.2 B	$27.3 B	R$132.9 B	¥1.6 T	$1.2 T
		25% increased diagnosis + 75% adherence to GDMT	€131.2 B	€76.0 B	€7.1 B	€27.7 B	£17.4 B	$24.0 B	R$114.5 B	¥1.3 T	$1.1 T
		% Change	**-10.0%**	**-9.1%**	**-17.5%**	**-13.9%**	**-4.3%**	**-12.3%**	**-13.9%**	**-19.3%**	**-8.2%**
Total Costs of non-KRT CKD & KRT[b]	25 Years	Current practice	€1.4 T	€883.1 B	€86.1 B	€296.9 B	£149.1 B	$232.0 B	R$1.6 T	¥79.4 T	$11.2 T
		25% increased diagnosis + 75% adherence to GDMT	€1.3 T	€803.5 B	€73.3 B	€256.0 B	£137.0 B	$191.5 B	R$1.5 T	¥78.3 T	$10.8 T
		% Change	**-10.2%**	**-9.0%**	**-14.9%**	**-13.8%**	**-8.1%**	**-17.5%**	**-7.4%**	**-1.4%**	**-3.9%**
	10 Years	Current practice	€521.8 B	€323.8 B	€30.5 B	€101.2 B	£56.5 B	$84.6 B	R$601.3 B	¥29.3 T	$4.4 T
		25% increased diagnosis + 75% adherence to GDMT	€506.8 B	€317.1 B	€26.8 B	€97.2 B	£56.0 B	$78.9 B	R$592.0 B	¥29.1 T	$4.4 T
		% Change	**-2.9%**	**-2.1%**	**-11.9%**	**-3.9%**	**-1.0%**	**-6.7%**	**-1.6%**	**-0.7%**	**-0.2%**

Outcome	Time Horizon	Scenario	EUR	Germany	Netherlands	Spain	UK	Australia	Brazil	China	US
Total Costs of non-KRT CKD & KRT Including Treatment Costs[c]	25 Years	Current practice	€1.4 T	€883.1 B	€86.1 B	€296.855 B	£149.1 B	$232.0 B	R$1.6 T	¥79.4 T	$11.2 T
		25% increased diagnosis + 75% adherence to GDMT	€1.4 T	€868.3	€82.0 B	€279.7 B	£163.4 B	$203.0 B	R$1.9 T	¥83.5 T	$11.9 T
		% Change	**-1.3%**	**-1.7%**	**-4.9%**	**-5.8%**	**9.6%**	**-12.5%**	**18.5%**	**5.1%**	**6.7%**
	10 Years	Current practice	€521.8 B	€323.8 B	€30.5 B	€101.2 B	£56.5 B	$84.6 B	R$601.3 B	¥29.3 T	$4.4 T
		25% increased diagnosis + 75% adherence to GDMT	€554.0 B	€340.6 B	€30.1 B	€105.6	£66.3 B	$83.4 B	R$738.9 B	¥31.1 T	$4.9 T
		% Change	**6.2%**	**5.2%**	**-1.2%**	**4.3%**	**17.3%**	**-1.4%**	**22.9%**	**6.2%**	**10.9%**
**Cumulative Environmental Burden**											
All CKD (incl. KRT) Freshwater Consumption (m^3)	25 Years	Current practice	2.5 B	969.5 M	210.5 M	732.9 M	576.2 M	283.4 M	10.6 B	18.9 B	87.7 B
		25% increased diagnosis + 75% adherence to GDMT	2.4 B	877.5 M	211.7 M	703.0 M	564.3 M	263.2 M	10.2 B	18.1 B	89.4 B
		% Change	**-5.3%**	**-9.5%**	**0.6%**	**-4.1%**	**-2.1%**	**-7.1%**	**-3.2%**	**-4.6%**	**1.9%**
	10 Years	Current practice	960.9 M	375.7 M	81.9 M	270.2 M	233.0 M	107.0 M	4.2 B	7.0 B	36.4 B
		25% increased diagnosis + 75% adherence to GDMT	955.6 M	370.3 M	81.9 M	268.8 M	234.7 M	105.3 M	4.2 B	6.9 B	36.9 B
		% Change	**-0.5%**	**-1.5%**	**0.0%**	**-0.5%**	**0.7%**	**-1.6%**	**-0.2%**	**-1.1%**	**1.4%**
All CKD (incl. KRT) Fossil Fuel Depletion (kg oil eq)	25 Years	Current practice	192.5 B	71.1 B	14.8 B	53.3 B	53.4 B	76.4 B	176.7 B	1.7 T	1.3 T
		25% increased diagnosis + 75% adherence to GDMT	189.2 B	68.2 B	15.0 B	52.6 B	53.4 B	73.7 B	173.2 B	1.7 T	1.3 T
		% Change	**-1.7%**	**-4.1%**	**1.7%**	**-1.2%**	**0.0%**	**-3.6%**	**-2.0%**	**-2.4%**	**2.2%**
	10 Years	Current practice	76.0 B	28.5 B	5.7 B	19.9 B	21.8 B	29.0 B	70.0 B	639.4 B	530.9 B
		25% increased diagnosis + 75% adherence to GDMT	76.3 B	28.5 B	5.8 B	20.0 B	22.0 B	28.8 B	70.1 B	637.0 B	538.9 B
		% Change	**0.4%**	**-0.2%**	**1.1%**	**0.3%**	**1.1%**	**-0.7%**	**0.2%**	**-0.4%**	**1.5%**

Outcome	Time Horizon	Scenario	EUR	Germany	Netherlands	Spain	UK	Australia	Brazil	China	US
All CKD (incl. KRT) Carbon Footprint (kg CO_2 eq)	25 Years	Current practice	467.2 B	186.3 B	37.4 B	122.7 B	120.7 B	25.9 B	496.6 B	4.7 T	3.4 T
		25% increased diagnosis + 75% adherence to GDMT	458.0 B	178.0 B	38.0 B	121.1 B	120.8 B	25.0 B	485.2 B	4.5 B	3.4 T
		% Change	**-2.0%**	**-4.5%**	**1.6%**	**-1.3%**	**0.1%**	**-3.4%**	**-2.3%**	**-2.6%**	**0.2%**
	10 Years	Current practice	184.5 B	74.7 B	14.5 B	46.0 B	49.3 B	9.8 B	196.3 B	1.7 T	1.4 T
		25% increased diagnosis + 75% adherence to GDMT	185.2 B	74.5 B	14.7 B	46.1 B	49.9 B	9.8 B	196.4 B	1.7 T	1.4 T
		% Change	**0.3%**	**-0.2%**	**1.0%**	**0.3%**	**1.1%**	**-0.7%**	**0.1%**	**-0.4%**	**-0.7%**
KRT only Freshwater Consumption (m^3)	25 Years	Current practice	486.4 M	275.3 M	17.8 M	112.4 M	80.9 M	50.2 M	1.7 B	2.7 B	6.9 B
		25% increased diagnosis + 75% adherence to GDMT	297.8 M	161.3 M	14.1 M	64.9 M	57.4 M	25.4 M	941.5 M	1.5 B	4.7 B
		% Change	**-38.8%**	**-41.4%**	**-20.9%**	**-42.2%**	**-29.0%**	**-49.5%**	**-43.4%**	**-46.3%**	**-32.2%**
	10 Years	Current practice	156.6 M	87.2 M	7.5 M	34.0 M	28.0 M	17.6 M	461.4 M	665.0 M	2.5 B
		25% increased diagnosis + 75% adherence to GDMT	140.3 M	78.5 M	6.0 M	29.2 M	26.6 M	15.3 M	388.7 M	524.9 M	2.3 B
		% Change	**-10.4%**	**-10.0%**	**-19.6%**	**-14.2%**	**-5.0%**	**-13.5%**	**-15.8%**	**-21.1%**	**-8.9%**
KRT only Fossil Fuel Depletion (kg oil eq)	25 Years	Current practice	21.9 B	12.0 B	649.4 M	5.0 B	4.2 B	8.5 B	23.3 B	171.3 B	95.7 B
		25% increased diagnosis + 75% adherence to GDMT	13.9 B	7.2 B	539.2 M	3.1 B	3.1 B	4.5 B	13.3 B	92.8 B	65.1 B
		% Change	**-36.8%**	**-40.3%**	**-17.0%**	**-39.5%**	**-26.5%**	**-46.8%**	**-42.9%**	**-45.8%**	**-32.0%**
	10 Years	Current practice	7.1 B	3.8 B	261.1 M	1.5 B	1.5 B	3.0 B	6.5 B	41.8 B	34.6 B
		25% increased diagnosis + 75% adherence to GDMT	6.4 B	3.4 B	217.9 M	1.3 B	1.4 B	2.6 B	5.5 B	33.1 B	31.6 B
		% Change	**-9.7%**	**-9.7%**	**-16.6%**	**-13.1%**	**-4.6%**	**-12.8%**	**-15.6%**	**-20.8%**	**-8.9%**

Outcome	Time Horizon	Scenario	EUR	Germany	Netherlands	Spain	UK	Australia	Brazil	China	US
KRT only Carbon Footprint (kg CO_2 eq)	25 Years	Current practice	56.3 B	33.0 B	1.8 B	12.0 B	9.5 B	2.8 B	68.5 B	485.0 B	231.2 B
		25% increased diagnosis + 75% adherence to GDMT	35.4 B	19.7 B	1.5 B	7.2 B	7.0 B	1.5 B	39.0 B	262.4 B	157.3 B
		% Change	**-37.2%**	**-40.5%**	**-17.6%**	**-39.7%**	**-26.5%**	**-46.7%**	**-43.1%**	**-45.9%**	**-32.0%**
	10 Years	Current practice	18.1 B	10.5 B	714.9 M	3.7 B	3.3 B	991.2 M	19.0 B	118.2 B	83.8 B
		25% increased diagnosis + 75% adherence to GDMT	16.3 B	9.4 B	593.2 M	3.2 B	3.1 B	864.6 M	16.0 B	93.6 B	76.3 B
		% Change	**-9.8%**	**-9.8%**	**-17.0%**	**-13.2%**	**-4.6%**	**-12.8%**	**-15.6%**	**-20.8%**	**-8.9%**

[a]Currency conversion for the UK from GBP (£) to euro (€) was performed prior to aggregation across European countries using the 2022 annual average from ECB.[26] Values used in conversions were as follows: £1.0 = €1.173.

[b]Does not include costs associated with CKD treatment or screening.

[c]Does not include costs associated with CKD screening.

Abbreviations: B = billion; CKD = chronic kidney disease; CV = cardiovascular; eq = equivalent; ECB = European Central Bank; EUR = Europe; GBP = pound sterling; GDMT = guideline-directed medical therapy; HHF = hospitalization from heart failure; incl. = including; KRT = kidney replacement therapy; M = million; MI = myocardial infarction; T = trillion; UK = United Kingdoms; US = United States.

Supplementary Table S14. CKD burden over 10 and 25 years following annual targeted screening of high-risk population and an increase to GDMT adherence to 75% compared to current practice.

Outcome	Time Horizon	Scenario	EUR	Germany	Netherlands	Spain	UK	Australia	Brazil	China	US
**Clinical Burden - Cumulative CKD and KRT prevalence**											
CKD stage 1-2	25 Years	Current practice	396.6 M	151.4 M	32.4 M	96.1 M	116.6 M	54.2 M	394.4 M	3.5 B	838.8 M
		Targeted screening + 75% adherence to GDMT	414.1 M	158.0 M	34.0 M	101.1 M	121.0 M	57.0 M	409.8 M	3.6 B	884.5 M
		% Change	**4.4%**	**4.3%**	**4.8%**	**5.2%**	**3.8%**	**5.2%**	**3.9%**	**3.7%**	**5.5%**
	10 Years	Current practice	178.6 M	70.6 M	14.2 M	42.5 M	51.2 M	21.3 M	159.6 M	1.4 B	360.9 M
		Targeted screening + 75% adherence to GDMT	181.8 M	71.9 M	14.5 M	43.4 M	52.0 M	21.7 M	162.0 M	1.4 B	369.0 M
		% Change	**1.8%**	**1.9%**	**1.8%**	**1.9%**	**1.6%**	**1.9%**	**1.5%**	**1.4%**	**2.2%**
CKD stage 3-5 (incl. KRT)	25 Years	Current practice	391.5 M	151.7 M	35.1 M	93.4 M	111.3 M	31.2 M	326.1 M	1.8 B	649.0 M
		Targeted screening + 75% adherence to GDMT	394.0 M	152.9 M	35.5 M	94.1 M	111.6 M	30.0 M	334.7 M	1.8 B	650.5 M
		% Change	**0.6%**	**0.8%**	**1.1%**	**0.7%**	**0.2%**	**-3.8%**	**2.6%**	**-1.4%**	**0.2%**
	10 Years	Current practice	145.3 M	57.0 M	12.7 M	31.8 M	43.9 M	11.6 M	136.4 M	695.4 M	265.9 M
		Targeted screening + 75% adherence to GDMT	145.3 M	56.9 M	12.7 M	31.8 M	43.9 M	11.4 M	137.5 M	689.2 M	263.9 M
		% Change	**0.0%**	**-0.1%**	**0.4%**	**-0.1%**	**0.1%**	**-1.9%**	**0.8%**	**-0.9%**	**-0.8%**
Dialysis	25 Years	Current practice	8.5 M	4.9 M	0.2 M	1.8 M	1.6 M	0.7 M	10.1 M	59.5 M	26.9 M
		Targeted screening + 75% adherence to GDMT	4.8 M	2.7 M	0.1 M	0.9 M	1.0 M	0.3 M	5.3 M	29.1 M	17.0 M
		% Change	**-43.8%**	**-45.5%**	**-27.2%**	**-48.9%**	**-34.7%**	**-55.0%**	**-47.5%**	**-51.1%**	**-37.0%**
	10 Years	Current practice	2.7 M	1.5 M	0.1 M	0.5 M	0.5 M	0.2 M	2.8 M	14.3 M	9.8 M
		Targeted screening + 75% adherence to GDMT	2.4 M	1.4 M	0.1 M	0.5 M	0.5 M	0.2 M	2.3 M	11.2 M	8.9 M
		% Change	**-11.3%**	**-11.0%**	**-23.2%**	**-16.1%**	**-5.6%**	**-14.8%**	**-16.3%**	**-21.9%**	**-10.0%**

Outcome	Time Horizon	Scenario	EUR	Germany	Netherlands	Spain	UK	Australia	Brazil	China	US
Transplant	25 Years	Current practice	5.8 M	2.0 M	0.5 M	1.5 M	1.8 M	0.5 M	3.3 M	8.6 M	12.1 M
		Targeted screening + 75% adherence to GDMT	5.8 M	2.0 M	0.5 M	1.5 M	1.8 M	0.5 M	3.3 M	8.6 M	12.1 M
		% Change	**0.0%**	**0.1%**	**-0.2%**	**0.4%**	**-0.3%**	**-0.5%**	**0.8%**	**0.3%**	**0.2%**
	10 Years	Current practice	1.9 M	0.6 M	0.2 M	0.5 M	0.6 M	0.2 M	1.0 M	2.8 M	4.0 M
		Targeted screening + 75% adherence to GDMT	1.9 M	0.6 M	0.2 M	0.5 M	0.6 M	0.2 M	1.0 M	2.8 M	4.0 M
		% Change	**-0.1%**	**0.0%**	**-0.1%**	**-0.3%**	**-0.3%**	**0.0%**	**0.1%**	**0.0%**	**-0.1%**
**Clinical Burden – Cumulative number of clinical events**											
CV events (MI, stroke, HHF)	25 Years	Current practice	12.2 M	5.1 M	1.1 M	2.3 M	3.7 M	2.1 M	11.6 M	59.6 M	41.2 M
		Targeted screening + 75% adherence to GDMT	6.9 M	2.9 M	0.6 M	1.3 M	2.0 M	1.1 M	6.3 M	37.3 M	22.3 M
		% Change	**-43.8%**	**-43.1%**	**-45.2%**	**-42.6%**	**-45.0%**	**-48.1%**	**-46.0%**	**-37.3%**	**-45.8%**
	10 Years	Current practice	4.1 M	1.8 M	0.3 M	0.7 M	1.3 M	0.5 M	3.3 M	17.5 M	10.9 M
		Targeted screening + 75% adherence to GDMT	2.6 M	1.1 M	0.2 M	0.5 M	0.8 M	0.3 M	2.0 M	12.2 M	6.4 M
		% Change	**-37.8%**	**-38.5%**	**-37.6%**	**-35.6%**	**-37.9%**	**-39.9%**	**-39.2%**	**-30.2%**	**-41.4%**
Death Events	25 Years	Current practice	27.6 M	12.2 M	2.0 M	5.2 M	8.2 M	1.8 M	18.8 M	112.9 M	38.7 M
		Targeted screening + 75% adherence to GDMT	26.3 M	11.7 M	1.9 M	4.7 M	8.0 M	1.7 M	17.2 M	104.2 M	35.3 M
		% Change	**-4.6%**	**-3.9%**	**-6.5%**	**-8.0%**	**-3.1%**	**-7.3%**	**-8.5%**	**-7.7%**	**-8.9%**
	10 Years	Current practice	10.2 M	5.0 M	0.6 M	1.6 M	3.0 M	0.5 M	6.0 M	34.3 M	12.6 M
		Targeted screening + 75% adherence to GDMT	9.7 M	4.8 M	0.6 M	1.5 M	2.9 M	0.5 M	5.4 M	32.1 M	11.4 M
		% Change	**-5.1%**	**-4.2%**	**-8.7%**	**-8.3%**	**-4.1%**	**-7.0%**	**-9.3%**	**-6.5%**	**-9.2%**

Outcome	Time Horizon	Scenario	EUR	Germany	Netherlands	Spain	UK	Australia	Brazil	China	US
**Cumulative Economic Burden[a]**											
Non-KRT CKD Costs[b]	25 Years	Current practice	€989.2 B	€620.6 B	€65.2 B	€190.7 B	£96.2 B	$154.1 B	R$1.1 T	¥73.0 T	$7.8 T
		Targeted screening + 75% adherence to GDMT	€1.0 T	€648.5 B	€53.6 B	€189.7 B	£97.6 B	$142.0 B	R$1.2 T	¥74.5 T	$8.3 T
		% Change	**1.7%**	**4.5%**	**-17.7%**	**-0.5%**	**1.5%**	**-7.8%**	**6.6%**	**2.1%**	**6.1%**
	10 Years	Current practice	€376.0 B	€240.1 B	€21.9 B	€69.1 B	£38.3 B	$57.2 B	R$468.4 B	¥27.7 T	$3.2 T
		Targeted screening + 75% adherence to GDMT	€380.2 B	€245.3 B	€19.3 B	€69.6 B	£39.2 B	$54.8 B	R$486.0 B	¥28.3 T	$3.3 T
		% Change	**1.1%**	**2.2%**	**-11.7%**	**0.8%**	**2.3%**	**-4.3%**	**3.8%**	**2.0%**	**4.0%**
CKD Treatment Costs	25 Years	Incremental Treatment Costs	€210.4 B	€111.4 B	€12.1 B	€38.2 B	£41.5 B	$23.1 B	R$754.9 B	¥11.2 T	$2.2 T
	10 Years	Incremental Treatment Costs	€80.6 B	€42.6 B	€4.8 B	€13.9 B	£16.4 B	$8.8 B	R$245.6 B	¥4.2 T	$928.8 B
KRT costs[b]	25 Years	Current practice	€451.7 B	€262.5 B	€21.0 B	€106.2 B	£52.9 B	$78.0 B	R$469.2 B	¥6.5 T	$3.4 T
		Targeted screening + 75% adherence to GDMT	€279.9 B	€157.1 B	€16.9 B	€60.0 B	£39.1 B	$41.6 B	R$279.6 B	¥3.4 T	$2.4 T
		% Change	**-38.0%**	**-40.1%**	**-19.4%**	**-43.5%**	**-26.0%**	**-46.6%**	**-40.4%**	**-46.8%**	**-29.8%**
	10 Years	Current practice	€145.7 B	€83.7 B	€8.6 B	€32.2 B	£18.2 B	$27.3 B	R$132.9 B	¥1.6 T	$1.2 T
		Targeted screening + 75% adherence to GDMT	€130.7 B	€75.6 B	€7.1 B	€27.6 B	£17.4 B	$23.9 B	R$115.0 B	¥1.3 T	$1.1 T
		% Change	**-10.3%**	**-9.6%**	**-17.7%**	**-14.1%**	**-4.3%**	**-12.5%**	**-13.5%**	**-19.4%**	**-8.2%**
Total Costs of non-KRT CKD & KRT[b]	25 Years	Current practice	€1.4 T	€883.1 B	€86.1 B	€296.9 B	£149.1 B	$232.0 B	R$1.6 T	¥79.4 T	$11.2 T
		Targeted screening + 75% adherence to GDMT	€1.3 T	€805.7 B	€70.6 B	€249.6 B	£136.7 B	$183.7 B	R$1.5 B	¥77.9 T	$10.7 T
		% Change	**-10.7%**	**-8.8%**	**-18.1%**	**-15.9%**	**-8.3%**	**-20.9%**	**-7.4%**	**-1.9%**	**-4.7%**
	10 Years	Current practice	€521.8 B	€323.8 B	€30.5 B	€101.2 B	£56.5 B	$84.6 B	601.3 B	¥29.3 T	$4.4 T
		Targeted screening + 75% adherence to GDMT	€511.0 B	€321.0 B	€26.4 B	€97.2 B	£56.6 B	$78.7 B	600.9 B	¥29.6 T	$4.5 T
		% Change	**-2.1%**	**-0.9%**	**-13.4%**	**-3.9%**	**0.1%**	**-7.0%**	**-0.1%**	**0.9%**	**0.7%**

Outcome	Time Horizon	Scenario	EUR	Germany	Netherlands	Spain	UK	Australia	Brazil	China	US
Total Costs of non-KRT CKD & KRT Including Treatment Costs[c]	25 Years	Current practice	€1.4 T	€883.1 B	€86.1 B	€296.9 B	£149.1 B	$232.0 B	R$1.6 T	¥79.4 T	$11.2 T
		Targeted screening + 75% adherence to GDMT	€1.5 T	€917.1 B	€82.6 B	€287.8 B	£178.2 B	$206.8 B	R$2.2 T	¥89.1 T	$12.9 T
		% Change	**3.9%**	**3.9%**	**-4.1%**	**-3.1%**	**19.6%**	**-10.9%**	**40.6%**	**12.1%**	**15.1%**
	10 Years	Current practice	€521.8 B	€323.8 B	€30.5 B	€101.2	£56.5 B	$84.6 B	R$601.3 B	¥29.3 T	$4.4 T
		Targeted screening + 75% adherence to GDMT	€591.6 B	€363.6 B	€31.2 B	€111.1 B	£73.0 B	$87.5 B	R$846.5 B	¥33.7 T	$5.4 T
		% Change	**13.4%**	**12.3%**	**2.5%**	**9.8%**	**29.2%**	**3.4%**	**40.8%**	**15.1%**	**21.7%**
**Cumulative Environmental Burden**											
All CKD (incl. KRT) Freshwater Consumption (m^3)	25 Years	Current practice	2.5 B	969.5 M	210.5 M	732.9 M	576.2 M	283.4 M	10.6 B	18.9 B	87.7 B
		Targeted screening + 75% adherence to GDMT	2.4 B	904.9 M	219.4 M	730.9 M	584.2 M	274.2 M	10.7 B	18.9 B	93.8 B
		% Change	**-2.0%**	**-6.7%**	**4.2%**	**-0.3%**	**1.4%**	**-3.2%**	**0.8%**	**-0.5%**	**6.9%**
	10 Years	Current practice	960.9 M	375.7 M	81.9 M	270.2 M	233.0 M	107.0 M	4.2 B	7.0 B	36.4 B
		Targeted screening + 75% adherence to GDMT	987.4 M	381.9 M	84.5 M	278.5 M	242.5 M	109.4 M	4.3 B	7.2 B	38.5 B
		% Change	**2.8%**	**1.6%**	**3.1%**	**3.1%**	**4.0%**	**2.3%**	**3.2%**	**3.0%**	**5.7%**
All CKD (incl. KRT) Fossil Fuel Depletion (kg oil eq)	25 Years	Current practice	192.5 B	71.1 B	14.8 B	53.3 B	53.4 B	76.4 B	176.7 B	1.7 T	1.3 T
		Targeted screening + 75% adherence to GDMT	197.0 B	71.0 B	15.6 B	54.9 B	55.5 B	76.9 B	181.0 B	1.7 T	1.4 T
		% Change	**2.3%**	**-0.1%**	**5.6%**	**3.1%**	**3.9%**	**0.6%**	**2.4%**	**2.2%**	**7.2%**
	10 Years	Current practice	76.0 B	28.5 B	5.7 B	19.9 B	21.8 B	29.0 B	70.0 B	639.4 B	530.9 B
		Targeted screening + 75% adherence to GDMT	79.1 B	29.6 B	6.0 B	20.8 B	22.8 B	30.0 B	72.7 B	663.8 B	561.6 B
		% Change	**4.1%**	**3.6%**	**4.4%**	**4.2%**	**4.6%**	**3.4%**	**3.8%**	**3.8%**	**5.8%**

Outcome	Time Horizon	Scenario	EUR	Germany	Netherlands	Spain	UK	Australia	Brazil	China	US
All CKD (incl. KRT) Carbon Footprint (kg CO_2 eq)	25 Years	Current practice	467.2 B	186.3 B	37.4 B	122.7 B	120.7 B	25.9 B	496.6 B	4.7 T	3.4 T
		Targeted screening + 75% adherence to GDMT	476.8 B	185.4 B	39.5 B	126.4 B	125.5 B	26.1 B	507.0 B	4.8 T	3.6 T
		% Change	**2.1%**	**-0.5%**	**5.6%**	**3.0%**	**4.0%**	**0.8%**	**2.1%**	**2.0%**	**3.6%**
	10 Years	Current practice	184.5 B	74.7 B	14.5 B	46.0 B	49.3 B	9.8 B	196.3 B	1.7 T	1.4 T
		Targeted screening + 75% adherence to GDMT	192.0 B	77.4 B	15.2 B	47.9 B	51.6 B	10.2 B	203.5 B	1.8 T	1.5 T
		% Change	**4.1%**	**3.5%**	**4.4%**	**4.2%**	**4.7%**	**3.5%**	**3.7%**	**3.8%**	**2.6%**
KRT only Freshwater Consumption (m^3)	25 Years	Current practice	486.4 M	275.3 M	17.8 M	112.4 M	80.9 M	50.2 M	1.7 B	2.7 B	6.9 B
		Targeted screening + 75% adherence to GDMT	287.6 M	155.3 M	13.8 M	62.6 M	55.9 M	24.6 M	916.0 M	1.4 B	4.6 B
		% Change	**-40.9%**	**-43.6%**	**-22.2%**	**-44.3%**	**-30.9%**	**-51.0%**	**-45.0%**	**-50.0%**	**-33.2%**
	10 Years	Current practice	156.6 M	87.2 M	7.5 M	34.0 M	28.0 M	17.6 M	461.4 M	665.0 M	2.5 B
		Targeted screening + 75% adherence to GDMT	139.7 M	78.0 M	6.0 M	29.1 M	26.6 M	15.2 M	390.6 M	524.0 M	2.3 B
		% Change	**-10.8%**	**-10.5%**	**-19.8%**	**-14.4%**	**-5.0%**	**-13.7%**	**-15.4%**	**-21.2%**	**-9.0%**
KRT only Fossil Fuel Depletion (kg oil eq)	25 Years	Current practice	21.9 B	12.0 B	649.4 M	5.0 B	4.2 B	8.5 B	23.3 B	171.3 B	95.7 B
		Targeted screening + 75% adherence to GDMT	13.4 B	6.9 B	532.4 M	3.0 B	3.0 B	4.4 B	12.9 B	86.6 B	64.2 B
		% Change	**-38.8%**	**-42.5%**	**-18.0%**	**-41.4%**	**-28.2%**	**-48.2%**	**-44.5%**	**-49.5%**	**-33.0%**
	10 Years	Current practice	7.1 B	3.8 B	261.1 M	1.5 B	1.5 B	3.0 B	6.5 B	41.8 B	34.6 B
		Targeted screening + 75% adherence to GDMT	6.3 B	3.4 B	217.4 M	1.3 B	1.4 B	2.6 B	5.5 B	33.0 B	31.5 B
		% Change	**-10.0%**	**-10.2%**	**-16.8%**	**-13.3%**	**-4.6%**	**-13.0%**	**-15.2%**	**-20.9%**	**-9.0%**

Outcome	Time Horizon	Scenario	EUR	Germany	Netherlands	Spain	UK	Australia	Brazil	China	US
KRT only Carbon Footprint (kg CO_2 eq)	25 Years	Current practice	56.3 B	33.0 B	1.8 B	12.0 B	9.5 B	2.8 B	68.5 B	485.0 B	231.2 B
		Targeted screening + 75% adherence to GDMT	34.2 B	19.0 B	1.4 B	7.0 B	6.8 B	1.5 B	37.9 B	244.8 B	154.9 B
		% Change	**-39.2%**	**-42.6%**	**-18.7%**	**-41.7%**	**-28.2%**	**-48.1%**	**-44.6%**	**-49.5%**	**-33.0%**
	10 Years	Current practice	18.1 B	10.5 B	714.9 M	3.7 B	3.3 B	991.2 M	19.0 B	118.2 B	83.8 B
		Targeted screening + 75% adherence to GDMT	16.3 B	9.4 B	591.7 M	3.2 B	3.1 B	862.6 M	16.1 B	93.4 B	76.2 B
		% Change	**-10.2%**	**-10.3%**	**-17.2%**	**-13.4%**	**-4.6%**	**-13.0%**	**-15.2%**	**-21.0%**	**-9.0%**

[a]Currency conversion for the UK from GBP (£) to euro (€) was performed prior to aggregation across European countries using the 2022 annual average from ECB.[26] Values used in conversions were as follows: £1.0 = €1.173.

[b]Does not include costs associated with CKD treatment or screening.

[c]Does not include costs associated with CKD screening.

Abbreviations: B = billion; CKD = chronic kidney disease; CV = cardiovascular; eq = equivalent; ECB = European Central Bank; EUR = Europe; GBP = pound sterling; GDMT = guideline-directed medical therapy; HHF = hospitalization from heart failure; incl. = including; KRT = kidney replacement therapy; M = million; MI = myocardial infarction; T = trillion; UK = United Kingdoms; US = United States.

Supplementary Table S15. CKD burden over 10 and 25 years following an increase to 60% adherence to GDMT compared to current practice.

Outcome	Time Horizon	Scenario	EUR	Germany	Netherlands	Spain	UK	Australia	Brazil	China	US
**Clinical Burden - Cumulative CKD and KRT prevalence**											
CKD stage 1-2	25 Years	Current practice	396.6 M	151.4 M	32.4 M	96.1 M	116.6 M	54.2 M	394.4 M	3.5 B	838.8 M
		60% adherence to GDMT	400.1 M	152.7 M	32.8 M	97.1 M	117.5 M	54.8 M	397.3 M	3.5 B	846.5 M
		% Change	**0.9%**	**0.8%**	**1.0%**	**1.0%**	**0.8%**	**1.0%**	**0.7%**	**0.7%**	**0.9%**
	10 Years	Current practice	178.6 M	70.6 M	14.2 M	42.5 M	51.2 M	21.3 M	159.6 M	1.4 B	360.9 M
		60% adherence to GDMT	179.3 M	70.9 M	14.3 M	42.7 M	51.4 M	21.3 M	160.2 M	1.4 B	362.5 M
		% Change	**0.4%**	**0.4%**	**0.4%**	**0.5%**	**0.4%**	**0.4%**	**0.4%**	**0.3%**	**0.4%**
CKD stage 3-5 (incl. KRT)	25 Years	Current practice	391.5 M	151.7 M	35.1 M	93.4 M	111.3 M	31.2 M	326.1 M	1.8 B	649.0 M
		60% adherence to GDMT	398.2 M	154.2 M	35.8 M	95.3 M	112.9 M	31.4 M	336.1 M	1.8 B	665.2 M
		% Change	**1.7%**	**1.7%**	**2.0%**	**2.1%**	**1.4%**	**0.7%**	**3.1%**	**1.3%**	**2.5%**
	10 Years	Current practice	145.3 M	57.0 M	12.7 M	31.8 M	43.9 M	11.6 M	136.4 M	695.4 M	265.9 M
		60% adherence to GDMT	146.3 M	57.3 M	12.8 M	32.0 M	44.1 M	11.6 M	137.7 M	696.9 M	267.3 M
		% Change	**0.6%**	**0.6%**	**0.8%**	**0.7%**	**0.6%**	**-0.1%**	**0.9%**	**0.2%**	**0.5%**
Dialysis	25 Years	Current practice	8.5 M	4.9 M	197.9 K	1.8 M	1.6 M	705.4 K	10.1 M	59.5 M	26.9 M
		60% adherence to GDMT	5.9 M	3.3 M	164.4 K	1.2 M	1.2 M	409.7 K	6.7 M	41.1 M	19.8 M
		% Change	**-30.6%**	**-32.9%**	**-16.9%**	**-33.5%**	**-21.6%**	**-41.9%**	**-34.1%**	**-31.0%**	**-26.6%**
	10 Years	Current practice	2.7 M	1.5 M	86.8 K	538.4 K	544.0 K	248.7 K	2.8 M	14.3 M	9.8 M
		60% adherence to GDMT	2.5 M	1.4 M	72.4 K	469.2 K	532.1 K	222.1 K	2.5 M	12.8 M	9.2 M
		% Change	**-7.5%**	**-7.0%**	**-16.7%**	**-12.9%**	**-2.2%**	**-10.7%**	**-9.8%**	**-10.8%**	**-6.4%**

Outcome	Time Horizon	Scenario	EUR	Germany	Netherlands	Spain	UK	Australia	Brazil	China	US
Transplant	25 Years	Current practice	5.8 M	2.0 M	529.7 K	1.5 M	1.8 M	539.1 K	3.3 M	8.6 M	12.1 M
		60% adherence to GDMT	5.8 M	2.0 M	527.7 K	1.5 M	1.8 M	537.4 K	3.3 M	8.6 M	12.1 M
		% Change	**-0.1%**	**0.0%**	**-0.4%**	**0.5%**	**-0.6%**	**-0.3%**	**0.4%**	**0.3%**	**-0.2%**
	10 Years	Current practice	1.9 M	649.0 K	172.5 K	513.6 K	576.1 K	178.4 K	1.0 M	2.8 M	4.0 M
		60% adherence to GDMT	1.9 M	649.2 K	172.3 K	513.3 K	574.3 K	178.2 K	1.0 M	2.8 M	4.0 M
		% Change	**-0.1%**	**0.0%**	**-0.1%**	**-0.1%**	**-0.3%**	**-0.1%**	**-0.1%**	**0.1%**	**-0.1%**
**Clinical Burden – Cumulative number of clinical events**											
CV events (MI, stroke, HHF)	25 Years	Current practice	12.2 M	5.1 M	1.1 M	2.3 M	3.7 M	2.1 M	11.6 M	59.6 M	41.2 M
		60% adherence to GDMT	10.3 M	4.3 M	910.6 K	2.0 M	3.1 M	1.8 M	9.7 M	53.9 M	34.5 M
		% Change	**-15.8%**	**-14.8%**	**-18.0%**	**-14.1%**	**-17.6%**	**-16.6%**	**-16.2%**	**-9.6%**	**-16.1%**
	10 Years	Current practice	4.1 M	1.8 M	327.8 K	702.5 K	1.3 M	532.0 K	3.3 M	17.5 M	10.9 M
		60% adherence to GDMT	3.6 M	1.6 M	281.6 K	623.8 K	1.1 M	461.4 K	2.9 M	16.2 M	9.4 M
		% Change	**-13.1%**	**-13.0%**	**-14.1%**	**-11.2%**	**-13.9%**	**-13.3%**	**-13.7%**	**-7.8%**	**-13.7%**
Death Events	25 Years	Current practice	27.6 M	12.2 M	2.0 M	5.2 M	8.2 M	1.8 M	18.8 M	112.9 M	38.7 M
		60% adherence to GDMT	27.0 M	12.0 M	2.0 M	5.0 M	8.1 M	1.8 M	18.0 M	109.2 M	37.0 M
		% Change	**-2.2%**	**-1.7%**	**-3.3%**	**-3.9%**	**-1.5%**	**-3.3%**	**-4.2%**	**-3.3%**	**-4.3%**
	10 Years	Current practice	10.2 M	5.0 M	612.9 K	1.6 M	3.0 M	538.2 K	6.0 M	34.3 M	12.6 M
		60% adherence to GDMT	10.0 M	4.9 M	584.6 K	1.5 M	2.9 M	524.1 K	5.7 M	33.5 M	12.0 M
		% Change	**-2.4%**	**-1.9%**	**-4.6%**	**-4.3%**	**-1.9%**	**-2.6%**	**-4.4%**	**-2.6%**	**-4.2%**

Outcome	Time Horizon	Scenario	EUR	Germany	Netherlands	Spain	UK	Australia	Brazil	China	US
**Cumulative Economic Burden[a]**											
Non-KRT CKD Costs[b]	25 Years	Current practice	€989.2 B	€620.6 B	€65.2 B	€190.7 B	£96.2 B	$154.1 B	R$1.1 T	¥73.0 T	$7.8 T
		60% adherence to GDMT	€986.1 B	€626.8 B	€57.5 B	€190.3 B	£95.0 B	$148.2 B	R$1.1 T	¥72.8 T	$8.1 T
		% Change	**-0.3%**	**1.0%**	**-11.7%**	**-0.2%**	**-1.2%**	**-3.8%**	**2.8%**	**-0.3%**	**3.8%**
	10 Years	Current practice	€376.0 B	€240.1 B	€21.9 B	€69.1 B	£38.3 B	$57.2 B	R$468.4 B	¥27.7 T	$3.2 T
		60% adherence to GDMT	€369.5 B	€236.8 B	€20.0 B	€68.3 B	£37.8 B	$54.6 B	R$466.0 B	¥27.2 T	$3.2 T
		% Change	**-1.7%**	**-1.4%**	**-8.5%**	**-1.1%**	**-1.4%**	**-4.6%**	**-0.5%**	**-1.7%**	**0.4%**
CKD Treatment Costs	25 Years	Incremental Treatment Costs	€136.5 B	€72.2 B	€10.0 B	€23.1 B	£26.6 B	$13.4 B	R$328.8 B	¥5.7 T	$1.6 T
	10 Years	Incremental Treatment Costs	€46.6 B	€24.5 B	€3.2 B	€7.7 B	£9.5 B	$4.5 B	R$112.0 B	¥2.0 T	$572.7 B
KRT costs[b]	25 Years	Current practice	€451.7 B	€262.5 B	€21.0 B	€106.2 B	£52.9 B	$78.0 B	R$469.2 B	¥6.5 T	$3.4 T
		60% adherence to GDMT	€331.1 B	€186.3 B	€18.4 B	€74.5 B	£44.2 B	$50.4 B	R$332.9 B	¥4.6 T	$2.6 T
		% Change	**-26.7%**	**-29.1%**	**-12.2%**	**-29.8%**	**-16.3%**	**-35.4%**	**-29.1%**	**-28.4%**	**-21.5%**
	10 Years	Current practice	€145.7 B	€83.7 B	€8.6 B	€32.2 B	£18.2 B	$27.3 B	R$132.9 B	¥1.6 T	$1.2 T
		60% adherence to GDMT	€135.6 B	€78.6 B	€7.5 B	€28.6 B	£17.8 B	$24.9 B	R$122.1 B	¥1.4 T	$1.1 T
		% Change	**-7.0%**	**-6.1%**	**-12.7%**	**-11.2%**	**-1.7%**	**-9.0%**	**-8.2%**	**-9.5%**	**-5.3%**
Total Costs of non-KRT CKD & KRT[b]	25 Years	Current practice	€1.4 T	€883.1 B	€86.1 B	€296.9 B	£149.1 B	$232.0 B	R$1.6 T	¥79.4 T	$11.2 T
		60% adherence to GDMT	€1.3 T	€813.0 B	€76.0 B	€264.8 B	£139.3 B	$198.6 B	R$1.5 T	¥77.4 T	$10.8 T
		% Change	**-8.6%**	**-7.9%**	**-11.8%**	**-10.8%**	**-6.6%**	**-14.4%**	**-6.7%**	**-2.6%**	**-3.8%**
	10 Years	Current practice	€521.8 B	€323.8 B	€30.5 B	€101.2 B	£56.5 B	$84.6 B	R$601.3 B	¥29.3 T	$4.4 T
		60% adherence to GDMT	€505.0 B	€315.4 B	€27.5 B	€96.8 B	£55.7 B	$79.5 B	R$588.1 B	¥28.7 T	$4.4 T
		% Change	**-3.2%**	**-2.6%**	**-9.7%**	**-4.3%**	**-1.5%**	**-6.0%**	**-2.2%**	**-2.2%**	**-1.2%**

Outcome	Time Horizon	Scenario	EUR	Germany	Netherlands	Spain	UK	Australia	Brazil	China	US
Total Costs of non-KRT CKD & KRT Including Treatment Costs[c]	25 Years	Current practice	€1.4 T	€883.1 B	€86.1 B	€296.9 B	£149.1 B	$232.0 B	R$1.6 T	¥79.4 T	$11.2 T
		60% adherence to GDMT	€1.5 T	€885.3 B	€85.9 B	€288.0 B	£165.9 B	$212.0 B	R$1.8 T	¥83.1 T	$12.3 T
		% Change	**0.9%**	**0.3%**	**-0.3%**	**-3.0%**	**11.3%**	**-8.7%**	**14.2%**	**4.6%**	**10.3%**
	10 Years	Current practice	€521.8 B	€323.8 B	€30.5 B	€101.2 B	£56.5 B	$84.6 B	R$601.3 B	¥29.3 T	$4.4 T
		60% adherence to GDMT	€551.6 B	€339.9 B	€30.8 B	€104.5 B	£65.2 B	$83.9 B	R$700.1 B	¥30.7 T	$4.9 T
		% Change	**5.7%**	**5.0%**	**0.9%**	**3.2%**	**15.4%**	**-0.8%**	**16.4%**	**4.7%**	**11.8%**
**Cumulative Environmental Burden**											
All CKD (incl. KRT) Freshwater Consumption (m^3)	25 Years	Current practice	2.5 B	969.5 M	210.5 M	732.9 M	576.2 M	283.4 M	10.6 B	18.9 B	87.7 B
		60% adherence to GDMT	2.4 B	891.8 M	209.4 M	705.1 M	563.6 M	265.2 M	10.2 B	18.3 B	88.3 B
		% Change	**-4.8%**	**-8.0%**	**-0.5%**	**-3.8%**	**-2.2%**	**-6.4%**	**-3.3%**	**-3.6%**	**0.6%**
	10 Years	Current practice	960.9 M	375.7 M	81.9 M	270.2 M	233.0 M	107.0 M	4.2 B	7.0 B	36.4 B
		60% adherence to GDMT	948.9 M	369.1 M	81.1 M	266.3 M	232.4 M	104.8 M	4.1 B	6.9 B	36.5 B
		% Change	**-1.3%**	**-1.8%**	**-1.1%**	**-1.4%**	**-0.3%**	**-2.0%**	**-1.0%**	**-1.1%**	**0.2%**
All CKD (incl. KRT) Fossil Fuel Depletion (kg oil eq)	25 Years	Current practice	192.5 B	71.1 B	14.8 B	53.3 B	53.4 B	76.4 B	176.7 B	1.7 T	1.3 T
		60% adherence to GDMT	188.3 B	68.3 B	14.8 B	52.3 B	52.9 B	73.6 B	172.5 B	1.7 T	1.3 T
		% Change	**-2.2%**	**-4.0%**	**0.2%**	**-1.8%**	**-0.9%**	**-3.7%**	**-2.4%**	**-2.2%**	**0.8%**
	10 Years	Current practice	76.0 B	28.5 B	5.7 B	19.9 B	21.8 B	29.0 B	70.0 B	639.4 B	530.9 B
		60% adherence to GDMT	75.5 B	28.3 B	5.7 B	19.8 B	21.8 B	28.5 B	69.5 B	634.4 B	532.4 B
		% Change	**-0.6%**	**-0.9%**	**-0.3%**	**-0.8%**	**-0.2%**	**-1.5%**	**-0.8%**	**-0.8%**	**0.3%**

Outcome	Time Horizon	Scenario	EUR	Germany	Netherlands	Spain	UK	Australia	Brazil	China	US
All CKD (incl. KRT) Carbon Footprint (kg CO_2 eq)	25 Years	Current practice	467.2 B	186.3 B	37.4 B	122.7 B	120.7 B	25.9 B	496.6 B	4.7 T	3.4 T
		60% adherence to GDMT	456.1 B	178.4 B	37.5 B	120.4 B	119.7 B	25.0 B	483.6 B	4.6 T	3.4 T
		% Change	**-2.4%**	**-4.3%**	**0.2%**	**-1.9%**	**-0.8%**	**-3.6%**	**-2.6%**	**-2.3%**	**-0.5%**
	10 Years	Current practice	184.5 B	74.7 B	14.5 B	46.0 B	49.3 B	9.8 B	196.3 B	1.7 T	1.4 T
		60% adherence to GDMT	183.3 B	74.0 B	14.5 B	45.6 B	49.3 B	9.7 B	194.7 B	1.7 T	1.4 T
		% Change	**-0.7%**	**-1.0%**	**-0.3%**	**-0.8%**	**-0.2%**	**-1.4%**	**-0.8%**	**-0.8%**	**-1.3%**
KRT only Freshwater Consumption (m^3)	25 Years	Current practice	486.4 M	275.3 M	17.8 M	112.4 M	80.9 M	50.2 M	1.7 B	2.7 B	6.9 B
		60% adherence to GDMT	347.3 M	188.4 M	15.3 M	78.3 M	65.3 M	30.7 M	1.1 B	1.9 B	5.2 B
		% Change	**-28.6%**	**-31.6%**	**-13.9%**	**-30.3%**	**-19.3%**	**-38.8%**	**-32.3%**	**-30.3%**	**-23.9%**
	10 Years	Current practice	156.6 M	87.2 M	7.5 M	34.0 M	28.0 M	17.6 M	461.4 M	665.0 M	2.5 B
		60% adherence to GDMT	145.3 M	81.4 M	6.4 M	30.1 M	27.5 M	15.9 M	418.7 M	595.6 M	2.3 B
		% Change	**-7.2%**	**-6.7%**	**-14.3%**	**-11.5%**	**-2.0%**	**-9.9%**	**-9.3%**	**-10.4%**	**-5.8%**
KRT only Fossil Fuel Depletion (kg oil eq)	25 Years	Current practice	21.9 B	12.0 B	649.4 M	5.0 B	4.2 B	8.5 B	23.3 B	171.3 B	95.7 B
		60% adherence to GDMT	16.0 B	8.3 B	576.0 M	3.6 B	3.5 B	5.4 B	15.9 B	120.0 B	73.0 B
		% Change	**-27.1%**	**-30.7%**	**-11.3%**	**-28.4%**	**-17.6%**	**-36.7%**	**-32.0%**	**-30.0%**	**-23.7%**
	10 Years	Current practice	7.1 B	3.8 B	261.1 M	1.5 B	1.5 B	3.0 B	6.5 B	41.8 B	34.6 B
		60% adherence to GDMT	6.6 B	3.6 B	229.6 M	1.4 B	1.4 B	2.7 B	5.9 B	37.4 B	32.6 B
		% Change	**-6.6%**	**-6.5%**	**-12.1%**	**-10.6%**	**-1.9%**	**-9.4%**	**-9.1%**	**-10.3%**	**-5.8%**

Outcome	Time Horizon	Scenario	EUR	Germany	Netherlands	Spain	UK	Australia	Brazil	China	US
KRT only Carbon Footprint (kg CO_2 eq)	25 Years	Current practice	56.3 B	33.0 B	1.8 B	12.0 B	9.5 B	2.8 B	68.5 B	485.0 B	231.2 B
		60% adherence to GDMT	40.8 B	22.8 B	1.6 B	8.6 B	7.8 B	1.8 B	46.5 B	339.5 B	176.3 B
		% Change	**-27.5%**	**-30.9%**	**-11.7%**	**-28.5%**	**-17.6%**	**-36.6%**	**-32.1%**	**-30.0%**	**-23.7%**
	10 Years	Current practice	18.1 B	10.5 B	714.9 M	3.7 B	3.3 B	991.2 M	19.0 B	118.2 B	83.8 B
		60% adherence to GDMT	16.9 B	9.8 B	626.3 M	3.3 B	3.2 B	898.0 M	17.3 B	106.0 B	78.9 B
		% Change	**-6.7%**	**-6.5%**	**-12.4%**	**-10.7%**	**-1.9%**	**-9.4%**	**-9.2%**	**-10.3%**	**-5.8%**

[a]Currency conversion for the UK from GBP (£) to euro (€) was performed prior to aggregation across European countries using the 2022 annual average from ECB.[26] Values used in conversions were as follows: £1.0 = €1.173.

[b]Does not include costs associated with CKD treatment or screening.

[c]Does not include costs associated with CKD screening.

Abbreviations: B = billion; CKD = chronic kidney disease; CV = cardiovascular; eq = equivalent; ECB = European Central Bank; EUR = Europe; GBP = pound sterling; GDMT = guideline-directed medical therapy; HHF = hospitalization from heart failure; incl. = including; K = thousand; KRT = kidney replacement therapy; M = million; MI = myocardial infarction; T = trillion; UK = United Kingdoms; US = United States.

Supplementary Table S16. CKD burden over 10 and 25 years following an increase to 90% adherence to GDMT compared to current practice.

Outcome	Time Horizon	Scenario	EUR	Germany	Netherlands	Spain	UK	Australia	Brazil	China	US
**Clinical Burden - Cumulative CKD and KRT prevalence**											
CKD stage 1-2	25 Years	Current practice	396.6 M	151.4 M	32.4 M	96.1 M	116.6 M	54.2 M	394.4 M	3.5 B	838.8 M
		90% adherence to GDMT	400.9 M	153.0 M	32.8 M	97.4 M	117.7 M	54.9 M	398.1 M	3.5 B	849.2 M
		% Change	**1.1%**	**1.1%**	**1.2%**	**1.3%**	**0.9%**	**1.2%**	**1.0%**	**0.9%**	**1.2%**
	10 Years	Current practice	178.6 M	70.6 M	14.2 M	42.5 M	51.2 M	21.3 M	159.6 M	1.4 B	360.9 M
		90% adherence to GDMT	179.5 M	71.0 M	14.3 M	42.8 M	51.4 M	21.4 M	160.4 M	1.4 B	363.1 M
		% Change	**0.5%**	**0.5%**	**0.6%**	**0.6%**	**0.5%**	**0.6%**	**0.5%**	**0.4%**	**0.6%**
CKD stage 3-5 (incl. KRT)	25 Years	Current practice	391.5 M	151.7 M	35.1 M	93.4 M	111.3 M	31.2 M	326.1 M	1.8 B	649.0 M
		90% adherence to GDMT	400.9 M	155.3 M	36.1 M	96.0 M	113.5 M	31.6 M	339.5 M	1.9 B	670.4 M
		% Change	**2.4%**	**2.4%**	**2.7%**	**2.8%**	**2.0%**	**1.2%**	**4.1%**	**1.8%**	**3.3%**
	10 Years	Current practice	145.3 M	57.0 M	12.7 M	31.8 M	43.9 M	11.6 M	136.4 M	695.4 M	265.9 M
		90% adherence to GDMT	146.8 M	57.5 M	12.8 M	32.1 M	44.3 M	11.6 M	138.3 M	698.0 M	268.0 M
		% Change	**1.0%**	**1.0%**	**1.2%**	**1.1%**	**0.9%**	**0.0%**	**1.4%**	**0.4%**	**0.8%**
Dialysis	25 Years	Current practice	8.5 M	4.9 M	197.9 K	1.8 M	1.6 M	705.4 K	10.1 M	59.5 M	26.9 M
		90% adherence to GDMT	5.2 M	2.9 M	153.9 K	1.0 M	1.1 M	348.2 K	5.7 M	32.7 M	17.8 M
		% Change	**-38.6%**	**-40.4%**	**-22.2%**	**-43.7%**	**-29.1%**	**-50.6%**	**-44.0%**	**-45.0%**	**-33.7%**
	10 Years	Current practice	2.7 M	1.5 M	86.8 K	538.4 K	544.0 K	248.7 K	2.8 M	14.3 M	9.8 M
		90% adherence to GDMT	2.4 M	1.4 M	68.4 K	455.1 K	519.7 K	214.3 K	2.4 M	11.5 M	8.9 M
		% Change	**-10.3%**	**-9.9%**	**-21.3%**	**-15.5%**	**-4.5%**	**-13.8%**	**-15.3%**	**-20.0%**	**-9.3%**

Outcome	Time Horizon	Scenario	EUR	Germany	Netherlands	Spain	UK	Australia	Brazil	China	US
Transplant	25 Years	Current practice	5.8 M	2.0 M	529.7 K	1.5 M	1.8 M	539.1 K	3.3 M	8.6 M	12.1 M
		90% adherence to GDMT	5.8 M	2.0 M	527.5 K	1.5 M	1.8 M	540.9 K	3.3 M	8.6 M	12.1 M
		% Change	**-0.1%**	**0.0%**	**-0.4%**	**0.5%**	**-0.5%**	**0.3%**	**0.9%**	**-0.3%**	**-0.1%**
	10 Years	Current practice	1.9 M	649.0 K	172.5 K	513.6 K	576.1 K	178.4 K	1.0 M	2.8 M	4.0 M
		90% adherence to GDMT	1.9 M	649.2 K	172.2 K	512.4 K	573.9 K	178.3 K	1.0 M	2.8 M	4.0 M
		% Change	**-0.2%**	**0.0%**	**-0.2%**	**-0.3%**	**-0.4%**	**-0.1%**	**-0.1%**	**-0.2%**	**-0.2%**
**Clinical Burden – Cumulative number of clinical events**											
CV events (MI, stroke, HHF)	25 Years	Current practice	12.2 M	5.1 M	1.1 M	2.3 M	3.7 M	2.1 M	11.6 M	59.6 M	41.2 M
		90% adherence to GDMT	9.2 M	3.9 M	813.3 K	1.8 M	2.7 M	1.6 M	8.6 M	50.2 M	31.3 M
		% Change	**-24.9%**	**-23.9%**	**-26.7%**	**-23.9%**	**-26.2%**	**-23.8%**	**-25.7%**	**-15.8%**	**-24.0%**
	10 Years	Current practice	4.1 M	1.8 M	327.8 K	702.5 K	1.3 M	532.0 K	3.3 M	17.5 M	10.9 M
		90% adherence to GDMT	3.2 M	1.4 M	253.4 K	555.0 K	1.0 M	418.7 K	2.5 M	15.0 M	8.4 M
		% Change	**-22.7%**	**-23.1%**	**-22.7%**	**-21.0%**	**-23.0%**	**-21.3%**	**-24.2%**	**-14.5%**	**-22.6%**
Death Events	25 Years	Current practice	27.6 M	12.2 M	2.0 M	5.2 M	8.2 M	1.8 M	18.8 M	112.9 M	38.7 M
		90% adherence to GDMT	26.8 M	11.9 M	1.9 M	4.9 M	8.1 M	1.7 M	17.8 M	108.3 M	36.6 M
		% Change	**-2.7%**	**-2.2%**	**-3.9%**	**-4.8%**	**-1.9%**	**-4.3%**	**-5.3%**	**-4.1%**	**-5.5%**
	10 Years	Current practice	10.2 M	5.0 M	612.9 K	1.6 M	3.0 M	538.2 K	6.0 M	34.3 M	12.6 M
		90% adherence to GDMT	9.9 M	4.9 M	576.9 K	1.5 M	2.9 M	517.0 K	5.6 M	33.1 M	11.8 M
		% Change	**-3.2%**	**-2.6%**	**-5.9%**	**-5.4%**	**-2.5%**	**-3.9%**	**-6.2%**	**-3.6%**	**-5.9%**

Outcome	Time Horizon	Scenario	EUR	Germany	Netherlands	Spain	UK	Australia	Brazil	China	US
**Cumulative Economic Burden[a]**											
Non-KRT CKD Costs[b]	25 Years	Current practice	€989.2 B	€620.6 B	€65.2 B	€190.7 B	£96.2 B	$154.1 B	R$1.1 T	¥73.0 T	$7.8 T
		90% adherence to GDMT	€990.3 M	€631.0 B	€56.7 B	€190.8 B	£95.4 B	$147.6 B	R$1.1 T	¥73.3 T	$8.2 T
		% Change	**0.1%**	**1.7%**	**-13.0%**	**0.0%**	**-0.8%**	**-4.2%**	**3.9%**	**0.5%**	**4.9%**
	10 Years	Current practice	€376.0 B	€240.1 B	€21.9 B	€69.1 B	£38.3 B	$57.2 B	R$468.4 B	¥27.7 T	$3.2 T
		90% adherence to GDMT	€369.0 B	€236.9 B	€19.6 B	€68.0 B	£37.8 B	$54.1 B	R$467.2 B	¥27.3 T	$3.2 T
		% Change	**-1.9%**	**-1.3%**	**-10.2%**	**-1.5%**	**-1.4%**	**-5.5%**	**-0.3%**	**-1.6%**	**0.6%**
CKD Treatment Costs	25 Years	Incremental Treatment Costs	€98.1 B	€48.6 B	€6.4 B	€19.1 B	£20.6 B	$8.6 B	R$350.3 B	¥3.9 T	$831.4 B
	10 Years	Incremental Treatment Costs	€35.0 B	€16.7 B	€2.4 B	€6.6 B	£7.9 B	$3.2 B	R$125.1 B	¥1.5 T	$323.3 B
KRT costs[b]	25 Years	Current practice	€451.7 B	€262.5 B	€21.0 B	€106.2 B	£52.9 B	$78.0 B	R$469.2 B	¥6.5 T	$3.4 T
		90% adherence to GDMT	€299.7 B	€168.8 B	€17.6 B	€64.9 B	£41.3 B	$44.7 B	R$293.7 B	¥3.8 T	$2.5 T
		% Change	**-33.6%**	**-35.7%**	**-15.9%**	**-38.9%**	**-21.9%**	**-42.7%**	**-37.4%**	**-41.3%**	**-27.2%**
	10 Years	Current practice	€145.7 B	€83.7 B	€8.6 B	€32.2 B	£18.2 B	$27.3 B	R$132.9 B	¥1.6 T	$1.2 T
		90% adherence to GDMT	€132.0 B	€76.4 B	€7.2 B	€27.8 B	£17.5 B	$24.1 B	R$116.1 B	¥1.3 T	$1.1 T
		% Change	**-9.4%**	**-8.7%**	**-16.2%**	**-13.5%**	**-3.5%**	**-11.7%**	**-12.6%**	**-17.8%**	**-7.7%**
Total Costs of non-KRT CKD & KRT[b]	25 Years	Current practice	€1.4 T	€883.1 B	€86.1 B	€296.9 B	£149.1 B	$232.0 B	R$1.6 T	¥79.4 T	$11.2 T
		90% adherence to GDMT	€1.3 T	€799.8 B	€74.3 B	€255.6 B	£136.7 B	$192.3 B	R$1.4 T	¥77.1 T	$10.7 T
		% Change	**-10.5%**	**-9.4%**	**-13.7%**	**-13.9%**	**-8.3%**	**-17.1%**	**-8.4%**	**-3.0%**	**-4.8%**
	10 Years	Current practice	€521.8 B	€323.8 B	€30.5 B	€101.2 B	£56.5 B	$84.6 B	R$601.3 B	¥29.3 T	$4.4 T
		90% adherence to GDMT	€501.0 B	€313.4 B	€26.8 B	€95.9 B	£55.3 B	$78.2 B	R$583.2 B	¥28.6 T	$4.4 T
		% Change	**-4.0%**	**-3.2%**	**-11.9%**	**-5.3%**	**-2.1%**	**-7.5%**	**-3.0%**	**-2.5%**	**-1.6%**

Outcome	Time Horizon	Scenario	EUR	Germany	Netherlands	Spain	UK	Australia	Brazil	China	US
Total Costs of non-KRT CKD & KRT Including Treatment Costs[c]	25 Years	Current practice	€1.4 T	€883.1 B	€86.1 B	€296.9 B	£149.1 B	$232.0 B	R$1.6 T	¥79.4 T	$11.2 T
		90% adherence to GDMT	€1.4 T	€848.3 B	€80.7 B	€274.7 B	£157.3 B	$200.9 B	R$1.8 T	¥81.0 T	$11.5 T
		% Change	**-3.7%**	**-3.9%**	**-6.3%**	**-7.5%**	**5.5%**	**-13.4%**	**13.9%**	**2.0%**	**2.6%**
	10 Years	Current practice	€521.8 B	€323.8 B	€30.5 B	€101.2 B	£56.5 B	$84.6 B	R$601.3 B	¥29.3 T	$4.4 T
		90% adherence to GDMT	€536.0 B	€330.0 B	€29.3 B	€102.5 B	£63.3 B	$81.4 B	R$708.4 B	¥30.1 T	$4.7 T
		% Change	**2.7%**	**1.9%**	**-3.9%**	**1.2%**	**12.0%**	**-3.7%**	**17.8%**	**2.7%**	**5.7%**
**Cumulative Environmental Burden**											
All CKD (incl. KRT) Freshwater Consumption (m^3)	25 Years	Current practice	2.5 B	969.5 M	210.5 M	732.9 M	576.2 M	283.4 M	10.6 B	18.9 B	87.7 B
		90% adherence to GDMT	2.3 B	875.9 M	209.5 M	697.8 M	560.2 M	261.9 M	10.1 B	18.0 B	88.5 B
		% Change	**-5.9%**	**-9.7%**	**-0.5%**	**-4.8%**	**-2.8%**	**-7.6%**	**-4.1%**	**-5.2%**	**0.8%**
	10 Years	Current practice	960.9 M	375.7 M	81.9 M	270.2 M	233.0 M	107.0 M	4.2 B	7.0 B	36.4 B
		90% adherence to GDMT	946.0 M	367.1 M	80.9 M	265.9 M	232.1 M	104.3 M	4.1 B	6.8 B	36.5 B
		% Change	**-1.6%**	**-2.3%**	**-1.2%**	**-1.6%**	**-0.4%**	**-2.5%**	**-1.4%**	**-1.8%**	**0.3%**
All CKD (incl. KRT) Fossil Fuel Depletion (kg oil eq)	25 Years	Current practice	192.5 B	71.1 B	14.8 B	53.3 B	53.4 B	76.4 B	176.7 B	1.7 T	1.3 T
		90% adherence to GDMT	187.6 B	67.8 B	14.8 B	52.1 B	52.9 B	73.2 B	171.5 B	1.7 T	1.3 T
		% Change	**-2.5%**	**-4.7%**	**0.4%**	**-2.1%**	**-1.0%**	**-4.3%**	**-3.0%**	**-3.2%**	**1.1%**
	10 Years	Current practice	76.0 B	28.5 B	5.7 B	19.9 B	21.8 B	29.0 B	70.0 B	639.4 B	530.9 B
		90% adherence to GDMT	75.5 B	28.2 B	5.7 B	19.8 B	21.8 B	28.5 B	69.3 B	631.6 B	532.7 B
		% Change	**-0.7%**	**-1.2%**	**-0.2%**	**-0.8%**	**-0.2%**	**-1.8%**	**-1.0%**	**-1.2%**	**0.3%**

Outcome	Time Horizon	Scenario	EUR	Germany	Netherlands	Spain	UK	Australia	Brazil	China	US
All CKD (incl. KRT) Carbon Footprint (kg CO_2 eq)	25 Years	Current practice	467.2 B	186.3 B	37.4 B	122.7 B	120.7 B	25.9 B	496.6 B	4.7 T	3.4 T
		90% adherence to GDMT	454.1 B	177.0 B	37.6 B	120.0 B	119.6 B	24.8 B	480.5 B	4.5 T	3.4 T
		% Change	**-2.8%**	**-5.0%**	**0.4%**	**-2.3%**	**-0.9%**	**-4.1%**	**-3.2%**	**-3.3%**	**-0.7%**
	10 Years	Current practice	184.5 B	74.7 B	14.5 B	46.0 B	49.3 B	9.8 B	196.3 B	1.7 T	1.4 T
		90% adherence to GDMT	183.1 B	73.8 B	14.5 B	45.6 B	49.3 B	9.7 B	194.2 B	1.7 T	1.4 T
		% Change	**-0.8%**	**-1.2%**	**-0.3%**	**-0.9%**	**-0.1%**	**-1.7%**	**-1.1%**	**-1.3%**	**-1.8%**
KRT only Freshwater Consumption (m^3)	25 Years	Current practice	486.4 M	275.3 M	17.8 M	112.4 M	80.9 M	50.2 M	1.7 B	2.7 B	6.9 B
		90% adherence to GDMT	310.9 M	168.5 M	14.5 M	67.9 M	59.9 M	26.7 M	1.0 B	1.5 B	4.8 B
		% Change	**-36.1%**	**-38.8%**	**-18.2%**	**-39.6%**	**-25.9%**	**-46.9%**	**-41.6%**	**-44.0%**	**-30.3%**
	10 Years	Current practice	156.6 M	87.2 M	7.5 M	34.0 M	28.0 M	17.6 M	461.4 M	665.0 M	2.5 B
		90% adherence to GDMT	141.2 M	78.9 M	6.1 M	29.3 M	26.9 M	15.4 M	395.1 M	535.7 M	2.3 B
		% Change	**-9.8%**	**-9.5%**	**-18.2%**	**-13.8%**	**-4.0%**	**-12.9%**	**-14.4%**	**-19.4%**	**-8.4%**
KRT only Fossil Fuel Depletion (kg oil eq)	25 Years	Current practice	21.9 B	12.0 B	649.4 M	5.0 B	4.2 B	8.5 B	23.3 B	171.3 B	95.7 B
		90% adherence to GDMT	14.4 B	7.5 B	553.2 M	3.2 B	3.2 B	4.8 B	13.7 B	96.6 B	66.9 B
		% Change	**-34.2%**	**-37.8%**	**-14.8%**	**-37.1%**	**-23.7%**	**-44.3%**	**-41.2%**	**-43.6%**	**-30.1%**
	10 Years	Current practice	7.1 B	3.8 B	261.1 M	1.5 B	1.5 B	3.0 B	6.5 B	41.8 B	34.6 B
		90% adherence to GDMT	6.4 B	3.4 B	221.0 M	1.3 B	1.4 B	2.6 B	5.6 B	33.7 B	31.7 B
		% Change	**-9.1%**	**-9.2%**	**-15.4%**	**-12.8%**	**-3.7%**	**-12.2%**	**-14.2%**	**-19.2%**	**-8.4%**

Outcome	Time Horizon	Scenario	EUR	Germany	Netherlands	Spain	UK	Australia	Brazil	China	US
KRT only Carbon Footprint (kg CO_2 eq)	25 Years	Current practice	56.3 B	33.0 B	1.8 B	12.0 B	9.5 B	2.8 B	68.5 B	485.0 B	231.2 B
		90% adherence to GDMT	36.8 B	20.5 B	1.5 B	7.5 B	7.3 B	1.6 B	40.2 B	273.3 B	161.6 B
		% Change	**-34.7%**	**-37.9%**	**-15.3%**	**-37.3%**	**-23.7%**	**-44.2%**	**-41.3%**	**-43.7%**	**-30.1%**
	10 Years	Current practice	18.1 B	10.5 B	714.9 M	3.7 B	3.3 B	991.2 M	19.0 B	118.2 B	83.8 B
		90% adherence to GDMT	16.5 B	9.5 B	601.9 M	3.2 B	3.2 B	870.8 M	16.3 B	95.5 B	76.7 B
		% Change	**-9.2%**	**-9.3%**	**-15.8%**	**-12.9%**	**-3.7%**	**-12.2%**	**-14.2%**	**-19.2%**	**-8.4%**

[a]Currency conversion for the UK from GBP (£) to euro (€) was performed prior to aggregation across European countries using the 2022 annual average from ECB.[26] Values used in conversions were as follows: £1.0 = €1.173.

[b]Does not include costs associated with CKD treatment or screening.

[c]Does not include costs associated with CKD screening.

Abbreviations: B = billion; CKD = chronic kidney disease; CV = cardiovascular; eq = equivalent; ECB = European Central Bank; EUR = Europe; GBP = pound sterling; GDMT = guideline-directed medical therapy; HHF = hospitalization from heart failure; incl. = including; KRT = kidney replacement therapy; M = million; K = thousand; MI = myocardial infarction; T = trillion; UK = United Kingdoms; US = United States.

Supplementary Table S17. CKD burden over 10 and 25 years following targeted screening every 3 years for high-risk population and a 75% adherence to GDMT compared to current practice.

Outcome	Time Horizon	Scenario	EUR	Germany	Netherlands	Spain	UK	Australia	Brazil	China	US
**Clinical Burden - Cumulative CKD and KRT prevalence**											
CKD stage 1-2	25 Years	Current practice	396.6 M	151.4 M	32.4 M	96.1 M	116.6 M	54.2 M	394.4 M	3.5 B	838.8 M
		Targeted screening every 3 years 75% adherence to GDMT	413.7 M	157.8 M	34.0 M	101.0 M	120.9 M	57.0 M	409.3 M	3.6 B	883.5 M
		% Change	**4.3%**	**4.2%**	**4.7%**	**5.1%**	**3.7%**	**5.0%**	**3.8%**	**3.6%**	**5.3%**
	10 Years	Current practice	178.6 M	70.6 M	14.2 M	42.5 M	51.2 M	21.3 M	159.6 M	1.4 B	360.9 M
		Targeted screening every 3 years 75% adherence to GDMT	181.7 M	71.9 M	14.5 M	43.3 M	52.0 M	21.6 M	161.9 M	1.4 B	368.8 M
		% Change	**1.7%**	**1.8%**	**1.7%**	**1.9%**	**1.5%**	**1.8%**	**1.4%**	**1.3%**	**2.2%**
CKD stage 3-5 (incl. KRT)	25 Years	Current practice	391.5 M	151.7 M	35.1 M	93.4 M	111.3 M	31.2 M	326.1 M	1.8 B	650.8 M
		Targeted screening every 3 years 75% adherence to GDMT	394.0 M	152.8 M	35.5 M	94.1 M	111.6 M	30.1 M	334.7 M	1.8 B	650.8 M
		% Change	**0.6%**	**0.8%**	**1.1%**	**0.7%**	**0.2%**	**-3.6%**	**2.6%**	**-1.3%**	**0.3%**
	10 Years	Current practice	145.3 M	57.0 M	12.7 M	31.8 M	43.9 M	11.6 M	136.4 M	695.4 M	265.9 M
		Targeted screening every 3 years 75% adherence to GDMT	145.3 M	56.9 M	12.7 M	31.8 M	43.9 M	11.4 M	137.5 M	689.4 M	263.9 M
		% Change	**0.0%**	**-0.1%**	**0.4%**	**-0.1%**	**0.1%**	**-1.8%**	**0.8%**	**-0.9%**	**-0.7%**

Outcome	Time Horizon	Scenario	EUR	Germany	Netherlands	Spain	UK	Australia	Brazil	China	US
Dialysis	25 Years	Current practice	8.5 M	4.9 M	197.9 K	1.8 M	1.6 M	705.4 K	10.1 M	59.5 M	26.9 M
		Targeted screening every 3 years 75% adherence to GDMT	4.8 M	2.7 M	145.3 K	937.2 K	1.0 M	318.7 K	5.3 M	29.4 M	17.0 M
		% Change	**-43.3%**	**-45.1%**	**-26.6%**	**-48.4%**	**-34.2%**	**-54.8%**	**-47.2%**	**-50.6%**	**-36.8%**
	10 Years	Current practice	2.7 M	1.5 M	86.8 K	538.4 K	544.0 K	248.7 K	2.8 M	14.3 M	9.8 M
		Targeted screening every 3 years 75% adherence to GDMT	2.4 M	1.4 M	66.7 K	451.9 K	513.8 K	212.0 K	2.3 M	11.2 M	8.9 M
		% Change	**-11.3%**	**-10.9%**	**-23.2%**	**-16.1%**	**-5.6%**	**-14.8%**	**-16.3%**	**-21.8%**	**-10.0%**
Transplant	25 Years	Current practice	5.8 M	2.0 M	529.7 K	1.5 M	1.8 M	539.1 K	3.3 M	8.6 M	12.1 M
		Targeted screening every 3 years 75% adherence to GDMT	5.8 M	2.0 M	528.5 K	1.5 M	1.8 M	537.7 K	3.3 M	8.6 M	12.1 M
		% Change	**0.0%**	**0.1%**	**-0.2%**	**0.4%**	**-0.3%**	**-0.3%**	**0.7%**	**0.2%**	**0.2%**
	10 Years	Current practice	1.9 M	649.0 K	172.5 K	513.6 K	576.1 K	178.4 K	1.0 M	2.8 M	4.0 M
		Targeted screening every 3 years 75% adherence to GDMT	1.9 M	649.2 K	172.4 K	512.2 K	574.7 K	178.3 K	1.0 M	2.8 M	4.0 M
		% Change	**-0.2%**	**0.0%**	**-0.1%**	**-0.3%**	**-0.2%**	**0.0%**	**0.1%**	**0.0%**	**-0.1%**

Outcome	Time Horizon	Scenario	EUR	Germany	Netherlands	Spain	UK	Australia	Brazil	China	US
**Clinical Burden – Cumulative number of clinical events**											
CV events (MI, stroke, HHF)	25 Years	Current practice	12.2 M	5.1 M	1.1 M	2.3 M	3.7 M	2.1 M	11.6 M	59.6 M	41.2 M
		Targeted screening every 3 years 75% adherence to GDMT	7.0 M	2.9 M	621.8 K	1.4 M	2.1 M	1.1 M	6.4 M	37.9 M	22.6 M
		% Change	**-42.7%**	**-42.1%**	**-44.0%**	**-41.6%**	**-43.9%**	**-47.2%**	**-45.1%**	**-36.5%**	**-45.1%**
	10 Years	Current practice	4.1 M	1.8 M	327.8 K	702.5 K	1.3 M	532.0 K	3.3 M	17.5 M	10.9 M
		Targeted screening every 3 years 75% adherence to GDMT	2.6 M	1.1 M	209.0 K	460.3 K	793.7 K	326.1 K	2.1 M	12.4 M	6.5 M
		% Change	**-36.8%**	**-37.7%**	**-36.2%**	**-34.5%**	**-36.8%**	**-38.7%**	**-38.2%**	**-29.3%**	**-40.6%**
Death Events	25 Years	Current practice	27.6 M	12.2 M	2.0 M	5.2 M	8.2 M	1.8 M	18.8 M	112.9 M	38.7 M
		Targeted screening every 3 years 75% adherence to GDMT	26.3 M	11.7 M	1.9 M	4.8 M	8.0 M	1.7 M	17.2 M	104.4 M	35.3 M
		% Change	**-4.5%**	**-3.8%**	**-6.3%**	**-7.8%**	**-3.1%**	**-7.2%**	**-8.3%**	**-7.6%**	**-8.8%**
	10 Years	Current practice	10.2 M	5.0 M	612.9 K	1.6 M	3.0 M	538.2 K	6.0 M	34.3 M	12.6 M
		Targeted screening every 3 years 75% adherence to GDMT	9.7 M	4.8 M	560.9 K	1.5 M	2.9 M	501.6 K	5.4 M	32.2 M	11.4 M
		% Change	**-5.0%**	**-4.1%**	**-8.5%**	**-8.2%**	**-4.0%**	**-6.8%**	**-9.1%**	**-6.3%**	**-9.1%**

Outcome	Time Horizon	Scenario	EUR	Germany	Netherlands	Spain	UK	Australia	Brazil	China	US
**Cumulative Economic Burden[a]**											
Non-KRT CKD Costs[b]	25 Years	Current practice	€989.2 B	€620.6 B	€65.2 B	€190.7 B	£96.2 B	$154.1 B	R$1.1 T	¥73.0 T	$7.8 T
		Targeted screening every 3 years 75% adherence to GDMT	€1.0 T	€646.4 B	€53.7 B	€189.9 B	£97.4 B	$142.1 B	R$1.2 T	¥74.4 T	$8.3 T
		% Change	**1.5%**	**4.2%**	**-17.5%**	**-0.4%**	**1.3%**	**-7.8%**	**6.5%**	**1.9%**	**6.0%**
	10 Years	Current practice	€376.0 B	€240.1 B	€21.9 B	€69.1 B	£38.3 B	$57.2 B	R$468.4 B	¥27.7 T	$3.2 T
		Targeted screening every 3 years 75% adherence to GDMT	€379.3 B	€244.6 B	€19.3 B	€69.6 B	£39.1 B	$54.7 B	R$484.9 B	¥28.2 T	$3.3 T
		% Change	**0.9%**	**1.9%**	**-11.7%**	**0.7%**	**2.0%**	**-4.5%**	**3.5%**	**1.8%**	**3.9%**
CKD Treatment Costs	25 Years	Incremental Treatment Costs	€204.1 B	€107.7 B	€11.8 B	€37.1 B	£40.4 B	$22.5 B	R$731.9 B	¥10.8 T	$2.2 T
	10 Years	Incremental Treatment Costs	€77.6 B	€41.0 B	€4.7 B	€13.4 B	£15.9 B	$8.5 B	R$236.8 B	¥4.0 T	$901.1 B
KRT costs[b]	25 Years	Current practice	€451.7 B	€262.5 B	€21.0 B	€106.2 B	£52.9 B	$78.0 B	R$469.2 B	¥6.5 T	$3.4 T
		Targeted screening every 3 years 75% adherence to GDMT	€281.6 B	€158.0 B	€17.0 B	€60.5 B	£39.3 B	$41.8 B	R$280.5 B	¥3.5 T	$2.4 T
		% Change	**-37.7%**	**-39.8%**	**-19.0%**	**-43.1%**	**-25.7%**	**-46.4%**	**-40.2%**	**-46.4%**	**-29.6%**
	10 Years	Current practice	€145.7 B	€83.7 B	€8.6 B	€32.2 B	£18.2 B	$27.3 B	R$132.9 B	¥1.6 T	$1.2 T
		Targeted screening every 3 years 75% adherence to GDMT	€130.8 B	€75.7 B	€7.1 B	€27.6 B	£17.4 B	$23.9 B	R$115.0 B	¥1.3 T	$1.1 T
		% Change	**-10.3%**	**-9.6%**	**-17.7%**	**-14.1%**	**-4.3%**	**-12.5%**	**-13.5%**	**-19.4%**	**-8.2%**

Outcome	Time Horizon	Scenario	EUR	Germany	Netherlands	Spain	UK	Australia	Brazil	China	US
Total Costs of non-KRT CKD & KRT[b]	25 Years	Current practice	€1.4 T	€883.1 B	€86.1 B	€296.9 B	£149.1 B	$232.0 B	R$1.6 T	¥79.4 T	$11.2 T
		Targeted screening every 3 years 75% adherence to GDMT	€1.3 T	€804.4 B	€70.7 B	€250.3 B	£136.7 B	$183.9 B	R$1.5 T	¥77.8 T	$10.7 T
		% Change	**-10.8%**	**-8.9%**	**-17.9%**	**-15.7%**	**-8.3%**	**-20.7%**	**-7.5%**	**-2.0%**	**-4.8%**
	10 Years	Current practice	€521.8 B	€323.8 B	€30.5 B	€101.2 B	£56.5 B	$84.6 B	R$601.3 B	¥29.3 T	$4.4 T
		Targeted screening every 3 years 75% adherence to GDMT	€510.1 B	€320.2 B	€26.4 B	€97.2 B	£56.5 B	$78.6 B	R$599.8 B	¥29.5 T	$4.5 T
		% Change	**-2.2%**	**-1.1%**	**-13.4%**	**-4.0%**	**-0.1%**	**-7.1%**	**-0.2%**	**0.6%**	**0.6%**
Total Costs of non-KRT CKD & KRT Including Treatment Costs[c]	25 Years	Current practice	€1.4 T	€883.1 B	€86.1 B	€296.9 B	£149.1 B	$232.0 B	R$1.6 T	¥79.4 T	$11.2 T
		Targeted screening every 3 years 75% adherence to GDMT	€1.5 T	€912.1 B	€82.5 B	€287.5 B	£177.1 B	$206.5 B	R$2.2 T	¥88.6 T	$12.8 T
		% Change	**3.4%**	**3.3%**	**-4.2%**	**-3.2%**	**18.8%**	**-11.0%**	**39.1%**	**11.6%**	**14.6%**
	10 Years	Current practice	€521.8 B	€323.8 B	€30.5 B	€101.2 B	£56.5 B	$84.6 B	R$601.3 B	¥29.3 T	$4.4 T
		Targeted screening every 3 years 75% adherence to GDMT	€587.7 B	€361.2 B	€31.1 B	€110.6 B	£72.4 B	$87.1 B	R$836.6 B	¥33.5 T	$5.4 T
		% Change	**12.6%**	**11.6%**	**2.0%**	**9.2%**	**28.1%**	**3.0%**	**39.1%**	**14.2%**	**20.9%**

Outcome	Time Horizon	Scenario	EUR	Germany	Netherlands	Spain	UK	Australia	Brazil	China	US
**Cumulative Environmental Burden**											
All CKD (incl. KRT) Freshwater Consumption (m^3)	25 Years	Current practice	2.5 B	969.5 M	210.5 M	732.9 M	576.2 M	283.4 M	10.6 B	18.9 B	87.7 B
		Targeted screening every 3 years 75% adherence to GDMT	2.4 B	903.2 M	218.8 M	729.1 M	582.8 M	273.6 M	10.6 B	18.8 B	93.6 B
		% Change	**-2.2%**	**-6.8%**	**3.9%**	**-0.5%**	**1.1%**	**-3.4%**	**0.5%**	**-0.7%**	**6.7%**
	10 Years	Current practice	960.9 M	375.7 M	81.9 M	270.2 M	233.0 M	107.0 M	4.2 B	7.0 B	36.4 B
		Targeted screening every 3 years 75% adherence to GDMT	984.6 M	381.0 M	84.2 M	277.6 M	241.8 M	109.1 M	4.3 B	7.2 B	38.4 B
		% Change	**2.5%**	**1.4%**	**2.8%**	**2.8%**	**3.8%**	**2.0%**	**2.9%**	**2.7%**	**5.4%**
All CKD (incl. KRT) Fossil Fuel Depletion (kg oil eq)	25 Years	Current practice	192.5 B	71.1 B	14.8 B	53.3 B	53.4 B	76.4 B	176.7 B	1.7 T	1.3 T
		Targeted screening every 3 years 75% adherence to GDMT	196.4 B	70.8 B	15.5 B	54.8 B	55.3 B	76.7 B	180.5 B	1.7 T	1.4 T
		% Change	**2.1%**	**-0.4%**	**5.3%**	**2.8%**	**3.6%**	**0.4%**	**2.1%**	**1.9%**	**6.9%**
	10 Years	Current practice	76.0 B	28.5 B	5.7 B	19.9 B	21.8 B	29.0 B	70.0 B	639.4 B	530.9 B
		Targeted screening every 3 years 75% adherence to GDMT	78.9 B	29.5 B	6.0 B	20.7 B	22.8 B	29.9 B	72.4 B	661.8 B	560.3 B
		% Change	**3.8%**	**3.3%**	**4.1%**	**3.8%**	**4.3%**	**3.1%**	**3.5%**	**3.5%**	**5.5%**

Outcome	Time Horizon	Scenario	EUR	Germany	Netherlands	Spain	UK	Australia	Brazil	China	US
All CKD (incl. KRT) Carbon Footprint (kg CO_2 eq)	25 Years	Current practice	467.2 B	186.3 B	37.4 B	122.7 B	120.7 B	25.9 B	496.6 B	4.7 T	3.4 T
		Targeted screening every 3 years 75% adherence to GDMT	475.5 B	184.9 B	39.4 B	126.0 B	125.2 B	26.0 B	505.6 B	4.8 T	3.6 T
		% Change	**1.8%**	**-0.8%**	**5.2%**	**2.7%**	**3.7%**	**0.6%**	**1.8%**	**1.7%**	**3.4%**
	10 Years	Current practice	184.5 B	74.7 B	14.5 B	46.0 B	49.3 B	9.8 B	196.3 B	1.7 T	1.4 T
		Targeted screening every 3 years 75% adherence to GDMT	191.5 B	77.2 B	15.1 B	47.7 B	51.5 B	10.1 B	202.9 B	1.8 T	1.5 T
		% Change	**3.7%**	**3.3%**	**4.0%**	**3.8%**	**4.4%**	**3.2%**	**3.4%**	**3.5%**	**2.4%**
KRT only Freshwater Consumption (m^3)	25 Years	Current practice	486.4 M	275.3 M	17.8 M	112.4 M	80.9 M	50.2 M	1.7 B	2.7 B	6.9 B
		Targeted screening every 3 years 75% adherence to GDMT	289.6 M	156.3 M	13.9 M	63.1 M	56.2 M	24.7 M	0.9 B	1.4 B	4.6 B
		% Change	**-40.5%**	**-43.2%**	**-21.7%**	**-43.8%**	**-30.5%**	**-50.8%**	**-44.7%**	**-49.5%**	**-33.0%**
	10 Years	Current practice	156.6 M	87.2 M	7.5 M	34.0 M	28.0 M	17.6 M	461.4 M	665.0 M	2.5 B
		Targeted screening every 3 years 75% adherence to GDMT	139.8 M	78.1 M	6.0 M	29.1 M	26.6 M	15.2 M	390.6 M	524.3 M	2.3 B
		% Change	**-10.8%**	**-10.5%**	**-19.8%**	**-14.3%**	**-5.0%**	**-13.7%**	**-15.3%**	**-21.2%**	**-9.0%**

Outcome	Time Horizon	Scenario	EUR	Germany	Netherlands	Spain	UK	Australia	Brazil	China	US
KRT only Fossil Fuel Depletion (kg oil eq)	25 Years	Current practice	21.9 B	12.0 B	649.4 M	5.0 B	4.2 B	8.5 B	23.3 B	171.3 B	95.7 B
		Targeted screening every 3 years 75% adherence to GDMT	13.5 B	6.9 B	535.0 M	3.0 B	3.1 B	4.4 B	13.0 B	87.3 B	64.3 B
		% Change	**-38.4%**	**-42.1%**	**-17.6%**	**-41.0%**	**-27.8%**	**-48.0%**	**-44.3%**	**-49.0%**	**-32.8%**
	10 Years	Current practice	7.1 B	3.8 B	261.1 M	1.5 B	1.5 B	3.0 B	6.5 B	41.8 B	34.6 B
		Targeted screening every 3 years 75% adherence to GDMT	6.4 B	3.4 B	217.5 M	1.3 B	1.4 B	2.6 B	5.5 B	33.0 B	31.5 B
		% Change	**-10.0%**	**-10.2%**	**-16.7%**	**-13.3%**	**-4.6%**	**-13.0%**	**-15.1%**	**-20.9%**	**-9.0%**
KRT only Carbon Footprint (kg CO_2 eq)	25 Years	Current practice	56.3 B	33.0 B	1.8 B	12.0 B	9.5 B	2.8 B	68.5 B	485.0 B	231.2 B
		Targeted screening every 3 years 75% adherence to GDMT	34.5 B	19.1 B	1.4 B	7.1 B	6.9 B	1.5 B	38.1 B	247.0 B	155.4 B
		% Change	**-38.9%**	**-42.3%**	**-18.2%**	**-41.2%**	**-27.8%**	**-47.9%**	**-44.4%**	**-49.1%**	**-32.8%**
	10 Years	Current practice	18.1 B	10.5 B	714.9 M	3.7 B	3.3 B	991.2 M	19.0 B	118.2 B	83.8 B
		Targeted screening every 3 years 75% adherence to GDMT	16.3 B	9.4 B	592.0 M	3.2 B	3.1 B	862.7 M	16.1 B	93.5 B	76.2 B
		% Change	**-10.1%**	**-10.2%**	**-17.2%**	**-13.4%**	**-4.6%**	**-13.0%**	**-15.2%**	**-20.9%**	**-9.0%**

[a]Currency conversion for the UK from GBP (£) to euro (€) was performed prior to aggregation across European countries using the 2022 annual average from ECB.[26] Values used in conversions were as follows: £1.0 = €1.173.

[b]Does not include costs associated with CKD treatment or screening.

[c]Does not include costs associated with CKD screening.

Abbreviations: B = billion; CKD = chronic kidney disease; CV = cardiovascular; eq = equivalent; ECB = European Central Bank; EUR = Europe; GBP = pound sterling; GDMT = guideline-directed medical therapy; HHF = hospitalization from heart failure; incl. = including; K = thousand; KRT = kidney replacement therapy; M = million; MI = myocardial infarction; T = trillion; UK = United Kingdoms; US = United States.

Supplementary Table S18. CKD burden over 10 and 25 years following targeted screening every 5 years for high-risk population and a 75% adherence to GDMT compared to current practice.

Outcome	Time Horizon	Scenario	EUR	Germany	Netherlands	Spain	UK	Australia	Brazil	China	US
**Clinical Burden - Cumulative CKD and KRT prevalence**											
CKD stage 1-2	25 Years	Current practice	396.6 M	151.4 M	32.4 M	96.1 M	116.6 M	54.2 M	394.4 M	3.5 B	838.8 M
		Targeted screening every 5 years 75% adherence to GDMT	410.9 M	156.7 M	33.7 M	100.3 M	120.3 M	56.6 M	407.0 M	3.6 B	875.4 M
		% Change	**3.6%**	**3.5%**	**4.0%**	**4.3%**	**3.1%**	**4.3%**	**3.2%**	**3.0%**	**4.4%**
	10 Years	Current practice	178.6 M	70.6 M	14.2 M	42.5 M	51.2 M	21.3 M	159.6 M	1.4 B	360.9 M
		Targeted screening every 5 years 75% adherence to GDMT	180.4 M	71.3 M	14.4 M	43.0 M	51.7 M	21.5 M	161.0 M	1.4 B	365.3 M
		% Change	**1.0%**	**1.0%**	**1.1%**	**1.1%**	**0.9%**	**1.1%**	**0.9%**	**0.8%**	**1.2%**
CKD stage 3-5 (incl. KRT)	25 Years	Current practice	391.5 M	151.7 M	35.1 M	93.4 M	111.3 M	31.2 M	326.1 M	1.8 B	649.0 M
		Targeted screening every 5 years 75% adherence to GDMT	395.0 M	153.3 M	35.5 M	94.3 M	111.8 M	30.3 M	335.1 M	1.8 B	654.2 M
		% Change	**0.9%**	**1.1%**	**1.3%**	**1.0%**	**0.4%**	**-2.8%**	**2.7%**	**-0.8%**	**0.8%**
	10 Years	Current practice	145.3 M	57.0 M	12.7 M	31.8 M	43.9 M	11.6 M	136.4 M	695.4 M	265.9 M
		Targeted screening every 5 years 75% adherence to GDMT	146.0 M	57.3 M	12.8 M	31.9 M	44.1 M	11.5 M	137.8 M	693.8 M	266.2 M
		% Change	**0.5%**	**0.5%**	**0.7%**	**0.4%**	**0.4%**	**-0.9%**	**1.0%**	**-0.2%**	**0.1%**

Outcome	Time Horizon	Scenario	EUR	Germany	Netherlands	Spain	UK	Australia	Brazil	China	US
Dialysis	25 Years	Current practice	8.5 M	4.9 M	197.9 K	1.8 M	1.6 M	705.4 K	10.1 M	59.5 M	26.9 M
		Targeted screening every 5 years 75% adherence to GDMT	5.0 M	2.8 M	152.0 K	1.0 M	1.1 M	334.7 K	5.6 M	31.7 M	17.5 M
		% Change	**-40.4%**	**-42.1%**	**-23.2%**	**-45.4%**	**-31.6%**	**-52.6%**	**-44.5%**	**-46.8%**	**-34.8%**
	10 Years	Current practice	2.7 M	1.5 M	86.8 K	538.4 K	544.0 K	248.7 K	2.8 M	14.3 M	9.8 M
		Targeted screening every 5 years 75% adherence to GDMT	2.5 M	1.4 M	69.2 K	458.7 K	523.9 K	215.6 K	2.4 M	11.7 M	9.0 M
		% Change	**-9.7%**	**-9.4%**	**-20.4%**	**-14.8%**	**-3.7%**	**-13.3%**	**-14.0%**	**-18.3%**	**-8.6%**
Transplant	25 Years	Current practice	5.8 M	2.0 M	529.7 K	1.5 M	1.8 M	539.1 K	3.3 M	8.6 M	12.1 M
		Targeted screening every 5 years 75% adherence to GDMT	5.8 M	2.0 M	527.2 K	1.5 M	1.8 M	538.6 K	3.3 M	8.6 M	12.1 M
		% Change	**-0.1%**	**0.1%**	**-0.5%**	**0.7%**	**-0.7%**	**-0.1%**	**0.7%**	**0.2%**	**-0.1%**
	10 Years	Current practice	1.9 M	649.0 K	172.5 K	513.6 K	576.1 K	178.4 K	1.0 M	2.8 M	4.0 M
		Targeted screening every 5 years 75% adherence to GDMT	1.9 M	649.2 K	172.3 K	513.1 K	574.3 K	178.3 K	1.0 M	2.8 M	4.0 M
		% Change	**-0.1%**	**0.0%**	**-0.1%**	**-0.1%**	**-0.3%**	**-0.1%**	**0.0%**	**0.0%**	**-0.2%**

Outcome	Time Horizon	Scenario	EUR	Germany	Netherlands	Spain	UK	Australia	Brazil	China	US
**Clinical Burden – Cumulative number of clinical events**											
CV events (MI, stroke, HHF)	25 Years	Current practice	12.2 M	5.1 M	1.1 M	2.3 M	3.7 M	2.1 M	11.6 M	59.6 M	41.2 M
		Targeted screening every 5 years 75% adherence to GDMT	7.4 M	3.1 M	651.3 K	1.4 M	2.2 M	1.2 M	6.6 M	39.4 M	23.7 M
		% Change	**-39.6%**	**-38.6%**	**-41.3%**	**-38.9%**	**-41.0%**	**-44.7%**	**-42.8%**	**-33.9%**	**-42.5%**
	10 Years	Current practice	4.1 M	1.8 M	327.8 K	702.5 K	1.3 M	532.0 K	3.3 M	17.5 M	10.9 M
		Targeted screening every 5 years 75% adherence to GDMT	2.9 M	1.3 M	228.1 K	504.4 K	875.4 K	361.5 K	2.3 M	13.5 M	7.3 M
		% Change	**-29.9%**	**-30.1%**	**-30.4%**	**-28.2%**	**-30.3%**	**-32.0%**	**-32.2%**	**-23.1%**	**-33.1%**
Death Events	25 Years	Current practice	27.6 M	12.2 M	2.0 M	5.2 M	8.2 M	1.8 M	18.8 M	112.9 M	38.7 M
		Targeted screening every 5 years 75% adherence to GDMT	26.4 M	11.8 M	1.9 M	4.8 M	8.0 M	1.7 M	17.3 M	104.9 M	35.5 M
		% Change	**-4.3%**	**-3.6%**	**-6.0%**	**-7.4%**	**-2.9%**	**-6.8%**	**-7.9%**	**-7.1%**	**-8.2%**
	10 Years	Current practice	10.2 M	5.0 M	612.9 K	1.6 M	3.0 M	538.2 K	6.0 M	34.3 M	12.6 M
		Targeted screening every 5 years 75% adherence to GDMT	9.8 M	4.8 M	568.1 K	1.5 M	2.9 M	508.5 K	5.5 M	32.6 M	11.6 M
		% Change	**-4.2%**	**-3.4%**	**-7.3%**	**-7.0%**	**-3.4%**	**-5.5%**	**-7.8%**	**-5.2%**	**-7.4%**

Outcome	Time Horizon	Scenario	EUR	Germany	Netherlands	Spain	UK	Australia	Brazil	China	US
**Cumulative Economic Burden[a]**											
Non-KRT CKD Costs[b]	25 Years	Current practice	€989.2 B	€620.6 B	€65.2 B	€190.7 B	£96.2 B	$154.1 B	R$1.1 T	¥73.0 T	$7.8 T
		Targeted screening every 5 years 75% adherence to GDMT	€1.0 T	€643.6 B	€54.4 B	€190.3 B	£97.0 B	$143.8 B	R$1.2 T	¥74.2 T	$8.3 T
		% Change	**1.3%**	**3.7%**	**-16.5%**	**-0.2%**	**0.9%**	**-6.7%**	**5.9%**	**1.7%**	**5.8%**
	10 Years	Current practice	€376.0 B	€240.1 B	€21.9 B	€69.1 B	£38.3 B	$57.2 B	R$468.4 B	¥27.7 T	$3.2 T
		Targeted screening every 5 years 75% adherence to GDMT	€377.3 B	€242.7 B	€19.7 B	€69.5 B	£38.7 B	$55.1 B	R$479.9 B	¥28.0 T	$3.3 T
		% Change	**0.3%**	**1.1%**	**-10.1%**	**0.6%**	**1.1%**	**-3.8%**	**2.5%**	**1.0%**	**3.2%**
CKD Treatment Costs	25 Years	Incremental Treatment Costs	€188.5 B	€98.8 B	€11.1 B	€34.6 B	£37.5 B	$20.9 B	R$673.6 B	¥10.0 T	$2.0 T
	10 Years	Incremental Treatment Costs	€64.5 B	€33.4 B	€4.1 B	€11.3 B	£13.4 B	$7.0 B	R$195.2 B	¥3.3 T	$750.5 B
KRT costs[b]	25 Years	Current practice	€451.7 B	€262.5 B	€21.0 B	€106.2 B	£52.9 B	$78.0 B	R$469.2 B	¥6.5 T	$3.4 T
		Targeted screening every 5 years 75% adherence to GDMT	€292.9 B	€164.8 B	€17.5 B	€63.3 B	£40.3 B	$43.4 B	R$291.3 B	¥3.7 T	$2.4 T
		% Change	**-35.2%**	**-37.2%**	**-16.6%**	**-40.4%**	**-23.8%**	**-44.4%**	**-37.9%**	**-42.8%**	**-28.1%**
	10 Years	Current practice	€145.7 B	€83.7 B	€8.6 B	€32.2 B	£18.2 B	$27.3 B	R$132.9 B	¥1.6 T	$1.2 T
		Targeted screening every 5 years 75% adherence to GDMT	€132.8 B	€76.8 B	€7.3 B	€28.0 B	£17.6 B	$24.3 B	R$117.5 B	¥1.3 T	$1.1 T
		% Change	**-8.9%**	**-8.2%**	**-15.5%**	**-12.9%**	**-2.9%**	**-11.2%**	**-11.6%**	**-16.2%**	**-7.1%**

Outcome	Time Horizon	Scenario	EUR	Germany	Netherlands	Spain	UK	Australia	Brazil	China	US
Total Costs of non-KRT CKD & KRT[b]	25 Years	Current practice	€1.4 T	€883.1 B	€86.1 B	€296.9 B	£149.1 B	$232.0 B	R$1.6 T	¥79.4 T	$11.2 T
		Targeted screening every 5 years 75% adherence to GDMT	€1.3 T	€808.5 B	€71.9 B	€253.6 B	£137.3 B	$187.2 B	R$1.5 T	¥77.9 T	$10.7 T
		% Change	**-10.1%**	**-8.5%**	**-16.5%**	**-14.6%**	**-7.9%**	**-19.3%**	**-7.2%**	**-1.9%**	**-4.4%**
	10 Years	Current practice	€521.8 B	€323.8 B	€30.5 B	€101.2 B	£56.5 B	$84.6 B	R$601.3 B	¥29.3 T	$4.4 T
		Targeted screening every 5 years 75% adherence to GDMT	€510.0 B	€319.5 B	€26.9 B	€97.5 B	£56.4 B	$79.4 B	R$597.4 B	¥29.4 T	$4.4 T
		% Change	**-2.3%**	**-1.3%**	**-11.6%**	**-3.7%**	**-0.2%**	**-6.2%**	**-0.7%**	**0.1%**	**0.4%**
Total Costs of non-KRT CKD & KRT Including Treatment Costs[c]	25 Years	Current practice	€1.4 T	€883.1 B	€86.1 B	€296.9 B	£149.1 B	$232.0 B	R$1.6 T	¥79.4 T	$11.2 T
		Targeted screening every 5 years 75% adherence to GDMT	€1.5 T	€907.3 B	€83.0 B	€288.2 B	£174.8 B	$208.1 B	R$2.1 T	¥87.9 T	$12.7 T
		% Change	**3.0%**	**2.7%**	**-3.6%**	**-2.9%**	**17.3%**	**-10.3%**	**35.7%**	**10.6%**	**13.5%**
	10 Years	Current practice	€521.8 B	€323.8 B	€30.5 B	€101.2 B	£56.5 B	$84.6 B	R$601.3 B	¥29.3 T	$4.4 T
		Targeted screening every 5 years 75% adherence to GDMT	€574.5 B	€352.9 B	€31.0 B	€108.8 B	£69.8 B	$86.4 B	R$792.6 B	¥32.6 T	$5.2 T
		% Change	**10.1%**	**9.0%**	**1.7%**	**7.5%**	**23.5%**	**2.1%**	**31.8%**	**11.3%**	**17.4%**

Outcome	Time Horizon	Scenario	EUR	Germany	Netherlands	Spain	UK	Australia	Brazil	China	US
**Cumulative Environmental Burden**											
All CKD (incl. KRT) Freshwater Consumption (m^3)	25 Years	Current practice	2.5 B	969.5 M	210.5 M	732.9 M	576.2 M	283.4 M	10.6 B	18.9 B	87.7 B
		Targeted screening every 5 years 75% adherence to GDMT	2.4 B	903.7 M	217.6 M	726.4 M	580.1 M	272.8 M	10.6 B	18.8 B	92.7 B
		% Change	**-2.5%**	**-6.8%**	**3.4%**	**-0.9%**	**0.7%**	**-3.7%**	**0.1%**	**-0.9%**	**5.7%**
	10 Years	Current practice	960.9 M	375.7 M	81.9 M	270.2 M	233.0 M	107.0 M	4.2 B	7.0 B	36.4 B
		Targeted screening every 5 years 75% adherence to GDMT	973.8 M	377.2 M	83.3 M	274.3 M	239.0 M	107.9 M	4.2 B	7.1 B	37.8 B
		% Change	**1.3%**	**0.4%**	**1.7%**	**1.5%**	**2.5%**	**0.9%**	**1.8%**	**1.6%**	**3.8%**
All CKD (incl. KRT) Fossil Fuel Depletion (kg oil eq)	25 Years	Current practice	192.5 B	71.1 B	14.8 B	53.3 B	53.4 B	76.4 B	176.7 B	1.7 T	1.3 T
		Targeted screening every 5 years 75% adherence to GDMT	195.3 B	70.5 B	15.4 B	54.4 B	55.0 B	76.4 B	179.5 B	1.7 T	1.4 T
		% Change	**1.5%**	**-0.8%**	**4.5%**	**2.2%**	**2.9%**	**-0.1%**	**1.6%**	**1.5%**	**5.9%**
	10 Years	Current practice	76.0 B	28.5 B	5.7 B	19.9 B	21.8 B	29.0 B	70.0 B	639.4 B	530.9 B
		Targeted screening every 5 years 75% adherence to GDMT	77.8 B	29.1 B	5.9 B	20.4 B	22.5 B	29.5 B	71.5 B	653.8 B	551.5 B
		% Change	**2.4%**	**1.9%**	**2.8%**	**2.4%**	**2.9%**	**1.8%**	**2.2%**	**2.3%**	**3.9%**

Outcome	Time Horizon	Scenario	EUR	Germany	Netherlands	Spain	UK	Australia	Brazil	China	US
All CKD (incl. KRT) Carbon Footprint (kg CO_2 eq)	25 Years	Current practice	467.2 B	186.3 B	37.4 B	122.7 B	120.7 B	25.9 B	496.6 B	4.7 T	3.4 T
		Targeted screening every 5 years 75% adherence to GDMT	472.8 B	184.0 B	39.1 B	125.3 B	124.3 B	25.9 B	502.8 B	4.7 T	3.5 T
		% Change	**1.2%**	**-1.2%**	**4.5%**	**2.1%**	**3.0%**	**0.1%**	**1.3%**	**1.3%**	**2.9%**
	10 Years	Current practice	184.5 B	74.7 B	14.5 B	46.0 B	49.3 B	9.8 B	196.3 B	1.7 T	1.4 T
		Targeted screening every 5 years 75% adherence to GDMT	188.9 B	76.1 B	14.9 B	47.1 B	50.8 B	10.0 B	200.5 B	1.8 T	1.4 T
		% Change	**2.4%**	**1.9%**	**2.7%**	**2.4%**	**3.0%**	**1.9%**	**2.2%**	**2.2%**	**1.3%**
KRT only Freshwater Consumption (m^3)	25 Years	Current practice	486.4 M	275.3 M	17.8 M	112.4 M	80.9 M	50.2 M	1.7 B	2.7 B	6.9 B
		Targeted screening every 5 years 75% adherence to GDMT	302.7 M	164.0 M	14.4 M	66.2 M	58.1 M	25.8 M	1.0 B	1.5 B	4.7 B
		% Change	**-37.8%**	**-40.4%**	**-19.0%**	**-41.1%**	**-28.2%**	**-48.7%**	**-42.2%**	**-45.7%**	**-31.3%**
	10 Years	Current practice	156.6 M	87.2 M	7.5 M	34.0 M	28.0 M	17.6 M	461.4 M	665.0 M	2.5 B
		Targeted screening every 5 years 75% adherence to GDMT	142.1 M	79.3 M	6.2 M	29.5 M	27.1 M	15.5 M	400.5 M	547.1 M	2.3 B
		% Change	**-9.3%**	**-9.0%**	**-17.4%**	**-13.2%**	**-3.4%**	**-12.4%**	**-13.2%**	**-17.7%**	**-7.8%**

Outcome	Time Horizon	Scenario	EUR	Germany	Netherlands	Spain	UK	Australia	Brazil	China	US
KRT only Fossil Fuel Depletion (kg oil eq)	25 Years	Current practice	21.9 B	12.0 B	649.4 M	5.0 B	4.2 B	8.5 B	23.3 B	171.3 B	95.7 B
		Targeted screening every 5 years 75% adherence to GDMT	14.1 B	7.3 B	549.0 M	3.1 B	3.1 B	4.6 B	13.6 B	93.7 B	66.0 B
		% Change	**-35.8%**	**-39.4%**	**-15.5%**	**-38.4%**	**-25.7%**	**-46.0%**	**-41.7%**	**-45.3%**	**-31.1%**
	10 Years	Current practice	7.1 B	3.8 B	261.1 M	1.5 B	1.5 B	3.0 B	6.5 B	41.8 B	34.6 B
		Targeted screening every 5 years 75% adherence to GDMT	6.5 B	3.5 B	222.7 M	1.4 B	1.4 B	2.6 B	5.6 B	34.4 B	31.9 B
		% Change	**-8.6%**	**-8.8%**	**-14.7%**	**-12.2%**	**-3.1%**	**-11.7%**	**-13.0%**	**-17.5%**	**-7.8%**
KRT only Carbon Footprint (kg CO_2 eq)	25 Years	Current practice	56.3 B	33.0 B	1.8 B	12.0 B	9.5 B	2.8 B	68.5 B	485.0 B	231.2 B
		Targeted screening every 5 years 75% adherence to GDMT	35.9 B	20.0 B	1.5 B	7.4 B	7.1 B	1.5 B	39.8 B	265.1 B	159.3 B
		% Change	**-36.3%**	**-39.5%**	**-16.0%**	**-38.6%**	**-25.7%**	**-45.9%**	**-41.9%**	**-45.3%**	**-31.1%**
	10 Years	Current practice	18.1 B	10.5 B	714.9 M	3.7 B	3.3 B	991.2 M	19.0 B	118.2 B	83.8 B
		Targeted screening every 5 years 75% adherence to GDMT	16.5 B	9.5 B	606.7 M	3.2 B	3.2 B	875.3 M	16.5 B	97.5 B	77.2 B
		% Change	**-8.7%**	**-8.8%**	**-15.1%**	**-12.3%**	**-3.1%**	**-11.7%**	**-13.1%**	**-17.5%**	**-7.8%**

[a]Currency conversion for the UK from GBP (£) to euro (€) was performed prior to aggregation across European countries using the 2022 annual average from ECB.[26] Values used in conversions were as follows: £1.0 = €1.173.

[b]Does not include costs associated with CKD treatment or screening.

[c]Does not include costs associated with CKD screening.

Abbreviations: B = billion; CKD = chronic kidney disease; CV = cardiovascular; eq = equivalent; ECB = European Central Bank; EUR = Europe; GBP = pound sterling; GDMT = guideline-directed medical therapy; HHF = hospitalization from heart failure; incl. = including; K = thousand; KRT = kidney replacement therapy; M = million; MI = myocardial infarction; T = trillion; UK = United Kingdoms; US = United States.

Supplementary Table S19. Societal CKD burden over 10 and 25 years following a 25% increase of the diagnosed population compared to current practice.

Outcome	Time Horizon	Scenario	EUR	Germany	Netherlands	Spain	UK	Australia	Brazil	China	US
**Societal Burden**											
Net Workdays (Total Workdays [Stage 3+] – Missed Workdays Due to Absenteeism in CKD Patients)	25 Years	Current practice	10.5 B	3.4 B	787.9 M	2.0 B	4.4 B	1.2 B	21.7 B	63.5 B	27.5 B
		25% increased diagnosis	10.6 B	3.4 B	795.6 M	2.0 B	4.4 B	1.2 B	22.1 B	64.3 B	28.0 B
		% Change	**1.3%**	**1.4%**	**1.0%**	**1.5%**	**1.2%**	**1.1%**	**2.0%**	**1.2%**	**1.8%**
	10 Years	Current practice	3.7 B	1.1 B	290.7 M	625.7 M	1.6 B	441.0 M	8.9 B	37.4 B	12.0 B
		25% increased diagnosis	3.7 B	1.2 B	295.9 M	642.9 M	1.6 B	449.0 M	9.2 B	37.9 B	12.3 B
		% Change	**2.3%**	**2.4%**	**1.8%**	**2.8%**	**2.2%**	**1.8%**	**3.3%**	**1.4%**	**2.8%**
Net Workdays (Total Workdays [Stage 3+] – Missed Workdays Due to Presenteeism in CKD Patients)	25 Years	Current practice	8.8 B	2.8 B	663.1 M	1.7 B	3.7 B	1.0 B	19.5 B	54.8 B	22.9 B
		25% increased diagnosis	8.9 B	2.8 B	667.3 M	1.7 B	3.7 B	1.0 B	19.8 B	55.3 B	23.2 B
		% Change	**1.0%**	**1.1%**	**0.6%**	**1.3%**	**0.9%**	**0.8%**	**1.8%**	**0.9%**	**1.4%**
	10 Years	Current practice	3.1 B	946.0 M	243.9 M	538.1 M	1.3 B	379.3 M	8.0 B	32.4 B	10.0 B
		25% increased diagnosis	3.1 B	964.5 M	247.2 M	551.4 M	1.4 B	384.7 M	8.2 B	32.7 B	10.2 B
		% Change	**1.9%**	**2.0%**	**1.4%**	**2.5%**	**1.8%**	**1.4%**	**3.0%**	**1.1%**	**2.3%**
Net Workdays (Total Workdays [Stage 3+] – Missed Workdays Due to Absenteeism in Caregivers)	25 Years	Current practice	17.2 B	6.2 B	1.5 B	3.8 B	5.7 B	1.9 B	24.8 B	92.2 B	35.1 B
		25% increased diagnosis	17.4 B	6.3 B	1.5 B	3.9 B	5.8 B	1.9 B	25.3 B	93.6 B	35.8 B
		% Change	**1.7%**	**1.5%**	**1.9%**	**2.1%**	**1.6%**	**1.3%**	**2.3%**	**1.5%**	**2.2%**
	10 Years	Current practice	5.4 B	2.0 B	457.3 M	1.1 B	1.9 B	581.3 M	8.8 B	30.8 B	12.5 B
		25% increased diagnosis	5.6 B	2.1 B	474.3 M	1.1 B	1.9 B	595.5 M	9.2 B	31.6 B	13.0 B
		% Change	**3.1%**	**2.6%**	**3.7%**	**4.3%**	**2.9%**	**2.5%**	**4.0%**	**2.7%**	**3.9%**

Outcome	Time Horizon	Scenario	EUR	Germany	Netherlands	Spain	UK	Australia	Brazil	China	US
**Societal Burden**											
Net GDP Contribution (Total GDP [Stage 3+] – Lost GDP due to Absenteeism/ Presenteeism in Patients and Absenteeism in Caregivers)	25 Years	Current practice	15.0 T	4.9 T	1.1 T	2.7 T	6.2 T	1.7 T	35.1 T	93.7 T	38.7 T
		25% increased diagnosis	15.1 T	5.0 T	1.1 T	2.8 T	6.3 T	1.7 T	35.7 T	94.6 T	39.3 T
		% Change	**1.2%**	**1.2%**	**0.8%**	**1.4%**	**1.1%**	**1.0%**	**1.9%**	**1.1%**	**1.6%**
	10 Years	Current practice	5.2 T	1.7 T	399.0 B	876.2 B	2.3 T	637.0 B	14.4 T	55.3 T	16.8 T
		25% increased diagnosis	5.3 T	1.7 T	405.4 B	899.0 B	2.3 T	647.4 B	14.9 T	56.0 T	17.3 T
		% Change	**2.1%**	**2.2%**	**1.6%**	**2.6%**	**2.0%**	**1.6%**	**3.1%**	**1.3%**	**2.6%**
Net FTE (Total FTE [Stage 3+] – Lost FTE due to Absenteeism/ Presenteeism in Patients and Absenteeism in Caregivers)	25 Years	Current practice	127.7 M	48.3 M	11.0 M	24.3 M	44.1 M	12.3 M	134.2 M	609.2 M	279.5 M
		25% increased diagnosis	129.5 M	48.9 M	11.2 M	24.7 M	44.6 M	12.4 M	136.9 M	616.8 M	284.8 M
		% Change	**1.4%**	**1.3%**	**1.5%**	**1.8%**	**1.2%**	**1.0%**	**2.0%**	**1.3%**	**1.9%**
	10 Years	Current practice	41.8 M	16.0 M	3.6 M	7.1 M	15.1 M	4.1 M	51.0 M	259.2 M	108.1 M
		25% increased diagnosis	42.9 M	16.4 M	3.7 M	7.4 M	15.5 M	4.2 M	52.8 M	263.9 M	111.6 M
		% Change	**2.6%**	**2.4%**	**2.9%**	**3.7%**	**2.3%**	**2.0%**	**3.5%**	**1.8%**	**3.2%**
Net Tax Revenue[a] (Total Income [Stage 3+] – Lost Income Due to Absenteeism in CKD Patients and Caregivers * Tax Rate Per Worker)	25 Years	Current practice	€1.8 T	€866.5 B	€79.8 B	€309.4 B	£431.5 B	$111.9 B	R$2.1 T	¥12.4 T	$2.7 T
		25% increased diagnosis	€1.8 T	€879.0 B	€81.1 B	€315.3 B	£437.8 B	$113.2 B	R$2.1 T	¥12.6 T	$2.8 T
		% Change	**1.5%**	**1.5%**	**1.6%**	**1.9%**	**1.5%**	**1.2%**	**2.2%**	**1.4%**	**2.0%**
	10 Years	Current practice	€579.6 B	€287.6 B	€26.1 B	€91.2 B	£149.0 B	$37.5 B	R$784.6 B	¥5.5 T	$1.1 T
		25% increased diagnosis	€595.6 B	€294.9 B	€26.9 B	€94.6 B	£152.8 B	$38.3 B	R$812.9 B	¥5.6 T	$1.1 T
		% Change	**2.8%**	**2.6%**	**3.0%**	**3.7%**	**2.6%**	**2.2%**	**3.6%**	**2.0%**	**3.3%**

[a]Currency conversion for the UK from GBP (£) to euro (€) was performed prior to aggregation across European countries using the 2022 annual average from ECB.[26] Values used in conversions were as follows: £1.0 = €1.173.

Abbreviations: B = billion; CKD = chronic kidney disease; ECB = European Central Bank; EUR = Europe; FTE = full-time equivalent; GBP = pound sterling; GDMT = guideline-directed medical therapy; GDP = gross domestic product; M = million; T = trillion; UK = United Kingdoms; US = United States.

Supplementary Table S20. Societal CKD burden over 10 and 25 years following an increase of GDMT adherence to 75% compared to current practice.

Outcome	Time Horizon	Scenario	EUR	Germany	Netherlands	Spain	UK	Australia	Brazil	China	US
**Societal Burden**											
Net Workdays (Total Workdays [Stage 3+] – Missed Workdays Due to Absenteeism in CKD Patients)	25 Years	Current practice	10.5 B	3.4 B	787.9 M	2.0 B	4.4 B	1.2 B	21.7 B	63.5 B	27.5 B
		75% adherence to GDMT	10.9 B	3.5 B	817.9 M	2.0 B	4.5 B	1.2 B	22.8 B	65.1 B	28.4 B
		% Change	**3.8%**	**4.1%**	**3.8%**	**4.4%**	**3.4%**	**2.0%**	**5.2%**	**2.5%**	**3.2%**
	10 Years	Current practice	3.7 B	1.1 B	290.7 M	625.7 M	1.6 B	441.0 M	8.9 B	37.4 B	12.0 B
		75% adherence to GDMT	3.7 B	1.2 B	297.3 M	643.3 M	1.6 B	444.4 M	9.1 B	37.7 B	12.1 B
		% Change	**2.2%**	**2.1%**	**2.3%**	**2.8%**	**1.9%**	**0.8%**	**1.8%**	**0.9%**	**0.8%**
Net Workdays (Total Workdays [Stage 3+] – Missed Workdays Due to Presenteeism in CKD Patients)	25 Years	Current practice	8.8 B	2.8 B	663.1 M	1.7 B	3.7 B	1.0 B	19.5 B	54.8 B	22.9 B
		75% adherence to GDMT	9.2 B	2.9 B	688.1 M	1.8 B	3.8 B	1.0 B	20.5 B	56.3 B	23.7 B
		% Change	**4.0%**	**4.5%**	**3.8%**	**4.6%**	**3.5%**	**2.3%**	**5.2%**	**2.6%**	**3.4%**
	10 Years	Current practice	3.1 B	946.0 M	243.9 M	538.1 M	1.3 B	379.3 M	8.0 B	32.4 B	10.0 B
		75% adherence to GDMT	3.1 B	967.2 M	249.5 M	554.0 M	1.4 B	382.6 M	8.1 B	32.6 B	10.0 B
		% Change	**2.3%**	**2.2%**	**2.3%**	**2.9%**	**2.0%**	**0.9%**	**1.8%**	**0.9%**	**0.8%**
Net Workdays (Total Workdays [Stage 3+] – Missed Workdays Due to Absenteeism in Caregivers)	25 Years	Current practice	17.2 B	6.2 B	1.5 B	3.8 B	5.7 B	1.9 B	24.8 B	92.2 B	35.1 B
		75% adherence to GDMT	17.6 B	6.4 B	1.5 B	3.9 B	5.8 B	1.9 B	25.9 B	93.9 B	36.4 B
		% Change	**2.7%**	**2.6%**	**3.1%**	**3.4%**	**2.3%**	**1.5%**	**4.7%**	**1.9%**	**3.7%**
	10 Years	Current practice	5.4 B	2.0 B	457.3 M	1.1 B	1.9 B	581.3 M	8.8 B	30.8 B	12.5 B
		75% adherence to GDMT	5.5 B	2.0 B	463.4 M	1.1 B	1.9 B	579.3 M	8.9 B	30.8 B	12.6 B
		% Change	**0.8%**	**0.6%**	**1.3%**	**0.9%**	**0.9%**	**-0.3%**	**1.2%**	**0.2%**	**0.6%**

Outcome	Time Horizon	Scenario	EUR	Germany	Netherlands	Spain	UK	Australia	Brazil	China	US
**Societal Burden**											
Net GDP Contribution (Total GDP [Stage 3+] – Lost GDP due to Absenteeism/ Presenteeism in Patients and Absenteeism in Caregivers)	25 Years	Current practice	15.0 T	4.9 T	1.1 T	2.7 T	6.2 T	1.7 T	35.1 T	93.7 T	38.7 T
		75% adherence to GDMT	15.6 T	5.1 T	1.1 T	2.9 T	6.4 T	1.8 T	36.9 T	96.2 T	40.0 T
		% Change	**4.0%**	**4.3%**	**3.9%**	**4.6%**	**3.5%**	**2.2%**	**5.3%**	**2.7%**	**3.4%**
	10 Years	Current practice	5.2 T	1.7 T	399.0 B	876.2 B	2.3 T	637.0 B	14.4 T	55.3 T	16.8 T
		75% adherence to GDMT	5.3 T	1.7 T	408.4 B	902.2 B	2.3 T	642.6 B	14.7 T	55.8 T	17.0 T
		% Change	**2.3%**	**2.2%**	**2.4%**	**3.0%**	**2.0%**	**0.9%**	**1.8%**	**0.9%**	**0.8%**
Net FTE (Total FTE [Stage 3+] – Lost FTE due to Absenteeism/ Presenteeism in Patients and Absenteeism in Caregivers)	25 Years	Current practice	127.7 M	48.3 M	11.0 M	24.3 M	44.1 M	12.3 M	134.2 M	609.2 M	279.5 M
		75% adherence to GDMT	131.8 M	49.9 M	11.4 M	25.2 M	45.3 M	12.5 M	140.9 M	623.0 M	289.8 M
		% Change	**3.2%**	**3.3%**	**3.4%**	**3.9%**	**2.8%**	**1.8%**	**5.0%**	**2.3%**	**3.7%**
	10 Years	Current practice	41.8 M	16.0 M	3.6 M	7.1 M	15.1 M	4.1 M	51.0 M	259.2 M	108.1 M
		75% adherence to GDMT	42.4 M	16.2 M	3.6 M	7.2 M	15.3 M	4.1 M	51.8 M	260.8 M	108.9 M
		% Change	**1.3%**	**1.1%**	**1.7%**	**1.6%**	**1.3%**	**0.2%**	**1.5%**	**0.6%**	**0.7%**
Net Tax Revenue[a] (Total Income [Stage 3+] – Lost Income Due to Absenteeism in CKD Patients and Caregivers * Tax Rate Per Worker)	25 Years	Current practice	€1.8 T	€866.5 B	€79.8 B	€309.4 B	£431.5 B	$111.9 B	R$2.1 T	¥12.4 T	$2.7 T
		75% adherence to GDMT	€1.8 T	€893.1 B	€82.5 B	€320.8 B	£443.2 B	$113.7 B	R$2.2 T	¥12.7 T	$2.8 T
		% Change	**3.1%**	**3.1%**	**3.3%**	**3.7%**	**2.7%**	**1.6%**	**4.9%**	**2.0%**	**3.4%**
	10 Years	Current practice	€579.6 B	€287.6 B	€26.1 B	€91.2 B	£149.0 B	$37.5 B	R$784.6 B	¥5.5 T	$1.1 T
		75% adherence to GDMT	€587.0 B	€290.7 B	€26.6 B	€92.6 B	£151.0 B	$37.5 B	R$796.0 B	¥5.5 T	$1.1 T
		% Change	**1.3%**	**1.1%**	**1.7%**	**1.6%**	**1.3%**	**0.1%**	**1.5%**	**0.5%**	**0.6%**

[a]Currency conversion for the UK from GBP (£) to euro (€) was performed prior to aggregation across European countries using the 2022 annual average from ECB.[26] Values used in conversions were as follows: £1.0 = €1.173.

Abbreviations: B = billion; CKD = chronic kidney disease; ECB = European Central Bank; EUR = Europe; FTE = full-time equivalent; GBP = pound sterling; GDMT = guideline-directed medical therapy; GDP = gross domestic product; M = million; T = trillion; UK = United Kingdoms; US = United States.

Supplementary Table S21. Societal CKD burden over 10 and 25 years following a 25% increase of diagnosed population and an increase to GDMT adherence to 75% compared to current practice.

Outcome	Time Horizon	Scenario	EUR	Germany	Netherlands	Spain	UK	Australia	Brazil	China	US
**Societal Burden**											
Net Workdays (Total Workdays [Stage 3+] – Missed Workdays Due to Absenteeism in CKD Patients)	25 Years	Current practice	10.5 B	3.4 B	787.9 M	2.0 B	4.4 B	1.2 B	21.7 B	63.5 B	27.5 B
		25% increased diagnosis + 75% adherence to GDMT	11.1 B	3.6 B	828.6 M	2.1 B	4.6 B	1.2 B	23.4 B	66.1 B	29.0 B
		% Change	**5.5%**	**5.8%**	**5.2%**	**6.3%**	**5.0%**	**3.2%**	**8.0%**	**4.1%**	**5.4%**
	10 Years	Current practice	3.7 B	1.1 B	290.7 M	625.7 M	1.6 B	441.0 M	8.9 B	37.4 B	12.0 B
		25% increased diagnosis + 75% adherence to GDMT	3.8 B	1.2 B	303.5 M	663.3 M	1.7 B	452.5 M	9.4 B	38.3 B	12.4 B
		% Change	**4.8%**	**4.8%**	**4.4%**	**6.0%**	**4.4%**	**2.6%**	**5.4%**	**2.5%**	**3.6%**
Net Workdays (Total Workdays [Stage 3+] – Missed Workdays Due to Presenteeism in CKD Patients)	25 Years	Current practice	8.8 B	2.8 B	663.1 M	1.7 B	3.7 B	1.0 B	19.5 B	54.8 B	22.9 B
		25% increased diagnosis + 75% adherence to GDMT	9.3 B	3.0 B	695.0 M	1.8 B	3.9 B	1.1 B	21.0 B	57.0 B	24.1 B
		% Change	**5.5%**	**6.0%**	**4.8%**	**6.3%**	**4.8%**	**3.2%**	**7.8%**	**4.0%**	**5.3%**
	10 Years	Current practice	3.1 B	946.0 M	243.9 M	538.1 M	1.3 B	379.3 M	8.0 B	32.4 B	10.0 B
		25% increased diagnosis + 75% adherence to GDMT	3.2 B	989.6 M	253.7 M	569.9 M	1.4 B	388.3 M	8.4 B	33.1 B	10.3 B
		% Change	**4.6%**	**4.6%**	**4.1%**	**5.9%**	**4.1%**	**2.4%**	**5.1%**	**2.2%**	**3.3%**

Outcome	Time Horizon	Scenario	EUR	Germany	Netherlands	Spain	UK	Australia	Brazil	China	US
**Societal Burden**											
Net Workdays (Total Workdays [Stage 3+] – Missed Workdays Due to Absenteeism in Caregivers)	25 Years	Current practice	17.2 B	6.2 B	1.5 B	3.8 B	5.7 B	1.9 B	24.8 B	92.2 B	35.1 B
		25% increased diagnosis + 75% adherence to GDMT	18.0 B	6.5 B	1.6 B	4.0 B	5.9 B	1.9 B	26.7 B	95.6 B	37.3 B
		% Change	**4.8%**	**4.5%**	**5.6%**	**5.9%**	**4.2%**	**2.9%**	**7.7%**	**3.7%**	**6.5%**
	10 Years	Current practice	5.4 B	2.0 B	457.3 M	1.1 B	1.9 B	581.3 M	8.8 B	30.8 B	12.5 B
		25% increased diagnosis + 75% adherence to GDMT	5.7 B	2.1 B	482.0 M	1.1 B	1.9 B	593.2 M	9.3 B	31.7 B	13.1 B
		% Change	**4.1%**	**3.4%**	**5.4%**	**5.4%**	**3.9%**	**2.1%**	**5.4%**	**2.9%**	**4.5%**
Net GDP Contribution (Total GDP [Stage 3+] – Lost GDP due to Absenteeism/ Presenteeism in Patients and Absenteeism in Caregivers)	25 Years	Current practice	15.0 T	4.9 T	1.1 T	2.7 T	6.2 T	1.7 T	35.1 T	93.7 T	38.7 T
		25% increased diagnosis + 75% adherence to GDMT	15.8 T	5.2 T	1.1 T	2.9 T	6.5 T	1.8 T	37.9 T	97.6 T	40.8 T
		% Change	**5.6%**	**6.0%**	**5.1%**	**6.5%**	**5.0%**	**3.3%**	**8.0%**	**4.2%**	**5.5%**
	10 Years	Current practice	5.2 T	1.7 T	399.0 B	876.2 B	2.3 T	637.0 B	14.4 T	55.3 T	16.8 T
		25% increased diagnosis + 75% adherence to GDMT	5.5 T	1.8 T	416.2 B	929.2 B	2.4 T	653.3 B	15.2 T	56.6 T	17.4 T
		% Change	**4.8%**	**4.7%**	**4.3%**	**6.1%**	**4.3%**	**2.6%**	**5.3%**	**2.4%**	**3.5%**

Outcome	Time Horizon	Scenario	EUR	Germany	Netherlands	Spain	UK	Australia	Brazil	China	US
**Societal Burden**											
Net FTE (Total FTE [Stage 3+] – Lost FTE due to Absenteeism/ Presenteeism in Patients and Absenteeism in Caregivers)	25 Years	Current practice	127.7 M	48.3 M	11.0 M	24.3 M	44.1 M	12.3 M	134.2 M	609.2 M	279.5 M
		25% increased diagnosis + 75% adherence to GDMT	134.1 M	50.7 M	11.6 M	25.8 M	46.0 M	12.7 M	144.7 M	632.8 M	296.5 M
		% Change	**5.0%**	**5.0%**	**5.3%**	**6.1%**	**4.4%**	**3.1%**	**7.8%**	**3.9%**	**6.1%**
	10 Years	Current practice	41.8 M	16.0 M	3.6 M	7.1 M	15.1 M	4.1 M	51.0 M	259.2 M	108.1 M
		25% increased diagnosis + 75% adherence to GDMT	43.6 M	16.6 M	3.7 M	7.5 M	15.7 M	4.2 M	53.7 M	265.7 M	112.5 M
		% Change	**4.2%**	**3.7%**	**4.9%**	**5.6%**	**3.9%**	**2.2%**	**5.3%**	**2.5%**	**4.0%**
Net Tax Revenue[a] (Total Income [Stage 3+] – Lost Income Due to Absenteeism in CKD Patients and Caregivers * Tax Rate Per Worker)	25 Years	Current practice	€1.8T	€866.5 B	€79.8 B	€309.4 B	£431.5 B	$111.9 B	R$2.1 T	¥12.4 T	$2.7 T
		25% increased diagnosis + 75% adherence to GDMT	€1.8T	€908.9 B	€84.1 B	€327.9 B	£451.0 B	$115.2 B	R$2.2 T	¥12.9 T	$2.9 T
		% Change	**5.0%**	**4.9%**	**5.4%**	**6.0%**	**4.5%**	**3.0%**	**7.8%**	**3.8%**	**5.9%**
	10 Years	Current practice	€579.6 B	€287.6 B	€26.1 B	€91.2 B	£149.0 B	$37.5 B	R$784.6 B	¥5.5 T	$1.1 T
		25% increased diagnosis + 75% adherence to GDMT	€604.5 B	€298.8 B	€27.4 B	€96.3 B	£155.1 B	$38.3 B	R$826.8 B	¥5.6 T	$1.1 T
		% Change	**4.3%**	**3.9%**	**5.0%**	**5.6%**	**4.1%**	**2.3%**	**5.4%**	**2.6%**	**4.1%**

[a]Currency conversion for the UK from GBP (£) to euro (€) was performed prior to aggregation across European countries using the 2022 annual average from ECB.[26] Values used in conversions were as follows: £1.0 = €1.173.

Abbreviations: B = billion; CKD = chronic kidney disease; ECB = European Central Bank; EUR = Europe; FTE = full-time equivalent; GBP = pound sterling; GDMT = guideline-directed medical therapy; GDP = gross domestic product; M = million; T = trillion; UK = United Kingdoms; US = United States.

Supplementary Table S22. Societal CKD burden over 10 and 25 years following annual targeted screening of high-risk population and an increase to GDMT adherence to 75% compared to current practice.

Outcome	Time Horizon	Scenario	EUR	Germany	Netherlands	Spain	UK	Australia	Brazil	China	US
**Societal Burden**											
Net Workdays (Total Workdays [Stage 3+] – Missed Workdays Due to Absenteeism in CKD Patients)	25 Years	Current practice	10.5 B	3.4 B	787.9 M	2.0 B	4.4 B	1.2 B	21.7 B	63.5 B	27.5 B
		Targeted screening + 75% adherence to GDMT	10.6 B	3.4 B	782.6 M	2.0 B	4.5 B	1.2 B	23.4 B	64.6 B	29.1 B
		% Change	**1.1%**	**-0.1%**	**-0.7%**	**0.5%**	**2.7%**	**-0.3%**	**7.8%**	**1.8%**	**5.8%**
	10 Years	Current practice	3.7 B	1.1 B	290.7 M	625.7 M	1.6 B	441.0 M	8.9 B	37.4 B	12.0 B
		Targeted screening + 75% adherence to GDMT	3.7 B	1.1 B	284.2 M	618.1 M	1.6 B	450.1 M	9.6 B	37.9 B	12.8 B
		% Change	**0.3%**	**-2.6%**	**-2.2%**	**-1.2%**	**3.5%**	**2.1%**	**7.2%**	**1.4%**	**7.3%**
Net Workdays (Total Workdays [Stage 3+] – Missed Workdays Due to Presenteeism in CKD Patients)	25 Years	Current practice	8.8 B	2.8 B	663.1 M	1.7 B	3.7 B	1.0 B	19.5 B	54.8 B	22.9 B
		Targeted screening + 75% adherence to GDMT	8.9 B	2.8 B	653.8 M	1.7 B	3.8 B	1.0 B	20.9 B	55.5 B	24.1 B
		% Change	**0.7%**	**-0.4%**	**-1.4%**	**0.2%**	**2.1%**	**-0.7%**	**7.5%**	**1.3%**	**5.5%**
	10 Years	Current practice	3.1 B	946.0 M	243.9 M	538.1 M	1.3 B	379.3 M	8.0 B	32.4 B	10.0 B
		Targeted screening + 75% adherence to GDMT	3.1 B	914.3 M	236.4 M	528.9 M	1.4 B	384.6 M	8.5 B	32.6 B	10.6 B
		% Change	**-0.4%**	**-3.3%**	**-3.1%**	**-1.7%**	**2.7%**	**1.4%**	**6.8%**	**0.6%**	**6.7%**

Outcome	Time Horizon	Scenario	EUR	Germany	Netherlands	Spain	UK	Australia	Brazil	China	US
**Societal Burden**											
Net Workdays (Total Workdays [Stage 3+] – Missed Workdays Due to Absenteeism in Caregivers)	25 Years	Current practice	17.2 B	6.2 B	1.5 B	3.8 B	5.7 B	1.9 B	24.8 B	92.2 B	35.1 B
		Targeted screening + 75% adherence to GDMT	18.4 B	6.6 B	1.6 B	4.1 B	6.0 B	1.9 B	27.7 B	97.1 B	38.6 B
		% Change	**7.1%**	**6.6%**	**8.1%**	**8.5%**	**6.5%**	**2.7%**	**12.0%**	**5.3%**	**10.0%**
	10 Years	Current practice	5.4 B	2.0 B	457.3 M	1.1 B	1.9 B	581.3 M	8.8 B	30.8 B	12.5 B
		Targeted screening + 75% adherence to GDMT	6.0 B	2.2 B	513.8 M	1.2 B	2.1 B	633.4 M	10.1 B	33.6 B	14.2 B
		% Change	**10.7%**	**9.6%**	**12.4%**	**12.7%**	**10.5%**	**9.0%**	**13.9%**	**9.0%**	**14.0%**
Net GDP Contribution (Total GDP [Stage 3+] – Lost GDP due to Absenteeism/ Presenteeism in Patients and Absenteeism in Caregivers)	25 Years	Current practice	15.0 T	4.9 T	1.1 T	2.7 T	6.2 T	1.7 T	35.1 T	93.7 T	38.7 T
		Targeted screening + 75% adherence to GDMT	15.1 T	4.9 T	1.1 T	2.8 T	6.4 T	1.7 T	37.8 T	95.2 T	40.9 T
		% Change	**1.0%**	**-0.2%**	**-0.9%**	**0.5%**	**2.5%**	**-0.4%**	**7.8%**	**1.7%**	**5.8%**
	10 Years	Current practice	5.2 T	1.7 T	399.0 B	876.2 B	2.3 T	637.0 B	14.4 T	55.3 T	16.8 T
		Targeted screening + 75% adherence to GDMT	5.2 T	1.6 T	388.8 B	864.0 B	2.3 T	648.6 B	15.4 T	55.9 T	18.0 T
		% Change	**0.0%**	**-2.9%**	**-2.6%**	**-1.4%**	**3.2%**	**1.8%**	**7.1%**	**1.1%**	**7.1%**

Outcome	Time Horizon	Scenario	EUR	Germany	Netherlands	Spain	UK	Australia	Brazil	China	US
**Societal Burden**											
Net FTE (Total FTE [Stage 3+] – Lost FTE due to Absenteeism/ Presenteeism in Patients and Absenteeism in Caregivers)	25 Years	Current practice	127.7 M	48.3 M	11.0 M	24.3 M	44.1 M	12.3 M	134.2 M	609.2 M	279.5 M
		Targeted screening + 75% adherence to GDMT	134.1 M	50.6 M	11.6 M	25.8 M	46.2 M	12.5 M	147.7 M	632.8 M	302.6 M
		% Change	**5.0%**	**4.6%**	**5.3%**	**6.1%**	**4.8%**	**1.5%**	**10.1%**	**3.9%**	**8.3%**
	10 Years	Current practice	41.8 M	16.0 M	3.6 M	7.1 M	15.1 M	4.1 M	51.0 M	259.2 M	108.1 M
		Targeted screening + 75% adherence to GDMT	44.7 M	17.0 M	3.8 M	7.7 M	16.2 M	4.3 M	56.4 M	271.6 M	119.8 M
		% Change	**6.8%**	**5.7%**	**7.2%**	**8.1%**	**7.2%**	**6.0%**	**10.6%**	**4.8%**	**10.8%**
Net Tax Revenue[a] (Total Income [Stage 3+] – Lost Income Due to Absenteeism in CKD Patients and Caregivers * Tax Rate Per Worker)	25 Years	Current practice	€1.8 T	€866.5 B	€79.8 B	€309.4 B	£431.5 B	$111.9 B	R$2.1 T	¥12.4 T	$2.7 T
		Targeted screening + 75% adherence to GDMT	€1.8 T	€902.6 B	€83.8 B	€326.9 B	£452.3 B	$113.5 B	R$2.3 T	¥12.9 T	$2.9 T
		% Change	**4.7%**	**4.2%**	**5.0%**	**5.7%**	**4.8%**	**1.4%**	**10.0%**	**3.8%**	**8.0%**
	10 Years	Current practice	€579.6 B	€287.6 B	€26.1 B	€91.2 B	£149.0 B	$37.5 B	R$784.6 B	¥5.5 T	$1.1 T
		Targeted screening + 75% adherence to GDMT	€615.5 B	€302.3 B	€27.8 B	€97.9 B	£159.8 B	$39.7 B	R$866.9 B	¥5.7 T	$1.2 T
		% Change	**6.2%**	**5.1%**	**6.6%**	**7.4%**	**7.2%**	**5.9%**	**10.5%**	**4.8%**	**10.6%**

[a]Currency conversion for the UK from GBP (£) to euro (€) was performed prior to aggregation across European countries using the 2022 annual average from ECB.[26] Values used in conversions were as follows: £1.0 = €1.173.

Abbreviations: B = billion; CKD = chronic kidney disease; ECB = European Central Bank; EUR = Europe; FTE = full-time equivalent; GBP = pound sterling; GDMT = guideline-directed medical therapy; GDP = gross domestic product; M = million; T = trillion; UK = United Kingdoms; US = United States.

Supplementary Table S23. Societal CKD burden over 10 and 25 years following an increase to 60% adherence to GDMT compared to current practice.

Outcome	Time Horizon	Scenario	EUR	Germany	Netherlands	Spain	UK	Australia	Brazil	China	US
**Societal Burden**											
Net Workdays (Total Workdays [Stage 3+] – Missed Workdays Due to Absenteeism in CKD Patients)	25 Years	Current practice	10.5 B	3.4 B	787.9 M	2.0 B	4.4 B	1.2 B	21.7 B	63.5 B	27.5 B
		60% adherence to GDMT	10.8 B	3.5 B	811.2 M	2.0 B	4.5 B	1.2 B	22.6 B	64.9 B	28.3 B
		% Change	**3.0%**	**3.2%**	**3.0%**	**3.5%**	**2.6%**	**1.5%**	**4.3%**	**2.3%**	**2.8%**
	10 Years	Current practice	3.7 B	1.1 B	290.7 M	625.7 M	1.6 B	441.0 M	8.9 B	37.4 B	12.0 B
		60% adherence to GDMT	3.7 B	1.2 B	295.6 M	639.1 M	1.6 B	443.5 M	9.0 B	37.6 B	12.0 B
		% Change	**1.7%**	**1.6%**	**1.7%**	**2.1%**	**1.5%**	**0.6%**	**1.4%**	**0.7%**	**0.6%**
Net Workdays (Total Workdays [Stage 3+] – Missed Workdays Due to Presenteeism in CKD Patients)	25 Years	Current practice	8.8 B	2.8 B	663.1 M	1.7 B	3.7 B	1.0 B	19.5 B	54.8 B	22.9 B
		60% adherence to GDMT	9.1 B	2.9 B	682.4 M	1.7 B	3.8 B	1.0 B	20.3 B	56.1 B	23.6 B
		% Change	**3.1%**	**3.5%**	**2.9%**	**3.6%**	**2.7%**	**1.8%**	**4.3%**	**2.3%**	**2.9%**
	10 Years	Current practice	3.1 B	946.0 M	243.9 M	538.1 M	1.3 B	379.3 M	8.0 B	32.4 B	10.0 B
		60% adherence to GDMT	3.1 B	962.5 M	248.1 M	550.1 M	1.4 B	381.8 M	8.1 B	32.6 B	10.0 B
		% Change	**1.7%**	**1.7%**	**1.7%**	**2.2%**	**1.6%**	**0.7%**	**1.3%**	**0.7%**	**0.6%**
Net Workdays (Total Workdays [Stage 3+] – Missed Workdays Due to Absenteeism in Caregivers)	25 Years	Current practice	17.2 B	6.2 B	1.5 B	3.8 B	5.7 B	1.9 B	24.8 B	92.2 B	35.1 B
		60% adherence to GDMT	17.5 B	6.3 B	1.5 B	3.9 B	5.8 B	1.9 B	25.7 B	93.7 B	36.2 B
		% Change	**2.2%**	**2.1%**	**2.6%**	**2.8%**	**1.9%**	**1.1%**	**3.8%**	**1.6%**	**3.1%**
	10 Years	Current practice	5.4 B	2.0 B	457.3 M	1.1 B	1.9 B	581.3 M	8.8 B	30.8 B	12.5 B
		60% adherence to GDMT	5.5 B	2.0 B	462.3 M	1.1 B	1.9 B	579.1 M	8.9 B	30.9 B	12.6 B
		% Change	**0.7%**	**0.4%**	**1.1%**	**0.7%**	**0.7%**	**-0.4%**	**0.9%**	**0.3%**	**0.5%**

Outcome	Time Horizon	Scenario	EUR	Germany	Netherlands	Spain	UK	Australia	Brazil	China	US
**Societal Burden**											
Net GDP Contribution (Total GDP [Stage 3+] – Lost GDP due to Absenteeism/ Presenteeism in Patients and Absenteeism in Caregivers)	25 Years	Current practice	15.0 T	4.9 T	1.1 T	2.7 T	6.2 T	1.7 T	35.1 T	93.7 T	38.7 T
		60% adherence to GDMT	15.4 T	5.1 T	1.1 T	2.8 T	6.4 T	1.8 T	36.6 T	95.9 T	39.8 T
		% Change	**3.1%**	**3.4%**	**3.0%**	**3.7%**	**2.7%**	**1.7%**	**4.4%**	**2.4%**	**3.0%**
	10 Years	Current practice	5.2 T	1.7 T	399.0 B	876.2 B	2.3 T	637.0 B	14.4 T	55.3 T	16.8 T
		60% adherence to GDMT	5.3 T	1.7 T	406.1 B	895.9 B	2.3 T	641.2 B	14.6 T	55.7 T	17.0 T
		% Change	**1.7%**	**1.7%**	**1.8%**	**2.3%**	**1.6%**	**0.7%**	**1.4%**	**0.7%**	**0.6%**
Net FTE (Total FTE [Stage 3+] – Lost FTE due to Absenteeism/ Presenteeism in Patients and Absenteeism in Caregivers)	25 Years	Current practice	127.7 M	48.3 M	11.0 M	24.3 M	44.1 M	12.3 M	134.2 M	609.2 M	279.5 M
		60% adherence to GDMT	131.0 M	49.6 M	11.3 M	25.0 M	45.1 M	12.4 M	139.7 M	621.1 M	288.2 M
		% Change	**2.6%**	**2.6%**	**2.7%**	**3.1%**	**2.2%**	**1.4%**	**4.1%**	**2.0%**	**3.1%**
	10 Years	Current practice	41.8 M	16.0 M	3.6 M	7.1 M	15.1 M	4.1 M	51.0 M	259.2 M	108.1 M
		60% adherence to GDMT	42.3 M	16.2 M	3.6 M	7.2 M	15.3 M	4.1 M	51.6 M	260.6 M	108.8 M
		% Change	**1.1%**	**0.9%**	**1.3%**	**1.2%**	**1.1%**	**0.1%**	**1.1%**	**0.5%**	**0.6%**
Net Tax Revenue[a] (Total Income [Stage 3+] – Lost Income Due to Absenteeism in CKD Patients and Caregivers * Tax Rate Per Worker)	25 Years	Current practice	€1.8 T	€866.5 B	€79.8 B	€309.4 B	£431.5 B	$111.9 B	R$2.1 T	¥12.4 T	$2.7 T
		60% adherence to GDMT	€1.8 T	€887.3 B	€81.9 B	€318.6 B	£440.9 B	$113.2 B	R$2.1 T	¥12.6 T	$2.8 T
		% Change	**2.5%**	**2.4%**	**2.7%**	**3.0%**	**2.2%**	**1.2%**	**4.0%**	**1.8%**	**2.9%**
	10 Years	Current practice	€579.6 B	€287.6 B	€26.1 B	€91.2 B	£149.0 B	$37.5 B	R$784.6 B	¥5.5 T	$1.1 T
		60% adherence to GDMT	€585.4 B	€290.0 B	€26.5 B	€92.3 B	£150.6 B	$37.5 B	R$793.1 B	¥5.5 T	$1.1 T
		% Change	**1.0%**	**0.8%**	**1.3%**	**1.2%**	**1.1%**	**0.0%**	**1.1%**	**0.5%**	**0.5%**

[a]Currency conversion for the UK from GBP (£) to euro (€) was performed prior to aggregation across European countries using the 2022 annual average from ECB.[26] Values used in conversions were as follows: £1.0 = €1.173.

Abbreviations: B = billion; CKD = chronic kidney disease; ECB = European Central Bank; EUR = Europe; FTE = full-time equivalent; GBP = pound sterling; GDMT = guideline-directed medical therapy; GDP = gross domestic product; M = million; T = trillion; UK = United Kingdoms; US = United States.

Supplementary Table S24. Societal CKD burden over 10 and 25 years following an increase to 90% adherence to GDMT compared to current practice.

Outcome	Time Horizon	Scenario	EUR	Germany	Netherlands	Spain	UK	Australia	Brazil	China	US
**Societal Burden**											
Net Workdays (Total Workdays [Stage 3+] – Missed Workdays Due to Absenteeism in CKD Patients)	25 Years	Current practice	10.5 B	3.4 B	787.9 M	2.0 B	4.4 B	1.2 B	21.7 B	63.5 B	27.5 B
		90% adherence to GDMT	11.0 B	3.5 B	823.4 M	2.1 B	4.5 B	1.2 B	22.9 B	65.2 B	28.5 B
		% Change	**4.5%**	**4.9%**	**4.5%**	**5.2%**	**3.9%**	**2.3%**	**5.8%**	**2.7%**	**3.6%**
	10 Years	Current practice	3.7 B	1.1 B	290.7 M	625.7 M	1.6 B	441.0 M	8.9 B	37.4 B	12.0 B
		90% adherence to GDMT	3.8 B	1.2 B	298.8 M	647.8 M	1.6 B	445.4 M	9.1 B	37.7 B	12.1 B
		% Change	**2.7%**	**2.6%**	**2.8%**	**3.5%**	**2.4%**	**1.0%**	**2.2%**	**1.0%**	**1.0%**
Net Workdays (Total Workdays [Stage 3+] – Missed Workdays Due to Presenteeism in CKD Patients)	25 Years	Current practice	8.8 B	2.8 B	663.1 M	1.7 B	3.7 B	1.0 B	19.5 B	54.8 B	22.9 B
		90% adherence to GDMT	9.2 B	2.9 B	692.7 M	1.8 B	3.8 B	1.1 B	20.6 B	56.4 B	23.8 B
		% Change	**4.7%**	**5.3%**	**4.5%**	**5.4%**	**4.1%**	**2.6%**	**5.8%**	**2.8%**	**3.8%**
	10 Years	Current practice	3.1 B	946.0 M	243.9 M	538.1 M	1.3 B	379.3 M	8.0 B	32.4 B	10.0 B
		90% adherence to GDMT	3.2 B	972.4 M	250.8 M	558.0 M	1.4 B	383.6 M	8.2 B	32.7 B	10.1 B
		% Change	**2.8%**	**2.8%**	**2.9%**	**3.7%**	**2.5%**	**1.1%**	**2.2%**	**1.0%**	**1.0%**
Net Workdays (Total Workdays [Stage 3+] – Missed Workdays Due to Absenteeism in Caregivers)	25 Years	Current practice	17.2 B	6.2 B	1.5 B	3.8 B	5.7 B	1.9 B	24.8 B	92.2 B	35.1 B
		90% adherence to GDMT	17.7 B	6.4 B	1.6 B	3.9 B	5.8 B	1.9 B	26.1 B	94.1 B	36.5 B
		% Change	**3.1%**	**2.9%**	**3.5%**	**3.8%**	**2.6%**	**1.8%**	**5.2%**	**2.0%**	**4.1%**
	10 Years	Current practice	5.4 B	2.0 B	457.3 M	1.1 B	1.9 B	581.3 M	8.8 B	30.8 B	12.5 B
		90% adherence to GDMT	5.5 B	2.1 B	464.4 M	1.1 B	1.9 B	580.2 M	9.0 B	30.9 B	12.6 B
		% Change	**1.0%**	**0.7%**	**1.6%**	**1.2%**	**1.1%**	**-0.2%**	**1.5%**	**0.3%**	**0.7%**

Outcome	Time Horizon	Scenario	EUR	Germany	Netherlands	Spain	UK	Australia	Brazil	China	US
**Societal Burden**											
Net GDP Contribution (Total GDP [Stage 3+] – Lost GDP due to Absenteeism/ Presenteeism in Patients and Absenteeism in Caregivers)	25 Years	Current practice	15.0 T	4.9 T	1.1 T	2.7 T	6.2 T	1.7 T	35.1 T	93.7 T	38.7 T
		90% adherence to GDMT	15.7 T	5.2 T	1.1 T	2.9 T	6.5 T	1.8 T	37.1 T	96.3 T	40.1 T
		% Change	**4.7%**	**5.1%**	**4.6%**	**5.5%**	**4.1%**	**2.6%**	**5.9%**	**2.9%**	**3.8%**
	10 Years	Current practice	5.2 T	1.7 T	399.0 B	876.2 B	2.3 T	637.0 B	14.4 T	55.3 T	16.8 T
		90% adherence to GDMT	5.4 T	1.7 T	410.6 B	908.6 B	2.3 T	644.1 B	14.7 T	55.9 T	17.0 T
		% Change	**2.8%**	**2.7%**	**2.9%**	**3.7%**	**2.5%**	**1.1%**	**2.2%**	**1.0%**	**1.0%**
Net FTE (Total FTE [Stage 3+] – Lost FTE due to Absenteeism/ Presenteeism in Patients and Absenteeism in Caregivers)	25 Years	Current practice	127.7 M	48.3 M	11.0 M	24.3 M	44.1 M	12.3 M	134.2 M	609.2 M	279.5 M
		90% adherence to GDMT	132.4 M	50.1 M	11.4 M	25.3 M	45.5 M	12.5 M	141.6 M	624.1 M	290.9 M
		% Change	**3.7%**	**3.7%**	**3.8%**	**4.4%**	**3.2%**	**2.1%**	**5.6%**	**2.5%**	**4.1%**
	10 Years	Current practice	41.8 M	16.0 M	3.6 M	7.1 M	15.1 M	4.1 M	51.0 M	259.2 M	108.1 M
		90% adherence to GDMT	42.5 M	16.3 M	3.6 M	7.3 M	15.4 M	4.1 M	52.0 M	261.1 M	109.1 M
		% Change	**1.6%**	**1.4%**	**2.0%**	**2.0%**	**1.7%**	**0.4%**	**1.9%**	**0.7%**	**0.9%**
Net Tax Revenue[a] (Total Income [Stage 3+] – Lost Income Due to Absenteeism in CKD Patients and Caregivers * Tax Rate Per Worker)	25 Years	Current practice	€1.8 T	€866.5 B	€79.8 B	€309.4 B	£431.5 B	$111.9 B	R$2.1 T	¥12.4 T	$2.7 T
		90% adherence to GDMT	€1.8 T	€897.2 B	€82.8 B	€322.4 B	£445.1 B	$114.0 B	R$2.2 T	¥12.7 T	$2.8 T
		% Change	**3.6%**	**3.5%**	**3.8%**	**4.2%**	**3.1%**	**1.9%**	**5.4%**	**2.2%**	**3.7%**
	10 Years	Current practice	€579.6 B	€287.6 B	€26.1 B	€91.2 B	£149.0 B	$37.5 B	R$784.6 B	¥5.5 T	$1.1 T
		90% adherence to GDMT	€588.9 B	€291.5 B	€26.7 B	€93.0 B	£151.5 B	$37.6 B	R$798.6 B	¥5.5 T	$1.1 T
		% Change	**1.6%**	**1.4%**	**2.0%**	**2.0%**	**1.7%**	**0.3%**	**1.8%**	**0.6%**	**0.8%**

[a]Currency conversion for the UK from GBP (£) to euro (€) was performed prior to aggregation across European countries using the 2022 annual average from ECB.[26] Values used in conversions were as follows: £1.0 = €1.173.

Abbreviations: B = billion; CKD = chronic kidney disease; ECB = European Central Bank; EUR = Europe; FTE = full-time equivalent; GBP = pound sterling; GDMT = guideline-directed medical therapy; GDP = gross domestic product; M = million; T = trillion; UK = United Kingdoms; US = United States.

Supplementary Table S25. Societal CKD burden over 10 and 25 years following targeted screening every 3 years for high-risk populations and a 75% adherence to GDMT compared to current practice.

Outcome	Time Horizon	Scenario	EUR	Germany	Netherlands	Spain	UK	Australia	Brazil	China	US
**Societal Burden**											
Net Workdays (Total Workdays [Stage 3+] – Missed Workdays Due to Absenteeism in CKD Patients)	25 Years	Current practice	10.5 B	3.4 B	787.9 M	2.0 B	4.4 B	1.2 B	21.7 B	63.5 B	27.5 B
		Targeted screening every 3 years + 75% adherence to GDMT	10.4 B	3.3 B	771.3 M	1.9 B	4.4 B	1.2 B	23.0 B	64.0 B	28.7 B
		% Change	**-0.7%**	**-2.0%**	**-2.1%**	**-1.6%**	**1.0%**	**-1.7%**	**6.1%**	**0.8%**	**4.6%**
	10 Years	Current practice	3.7 B	1.1 B	290.7 M	625.7 M	1.6 B	441.0 M	8.9 B	37.4 B	12.0 B
		Targeted screening every 3 years + 75% adherence to GDMT	3.6 B	1.1 B	278.0 M	596.1 M	1.6 B	440.9 M	9.4 B	37.4 B	12.6 B
		% Change	**-2.3%**	**-5.2%**	**-4.4%**	**-4.7%**	**1.2%**	**0.0%**	**5.2%**	**0.0%**	**5.6%**

Outcome	Time Horizon	Scenario	EUR	Germany	Netherlands	Spain	UK	Australia	Brazil	China	US
**Societal Burden**											
Net Workdays (Total Workdays [Stage 3+] – Missed Workdays Due to Presenteeism in CKD Patients)	25 Years	Current practice	8.8 B	2.8 B	663.1 M	1.7 B	3.7 B	1.0 B	19.5 B	54.8 B	22.9 B
		Targeted screening every 3 years + 75% adherence to GDMT	8.7 B	2.7 B	644.5 M	1.7 B	3.7 B	1.0 B	20.6 B	55.0 B	23.8 B
		% Change	**-1.1%**	**-2.3%**	**-2.8%**	**-1.9%**	**0.4%**	**-2.1%**	**5.8%**	**0.3%**	**4.1%**
	10 Years	Current practice	3.1 B	946.0 M	243.9 M	538.1 M	1.3 B	379.3 M	8.0 B	32.4 B	10.0 B
		Targeted screening every 3 years + 75% adherence to GDMT	3.0 B	889.5 M	231.2 M	510.0 M	1.3 B	376.6 M	8.4 B	32.1 B	10.5 B
		% Change	**-3.0%**	**-6.0%**	**-5.2%**	**-5.2%**	**0.4%**	**-0.7%**	**4.8%**	**-0.7%**	**5.0%**
Net Workdays (Total Workdays [Stage 3+] – Missed Workdays Due to Absenteeism in Caregivers)	25 Years	Current practice	17.2 B	6.2 B	1.5 B	3.8 B	5.7 B	1.9 B	24.8 B	92.2 B	35.1 B
		Targeted screening every 3 years + 75% adherence to GDMT	18.2 B	6.6 B	1.6 B	4.1 B	6.0 B	1.9 B	27.6 B	96.5 B	38.4 B
		% Change	**6.3%**	**5.7%**	**7.3%**	**7.6%**	**5.7%**	**2.2%**	**11.3%**	**4.7%**	**9.5%**
	10 Years	Current practice	5.4 B	2.0 B	457.3 M	1.1 B	1.9 B	581.3 M	8.8 B	30.8 B	12.5 B
		Targeted screening every 3 years + 75% adherence to GDMT	5.9 B	2.2 B	507.0 M	1.2 B	2.0 B	628.0 M	10.0 B	33.3 B	14.2 B
		% Change	**9.5%**	**8.5%**	**10.9%**	**11.2%**	**9.2%**	**8.0%**	**12.9%**	**8.1%**	**13.4%**

Outcome	Time Horizon	Scenario	EUR	Germany	Netherlands	Spain	UK	Australia	Brazil	China	US
**Societal Burden**											
Net GDP Contribution (Total GDP [Stage 3+] – Lost GDP due to Absenteeism/ Presenteeism in Patients and Absenteeism in Caregivers)	25 Years	Current practice	15.0 T	4.9 T	1.1 T	2.7 T	6.2 T	1.7 T	35.1 T	93.7 T	38.7 T
		Targeted screening every 3 years + 75% adherence to GDMT	14.8 T	4.8 T	1.1 T	2.7 T	6.3 T	1.7 T	37.2 T	94.3 T	40.4 T
		% Change	**-0.8%**	**-2.0%**	**-2.4%**	**-1.6%**	**0.8%**	**-1.8%**	**6.1%**	**0.7%**	**4.5%**
	10 Years	Current practice	5.2 T	1.7 T	399.0 B	876.2 B	2.3 T	637.0 B	14.4 T	55.3 T	16.8 T
		Targeted screening every 3 years + 75% adherence to GDMT	5.1 T	1.6 T	380.2 B	833.1 B	2.3 T	635.1 B	15.1 T	55.1 T	17.8 T
		% Change	**-2.6%**	**-5.6%**	**-4.7%**	**-4.9%**	**0.9%**	**-0.3%**	**5.0%**	**-0.3%**	**5.4%**
Net FTE (Total FTE [Stage 3+] – Lost FTE due to Absenteeism/ Presenteeism in Patients and Absenteeism in Caregivers)	25 Years	Current practice	127.7 M	48.3 M	11.0 M	24.3 M	44.1 M	12.3 M	134.2 M	609.2 M	279.5 M
		Targeted screening every 3 years + 75% adherence to GDMT	132.6 M	50.0 M	11.5 M	25.5 M	45.7 M	12.4 M	146.2 M	628.3 M	300.4 M
		% Change	**3.9%**	**3.5%**	**4.3%**	**4.9%**	**3.7%**	**0.7%**	**9.0%**	**3.2%**	**7.5%**
	10 Years	Current practice	41.8 M	16.0 M	3.6 M	7.1 M	15.1 M	4.1 M	51.0 M	259.2 M	108.1 M
		Targeted screening every 3 years + 75% adherence to GDMT	44.0 M	16.7 M	3.8 M	7.5 M	16.0 M	4.3 M	55.7 M	268.7 M	118.7 M
		% Change	**5.1%**	**4.2%**	**5.6%**	**5.9%**	**5.6%**	**4.7%**	**9.1%**	**3.7%**	**9.7%**

Outcome	Time Horizon	Scenario	EUR	Germany	Netherlands	Spain	UK	Australia	Brazil	China	US
**Societal Burden**											
Net Tax Revenue[a] (Total Income [Stage 3+] – Lost Income Due to Absenteeism in CKD Patients and Caregivers * Tax Rate Per Worker)	25 Years	Current practice	€1.8 T	€866.5 B	€79.8 B	€309.4 B	£431.5 B	$111.9 B	R$2.1 T	¥12.4 T	$2.7 T
		Targeted screening every 3 years + 75% adherence to GDMT	€1.8 T	€891.8 B	€82.9 B	€322.8 B	£447.2 B	$112.5 B	R$2.2 T	¥12.8 T	$2.9 T
		% Change	**3.4%**	**2.9%**	**4.0%**	**4.3%**	**3.6%**	**0.6%**	**8.8%**	**3.0%**	**7.2%**
	10 Years	Current practice	€579.6 B	€287.6 B	€26.1 B	€91.2 B	£149.0 B	$37.5 B	R$784.6 B	¥5.5 T	$1.1 T
		Targeted screening every 3 years + 75% adherence to GDMT	€605.1 B	€297.5 B	€27.4 B	€95.8 B	£157.1 B	$39.2 B	R$855.0 B	¥5.7 T	$1.2 T
		% Change	**4.4%**	**3.5%**	**4.8%**	**5.1%**	**5.5%**	**4.5%**	**9.0%**	**3.7%**	**9.5%**

[a]Currency conversion for the UK from GBP (£) to euro (€) was performed prior to aggregation across European countries using the 2022 annual average from ECB.[26] Values used in conversions were as follows: £1.0 = €1.173.

Abbreviations: B = billion; CKD = chronic kidney disease; ECB = European Central Bank; EUR = Europe; FTE = full-time equivalent; GBP = pound sterling; GDMT = guideline-directed medical therapy; GDP = gross domestic product; M = million; T = trillion; UK = United Kingdoms; US = United States.

Supplementary Table S26. Societal CKD burden over 10 and 25 years following targeted screening every 5 years for high-risk populations and a 75% adherence to GDMT compared to current practice.

Outcome	Time Horizon	Scenario	EUR	Germany	Netherlands	Spain	UK	Australia	Brazil	China	US
**Societal Burden**											
Net Workdays (Total Workdays [Stage 3+] – Missed Workdays Due to Absenteeism in CKD Patients)	25 Years	Current practice	10.5 B	3.4 B	787.9 M	2.0 B	4.4 B	1.2 B	21.7 B	63.5 B	27.5 B
		Targeted screening every 5 years + 75% adherence to GDMT	10.1 B	3.2 B	750.4 M	1.9 B	4.3 B	1.1 B	22.1 B	60.7 B	27.7 B
		% Change	**-3.3%**	**-4.4%**	**-4.8%**	**-3.6%**	**-2.1%**	**-4.8%**	**2.2%**	**-4.3%**	**0.6%**
	10 Years	Current practice	3.7 B	1.1 B	290.7 M	625.7 M	1.6 B	441.0 M	8.9 B	37.4 B	12.0 B
		Targeted screening every 5 years + 75% adherence to GDMT	3.3 B	1.0 B	260.6 M	569.6 M	1.5 B	407.9 M	8.7 B	34.2 B	11.6 B
		% Change	**-8.4%**	**-10.9%**	**-10.3%**	**-9.0%**	**-6.1%**	**-7.5%**	**-2.9%**	**-8.4%**	**-2.7%**

Outcome	Time Horizon	Scenario	EUR	Germany	Netherlands	Spain	UK	Australia	Brazil	China	US
**Societal Burden**											
Net Workdays (Total Workdays [Stage 3+] – Missed Workdays Due to Presenteeism in CKD Patients)	25 Years	Current practice	8.8 B	2.8 B	663.1 M	1.7 B	3.7 B	1.0 B	19.5 B	54.8 B	22.9 B
		Targeted screening every 5 years + 75% adherence to GDMT	8.5 B	2.7 B	627.3 M	1.6 B	3.6 B	1.0 B	19.8 B	52.2 B	22.9 B
		% Change	**-3.8%**	**-4.7%**	**-5.4%**	**-3.9%**	**-2.7%**	**-5.1%**	**1.9%**	**-4.8%**	**0.1%**
	10 Years	Current practice	3.1 B	946.0 M	243.9 M	538.1 M	1.3 B	379.3 M	8.0 B	32.4 B	10.0 B
		Targeted screening every 5 years + 75% adherence to GDMT	2.8 B	837.0 M	216.9 M	487.3 M	1.3 B	348.4 M	7.7 B	29.4 B	9.6 B
		% Change	**-9.0%**	**-11.5%**	**-11.0%**	**-9.5%**	**-6.8%**	**-8.2%**	**-3.3%**	**-9.1%**	**-3.4%**
Net Workdays (Total Workdays [Stage 3+] – Missed Workdays Due to Absenteeism in Caregivers)	25 Years	Current practice	17.2 B	6.2 B	1.5 B	3.8 B	5.7 B	1.9 B	24.8 B	92.2 B	35.1 B
		Targeted screening every 5 years + 75% adherence to GDMT	17.8 B	6.4 B	1.6 B	4.0 B	5.8 B	1.9 B	26.8 B	94.6 B	37.5 B
		% Change	**3.6%**	**2.9%**	**4.7%**	**5.4%**	**3.0%**	**0.4%**	**8.2%**	**2.6%**	**7.0%**
	10 Years	Current practice	5.4 B	2.0 B	457.3 M	1.1 B	1.9 B	581.3 M	8.8 B	30.8 B	12.5 B
		Targeted screening every 5 years + 75% adherence to GDMT	5.6 B	2.1 B	477.9 M	1.1 B	1.9 B	590.7 M	9.3 B	31.4 B	13.3 B
		% Change	**2.8%**	**1.7%**	**4.5%**	**5.3%**	**2.3%**	**1.6%**	**5.6%**	**1.9%**	**6.6%**

Outcome	Time Horizon	Scenario	EUR	Germany	Netherlands	Spain	UK	Australia	Brazil	China	US
**Societal Burden**											
Net GDP Contribution (Total GDP [Stage 3+] – Lost GDP due to Absenteeism/ Presenteeism in Patients and Absenteeism in Caregivers)	25 Years	Current practice	15.0 T	4.9 T	1.1 T	2.7 T	6.2 T	1.7 T	35.1 T	93.7 T	38.7 T
		Targeted screening every 5 years + 75% adherence to GDMT	14.4 T	4.7 T	1.0 T	2.6 T	6.1 T	1.6 T	35.8 T	89.5 T	38.9 T
		% Change	**-3.5%**	**-4.5%**	**-5.0%**	**-3.6%**	**-2.4%**	**-4.8%**	**2.1%**	**-4.5%**	**0.5%**
	10 Years	Current practice	5.2 T	1.7 T	399.0 B	876.2 B	2.3 T	637.0 B	14.4 T	55.3 T	16.8 T
		Targeted screening every 5 years + 75% adherence to GDMT	4.8 T	1.5 T	356.5 B	795.7 B	2.1 T	587.4 B	14.0 T	50.5 T	16.3 T
		% Change	**-8.7%**	**-11.2%**	**-10.7%**	**-9.2%**	**-6.4%**	**-7.8%**	**-3.0%**	**-8.7%**	**-3.0%**
Net FTE (Total FTE [Stage 3+] – Lost FTE due to Absenteeism/ Presenteeism in Patients and Absenteeism in Caregivers)	25 Years	Current practice	127.7 M	48.3 M	11.0 M	24.3 M	44.1 M	12.3 M	134.2 M	609.2 M	279.5 M
		Targeted screening every 5 years + 75% adherence to GDMT	129.3 M	48.7 M	11.2 M	24.9 M	44.4 M	12.1 M	141.6 M	609.1 M	291.7 M
		% Change	**1.2%**	**0.7%**	**1.7%**	**2.7%**	**0.8%**	**-1.5%**	**5.5%**	**0.0%**	**4.4%**
	10 Years	Current practice	41.8 M	16.0 M	3.6 M	7.1 M	15.1 M	4.1 M	51.0 M	259.2 M	108.1 M
		Targeted screening every 5 years + 75% adherence to GDMT	41.3 M	15.7 M	3.5 M	7.2 M	14.9 M	4.0 M	51.7 M	249.9 M	110.6 M
		% Change	**-1.3%**	**-2.3%**	**-0.6%**	**0.6%**	**-1.4%**	**-2.1%**	**1.4%**	**-3.6%**	**2.3%**

Outcome	Time Horizon	Scenario	EUR	Germany	Netherlands	Spain	UK	Australia	Brazil	China	US
**Societal Burden**											
Net Tax Revenue[a] (Total Income [Stage 3+] – Lost Income Due to Absenteeism in CKD Patients and Caregivers * Tax Rate Per Worker)	25 Years	Current practice	€1.8 T	€866.5 B	€79.8 B	€309.4 B	£431.5 B	$111.9 B	R$2.1 T	¥12.4 T	$2.7 T
		Targeted screening every 5 years + 75% adherence to GDMT	€1.8 T	€868.3 B	€80.9 B	€316.1 B	£434.6 B	$110.0 B	R$2.2 T	¥12.4 T	$2.8 T
		% Change	**0.8%**	**0.2%**	**1.4%**	**2.2%**	**0.7%**	**-1.7%**	**5.3%**	**-0.3%**	**4.0%**
	10 Years	Current practice	€579.6 B	€287.6 B	€26.1 B	€91.2 B	£149.0 B	$37.5 B	R$784.6 B	¥5.5 T	$1.1 T
		Targeted screening every 5 years + 75% adherence to GDMT	€567.9 B	€279.1 B	€25.8 B	€91.1 B	£146.6 B	$36.6 B	R$794.8 B	¥5.2 T	$1.1 T
		% Change	**-2.0%**	**-3.0%**	**-1.4%**	**-0.1%**	**-1.6%**	**-2.4%**	**1.3%**	**-3.8%**	**2.0%**

[a]Currency conversion for the UK from GBP (£) to euro (€) was performed prior to aggregation across European countries using the 2022 annual average from ECB.[26] Values used in conversions were as follows: £1.0 = €1.173.

Abbreviations: B = billion; CKD = chronic kidney disease; ECB = European Central Bank; EUR = Europe; FTE = full-time equivalent; GBP = pound sterling; GDMT = guideline-directed medical therapy; GDP = gross domestic product; M = million; T = trillion; UK = United Kingdoms; US = United States.

Supplementary Figure S1. Primary Scenarios - Change in CKD Prevalence (excl. KRT) over 25 years.

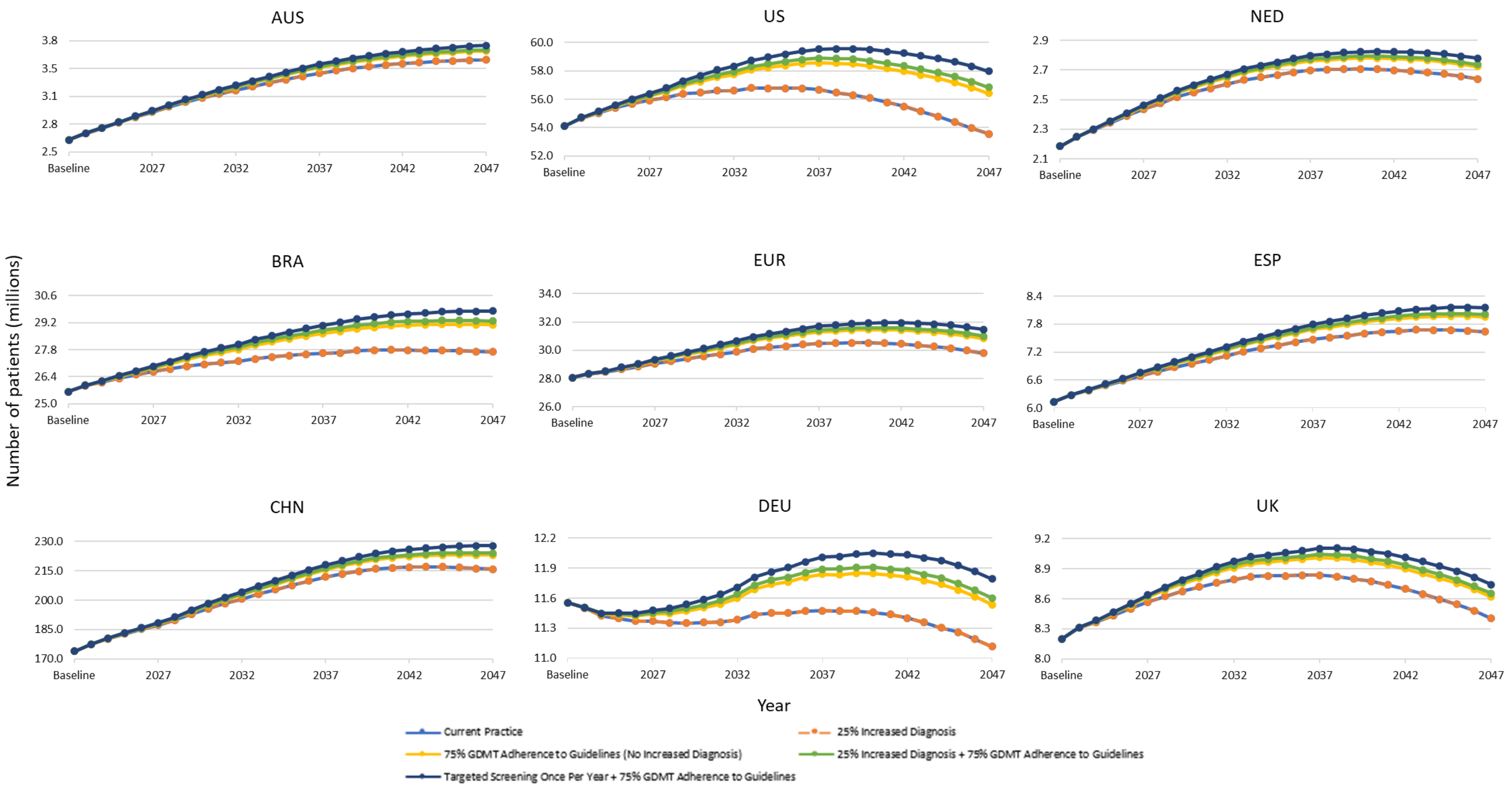

Notes: Line traces for the "current practice" and "25% increased diagnosis" scenarios overlap due to unchanged mortality and disease progression without improved GDMT adherence.
Abbreviations: AUS = Australia; BRA = Brazil; CHN = China; CKD = chronic kidney disease; DEU = Germany; ESP = Spain; EUR = Europe; excl. = excluding; GDMT = guideline-directed medical therapy; KRT = kidney replacement therapy; NED = Netherlands; UK = United Kingdom; US = United States.

Supplementary Figure S2. Primary Scenarios - Change in Freshwater Consumption due to KRT over 25 years.

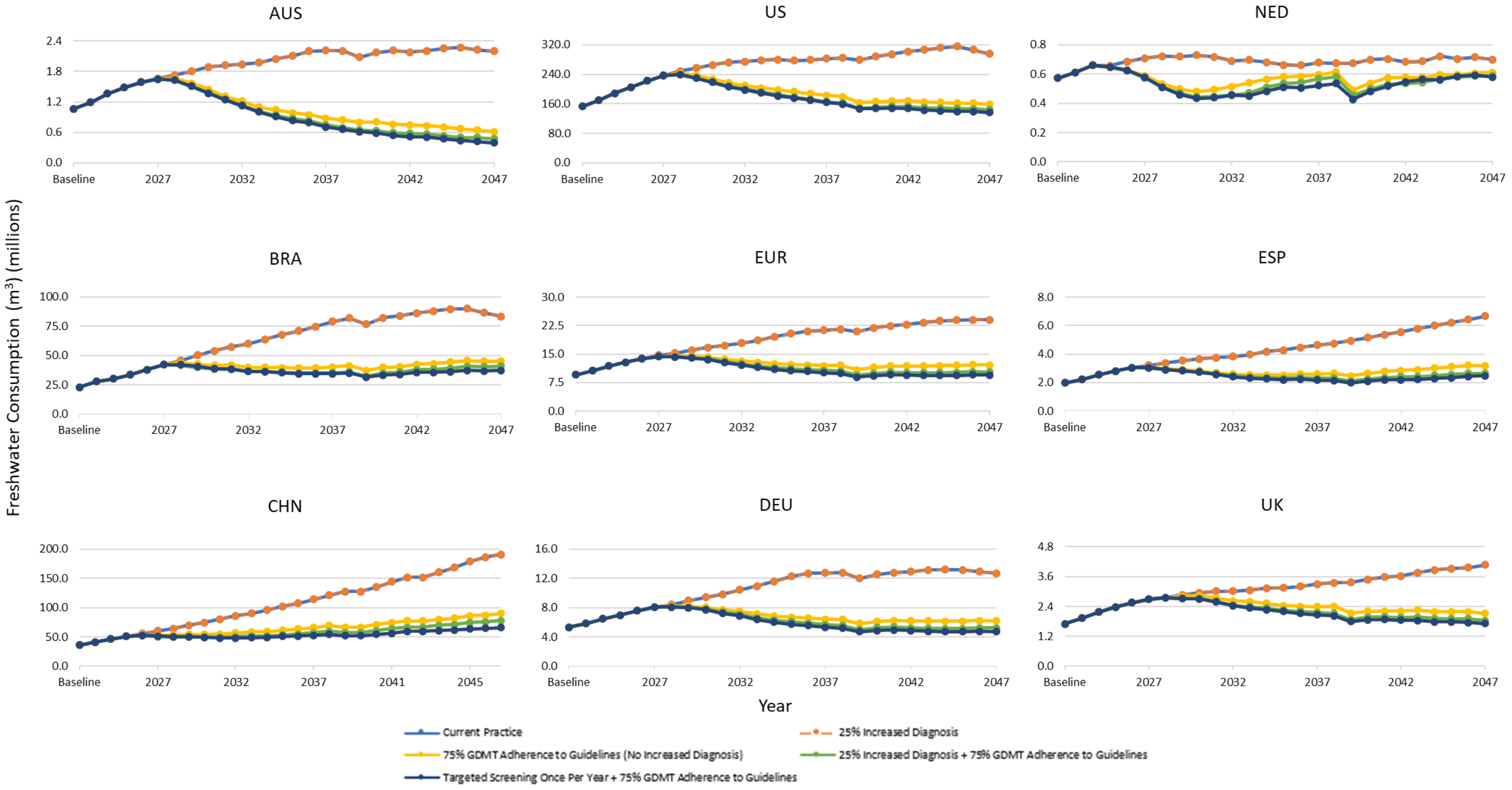

Notes: Line traces for the "current practice" and "25% increased diagnosis" scenarios overlap due to unchanged mortality and disease progression without improved GDMT adherence.

Abbreviations: AUS = Australia; BRA = Brazil; CHN = China; DEU = Germany; ESP = Spain; EUR = Europe; GDMT = guideline-directed medical therapy; KRT = kidney replacement therapy; NED = Netherlands; UK = United Kingdom; US = United States.

Supplementary Figure S3. Primary Scenarios - Change in Fossil Fuel Depletion due to KRT over 25 years.

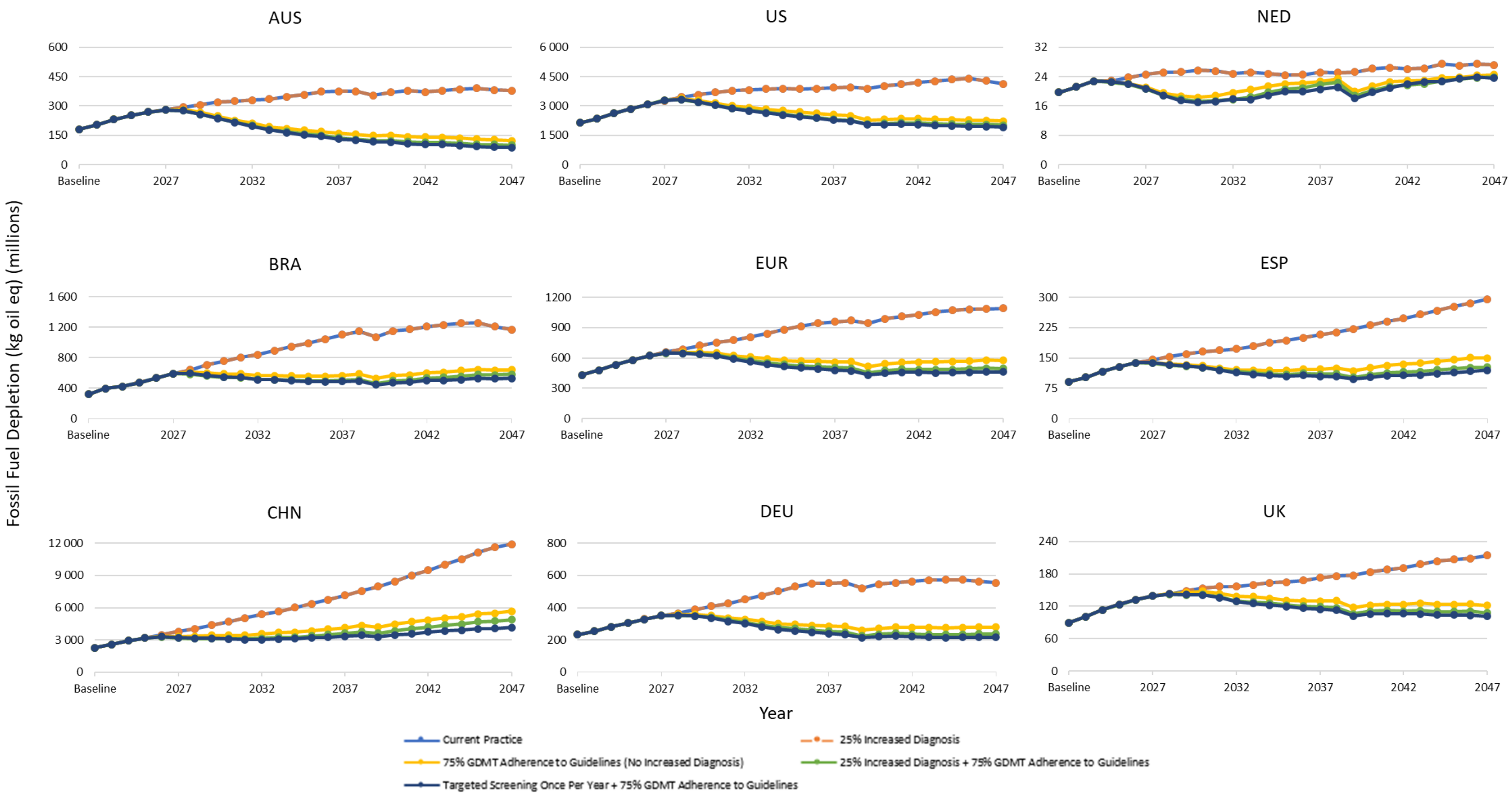

Notes: Line traces for the "current practice" and "25% increased diagnosis" scenarios overlap due to unchanged mortality and disease progression without improved GDMT adherence.
Abbreviations: AUS = Australia; BRA = Brazil; CHN = China; DEU = Germany; ESP = Spain; eq = equivalents; EUR = Europe; GDMT = guideline-directed medical therapy; KRT = kidney replacement therapy; NED = Netherlands; UK = United Kingdom; US = United States.

Supplementary Figure S4. Primary Scenarios - Change in non-KRT CKD Costa over 25 years.

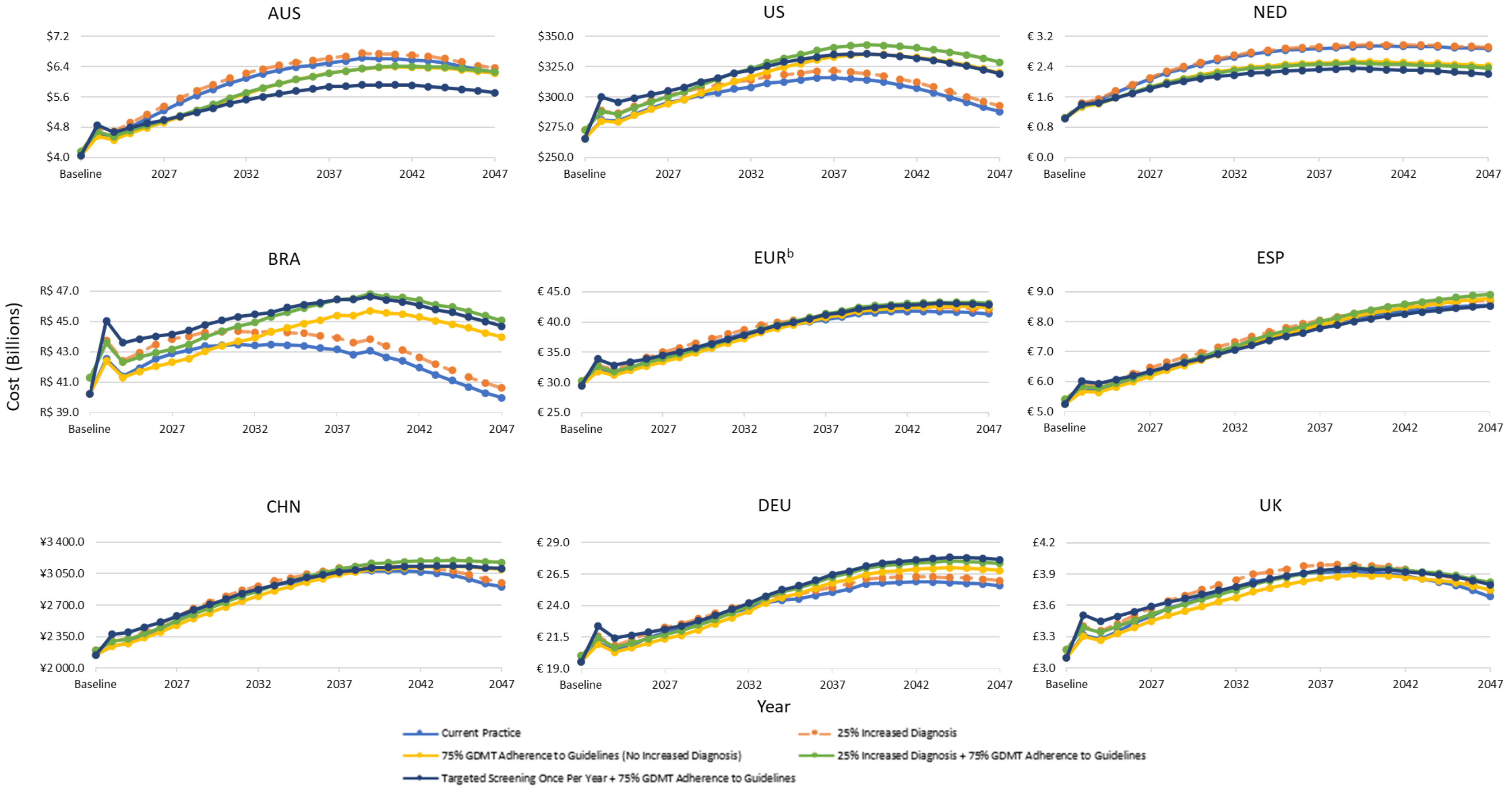

[a]Does not include costs associated with CKD treatment or screening.

[b]Currency conversion for the UK from GBP (£) to euro (€) was performed prior to aggregation across European countries using the 2022 annual average from ECB.[30] Values used in conversions were as follows: £1.0 = €1.173.

Abbreviations: AUS = Australia; BRA = Brazil; CHN = China; CKD = chronic kidney disease; DEU = Germany; ECB = European Central Bank; ESP = Spain; EUR = Europe; GBP = pound sterling; GDMT = guideline-directed medical therapy; KRT = kidney replacement therapy; NED = Netherlands; UK = United Kingdom; US = United States.

Supplementary Figure S5. Primary Scenarios - Change in Total Cost[a] (Incl. non-KRT CKD Cost, KRT Cost, Treatment Cost) over 25 years.

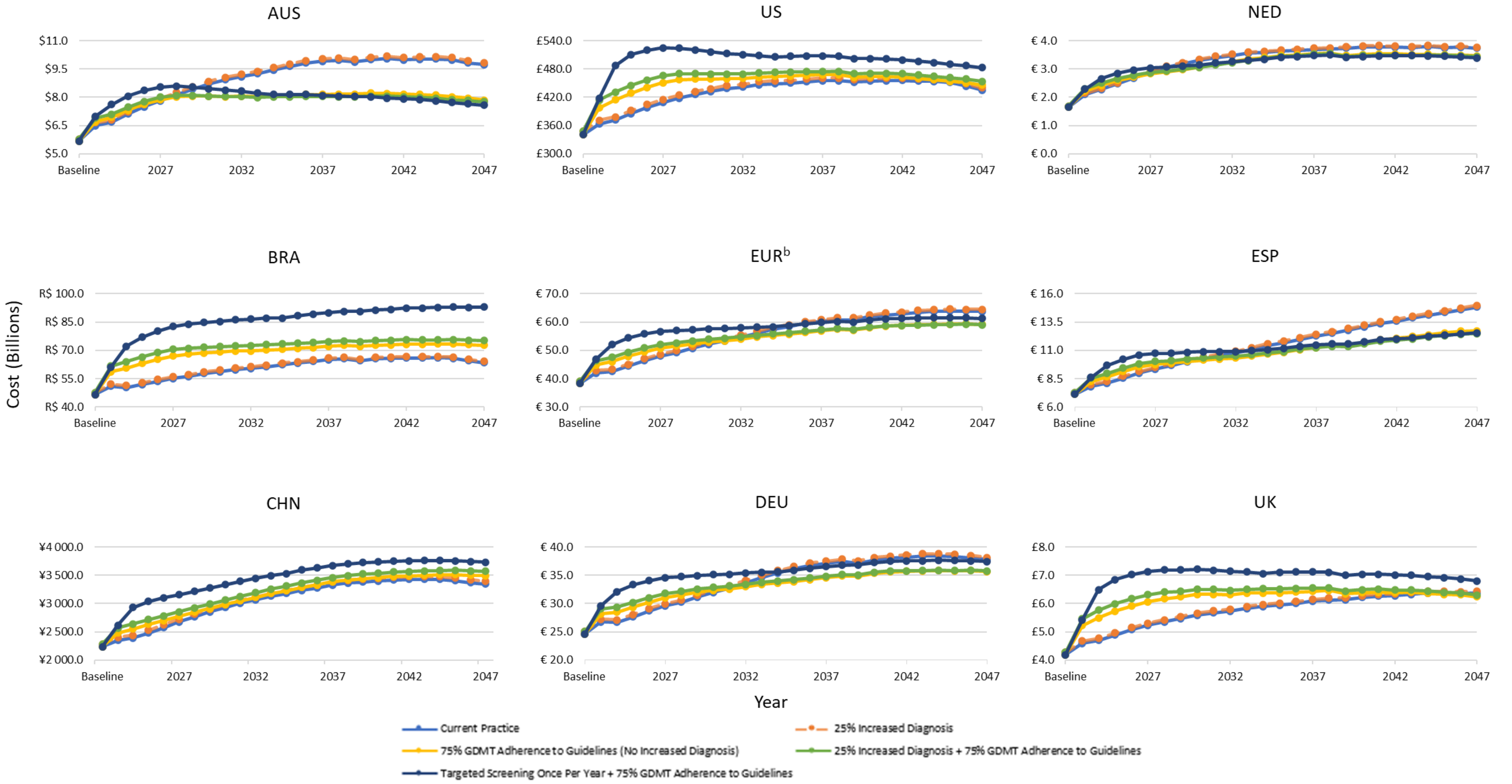

[a]Does not include costs associated with CKD screening.

[b]Currency conversion for the UK from GBP (£) to euro (€) was performed prior to aggregation across European countries using the 2022 annual average from ECB.[30] Values used in conversions were as follows: £1.0 = €1.173.

Abbreviations: AUS = Australia; BRA = Brazil; CHN = China; CKD = chronic kidney disease; DEU = Germany; ECB = European Central Bank; ESP = Spain; EUR = Europe; GBP = pound sterling; GDMT = guideline-directed medical therapy; incl. = including; KRT = kidney replacement therapy; NED = Netherlands; UK = United Kingdom; US = United States.

Supplementary Figure S6. Primary Scenarios - Change in Freshwater Consumption due to all CKD over 25 years.

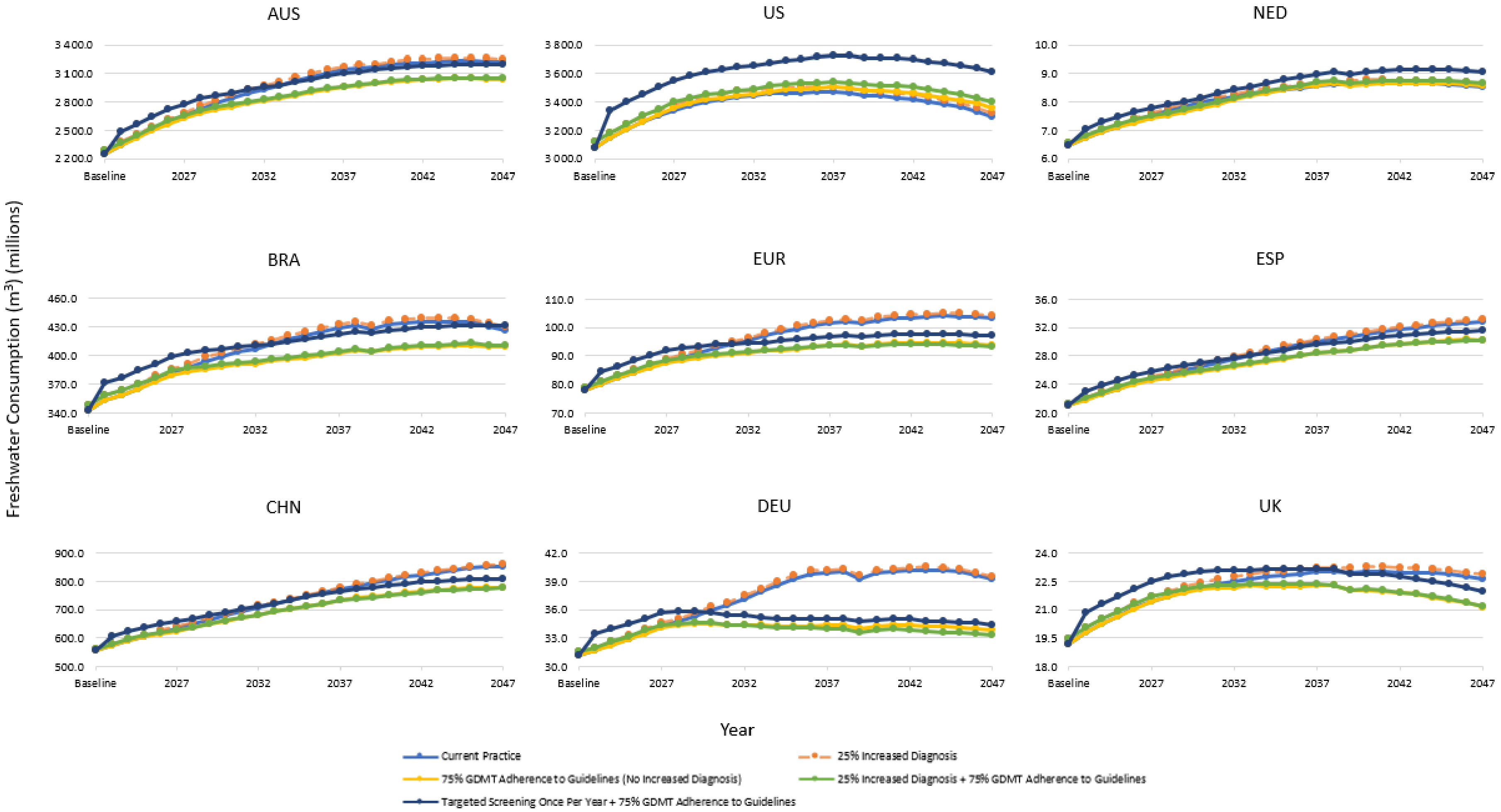

Abbreviations: AUS = Australia; BRA = Brazil; CHN = China; CKD = chronic kidney disease; DEU = Germany; ESP = Spain; EUR = Europe; GDMT = guideline-directed medical therapy; NED = Netherlands; UK = United Kingdom; US = United States.

Supplementary Figure S7. Primary Scenarios - Change in Fossil Fuel Depletion due to all CKD over 25 years.

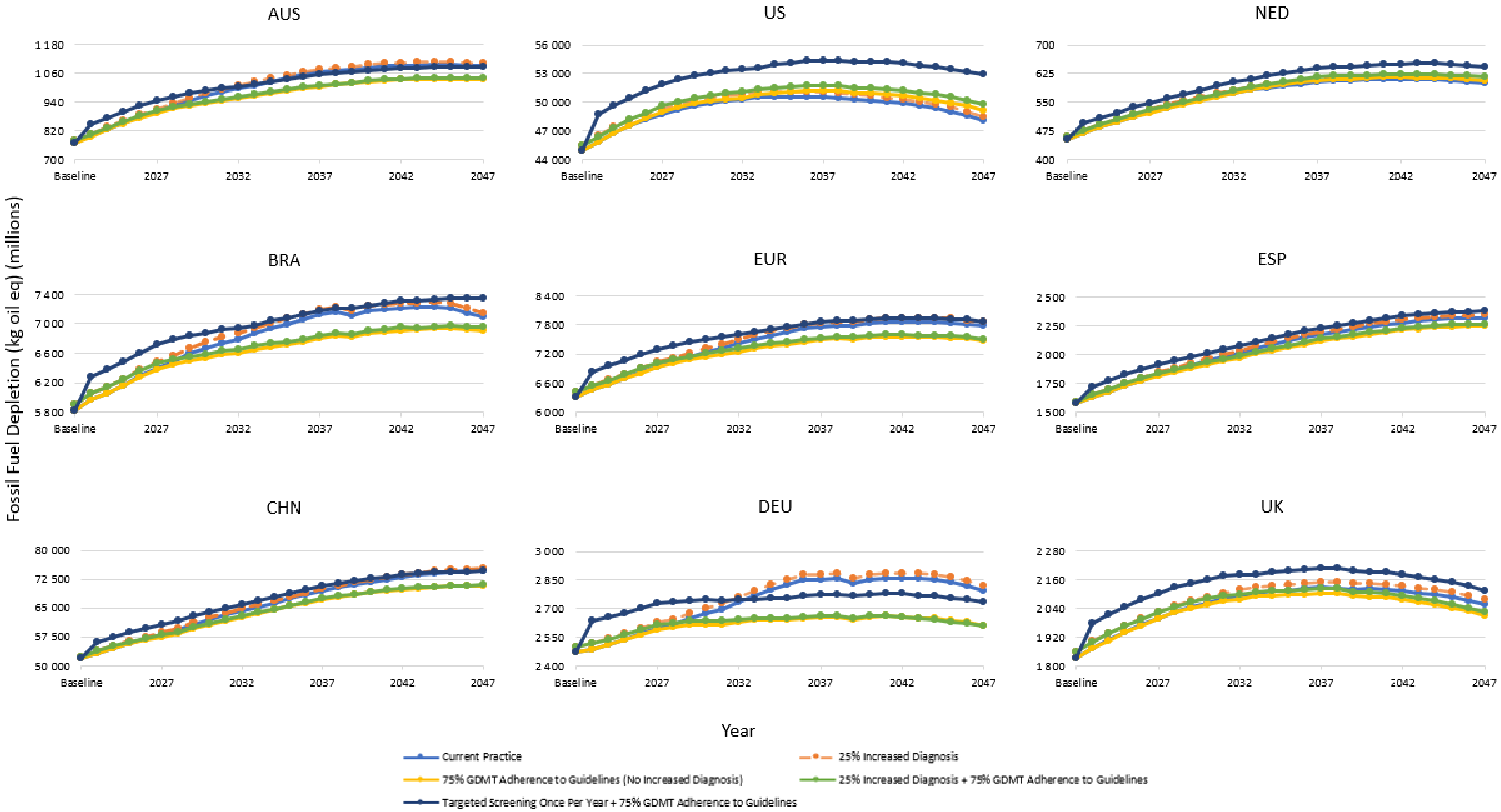

Abbreviations: AUS = Australia; BRA = Brazil; CHN = China; CKD = chronic kidney disease; DEU = Germany; eq = equivalent; ESP = Spain; EUR = Europe; GDMT = guideline-directed medical therapy; NED = Netherlands; UK = United Kingdom; US = United States.

Supplementary Figure S8. Primary Scenarios - Change in Carbon Footprint due to all CKD over 25 years.

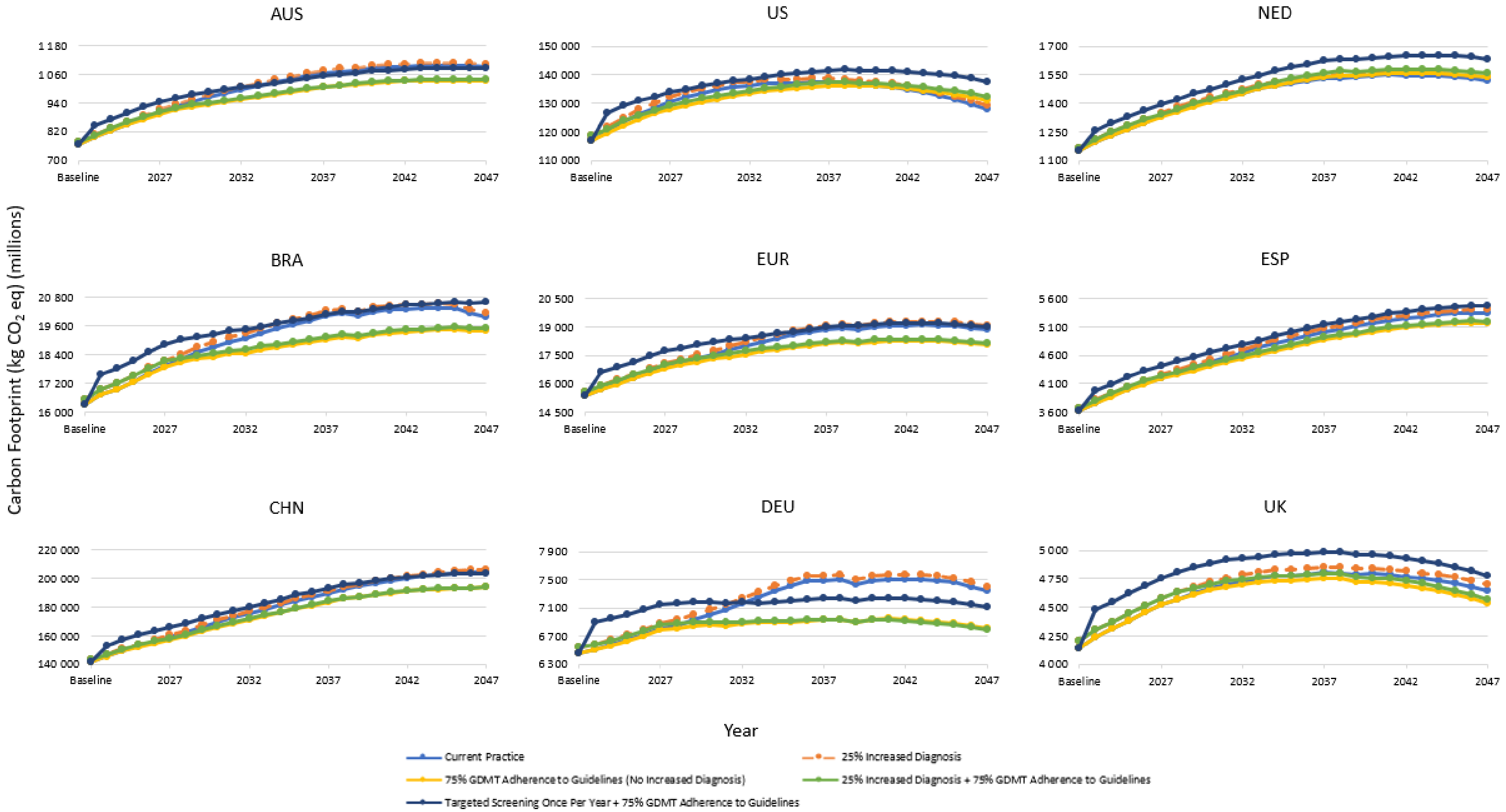

Abbreviations: AUS = Australia; BRA = Brazil; CHN = China; CKD = chronic kidney disease; DEU = Germany; eq = equivalent; ESP = Spain; EUR = Europe; GDMT = guideline-directed medical therapy; NED = Netherlands; UK = United Kingdom; US = United States.

Supplementary Figure S9. Sensitivity Scenarios - Change in CKD Patients requiring dialysis over 25 years.

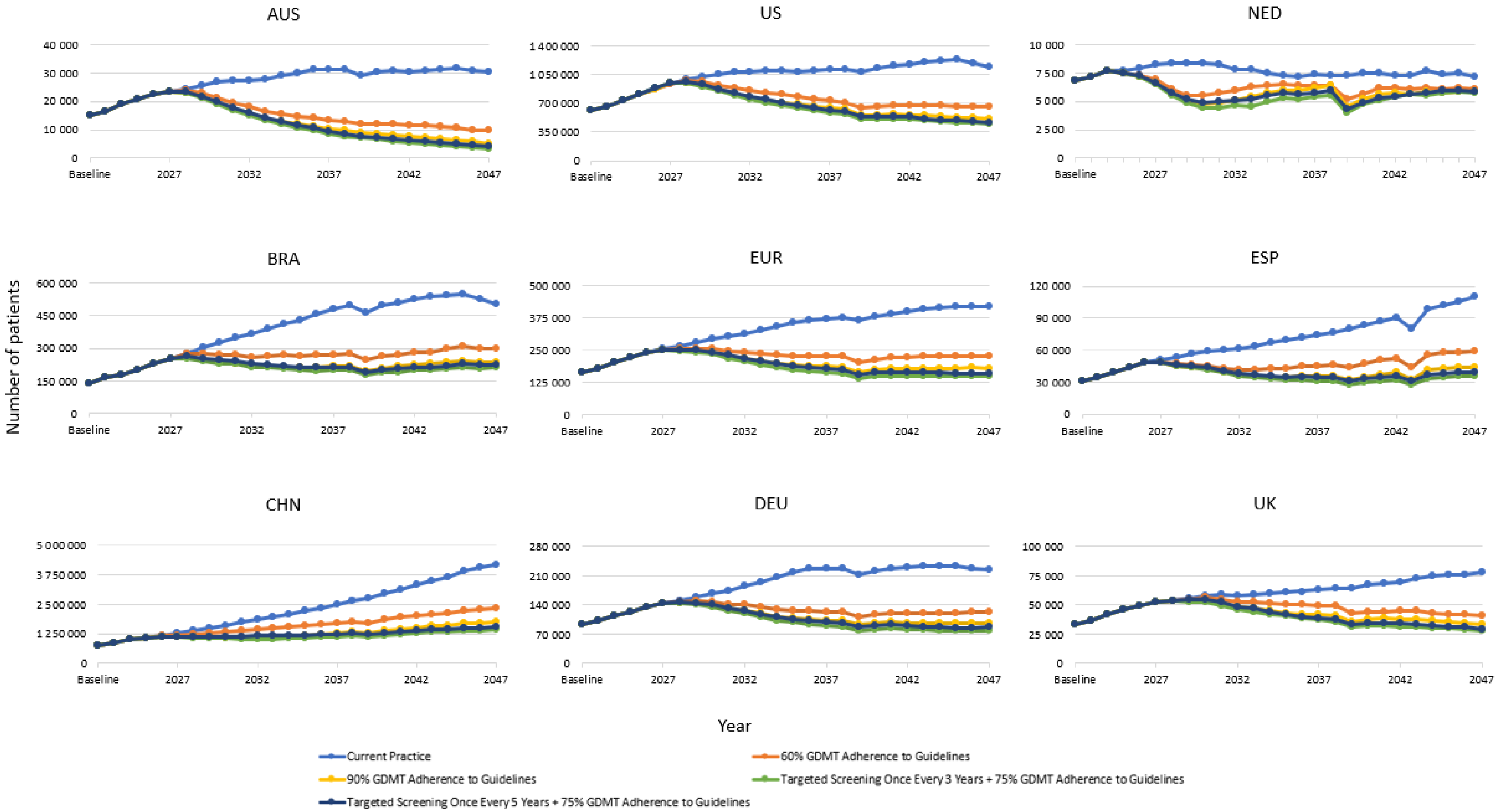

Abbreviations: AUS = Australia; BRA = Brazil; CHN = China; CKD = chronic kidney disease; DEU = Germany; ESP = Spain; EUR = Europe; GDMT = guideline-directed medical therapy; NED = Netherlands; UK = United Kingdom; US = United States.

Supplementary Figure S10. Sensitivity Scenarios - Change in Cardiovascular Events over 25 years.

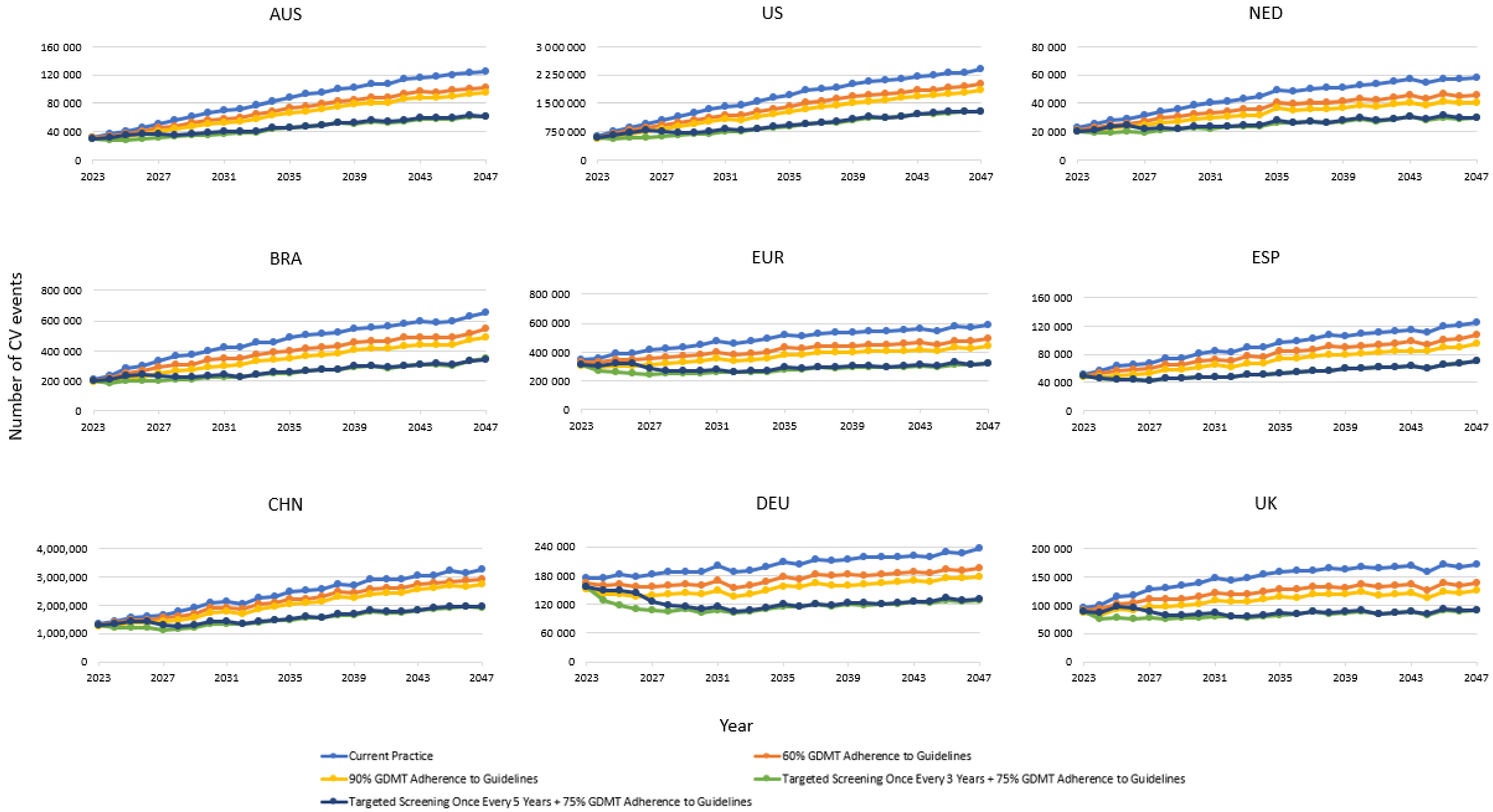

Abbreviations: AUS = Australia; BRA = Brazil; CHN = China; CV = cardiovascular; DEU = Germany; ESP = Spain; EUR = Europe; GDMT = guideline-directed medical therapy; NED = Netherlands; UK = United Kingdom; US = United States.

Supplementary Figure S11. Sensitivity Scenarios - Change in Mortality over 25 years.

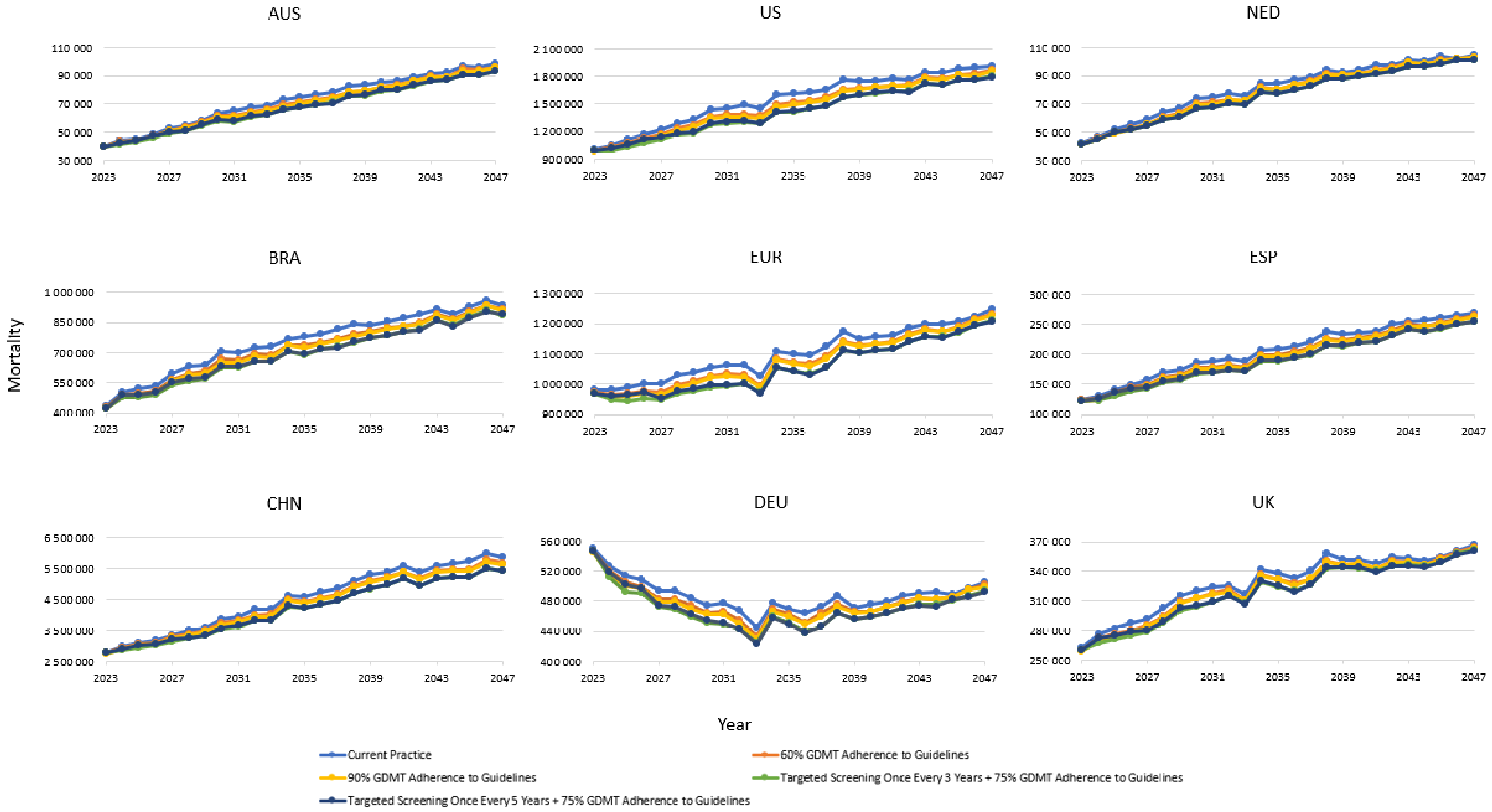

Abbreviations: AUS = Australia; BRA = Brazil; CHN = China; DEU = Germany; ESP = Spain; EUR = Europe; GDMT = guideline-directed medical therapy; NED = Netherlands; UK = United Kingdom; US = United States.

Supplementary Figure S12. Sensitivity Scenarios - Change in CKD Prevalence (excl. KRT) over 25 years.

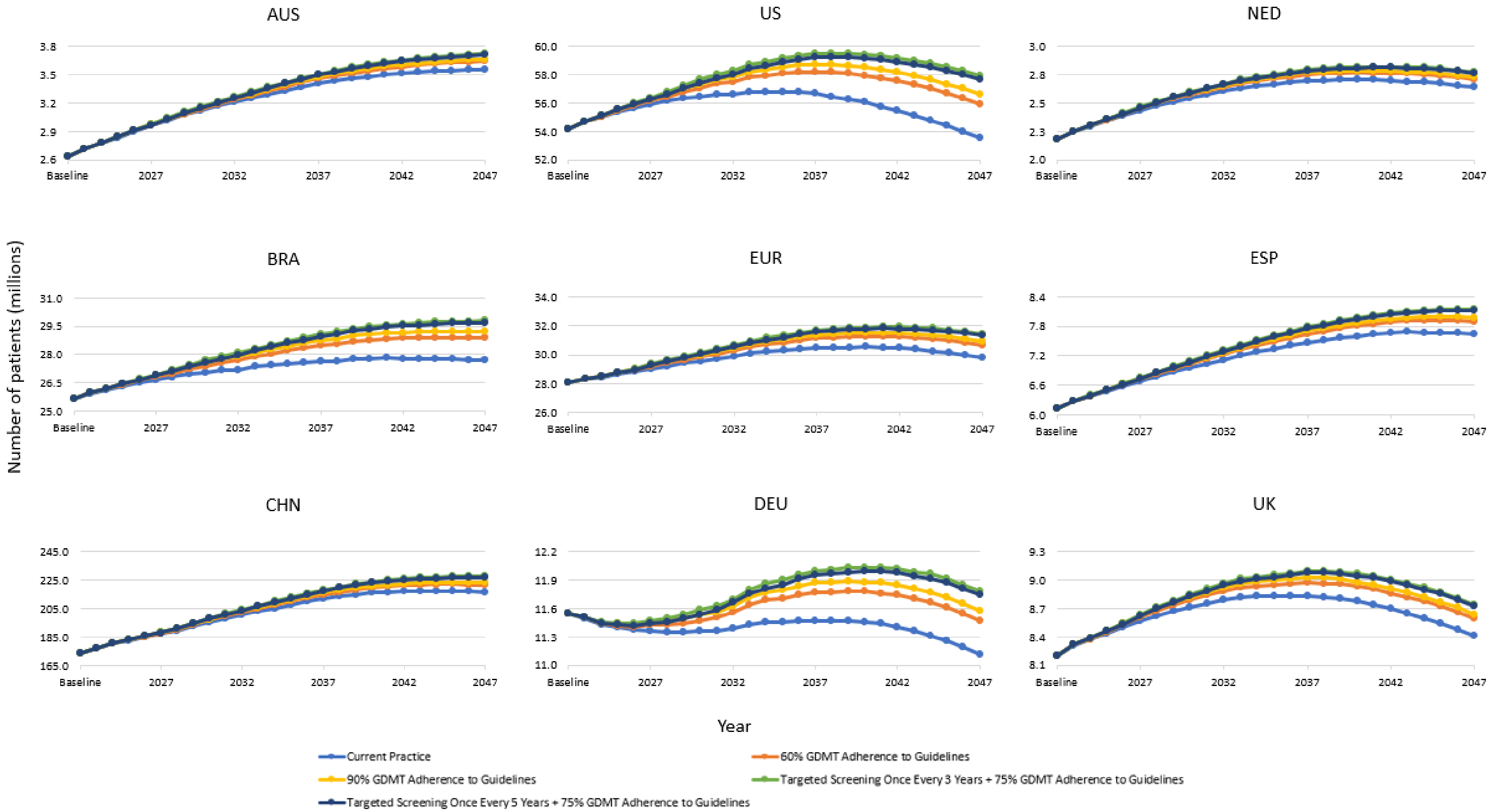

Abbreviations: AUS = Australia; BRA = Brazil; CHN = China; CKD = chronic kidney disease; DEU = Germany; ESP = Spain; EUR = Europe; excl. = excluding; GDMT = guideline-directed medical therapy; KRT = kidney replacement therapy; NED = Netherlands; UK = United Kingdom; US = United States.

Supplementary Figure S13. Sensitivity Scenarios - Change in non-KRT CKD Cost[a] over 25 years.

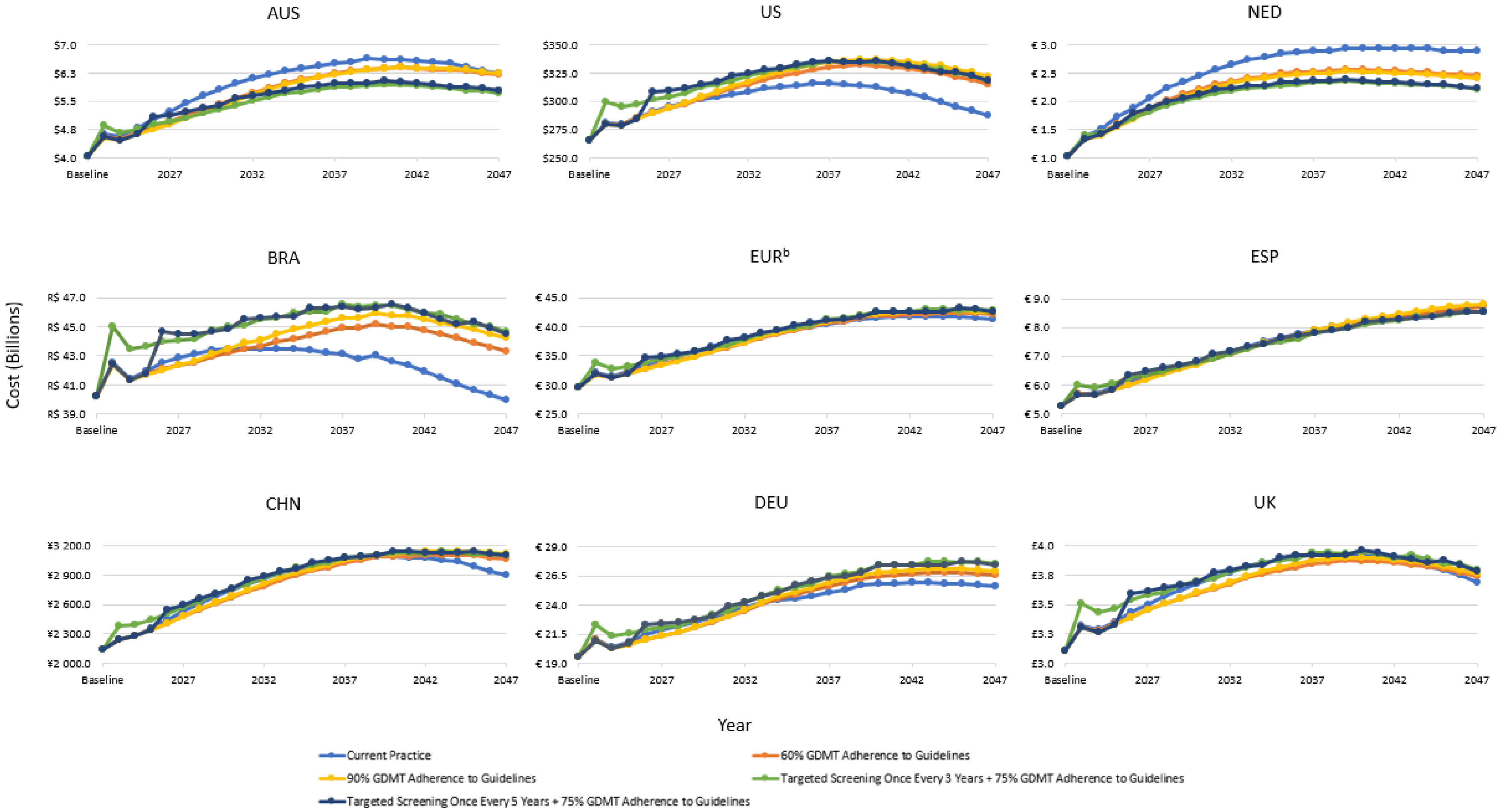

[a]Does not include costs associated with CKD treatment or screening.
[b]Currency conversion for the UK from GBP (£) to euro (€) was performed prior to aggregation across European countries using the 2022 annual average from ECB.[26] Values used in conversions were as follows: £1.0 = €1.173.
Abbreviations: AUS = Australia; BRA = Brazil; CHN = China; CKD = chronic kidney disease; DEU = Germany; ECB = European Central Bank; ESP = Spain; EUR = Europe; GBP = pound sterling; GDMT = guideline-directed medical therapy; KRT = kidney replacement therapy; NED = Netherlands; UK = United Kingdom; US = United States.

Supplementary Figure S14. Sensitivity Scenarios - Change in KRT Cost[a] over 25 years.

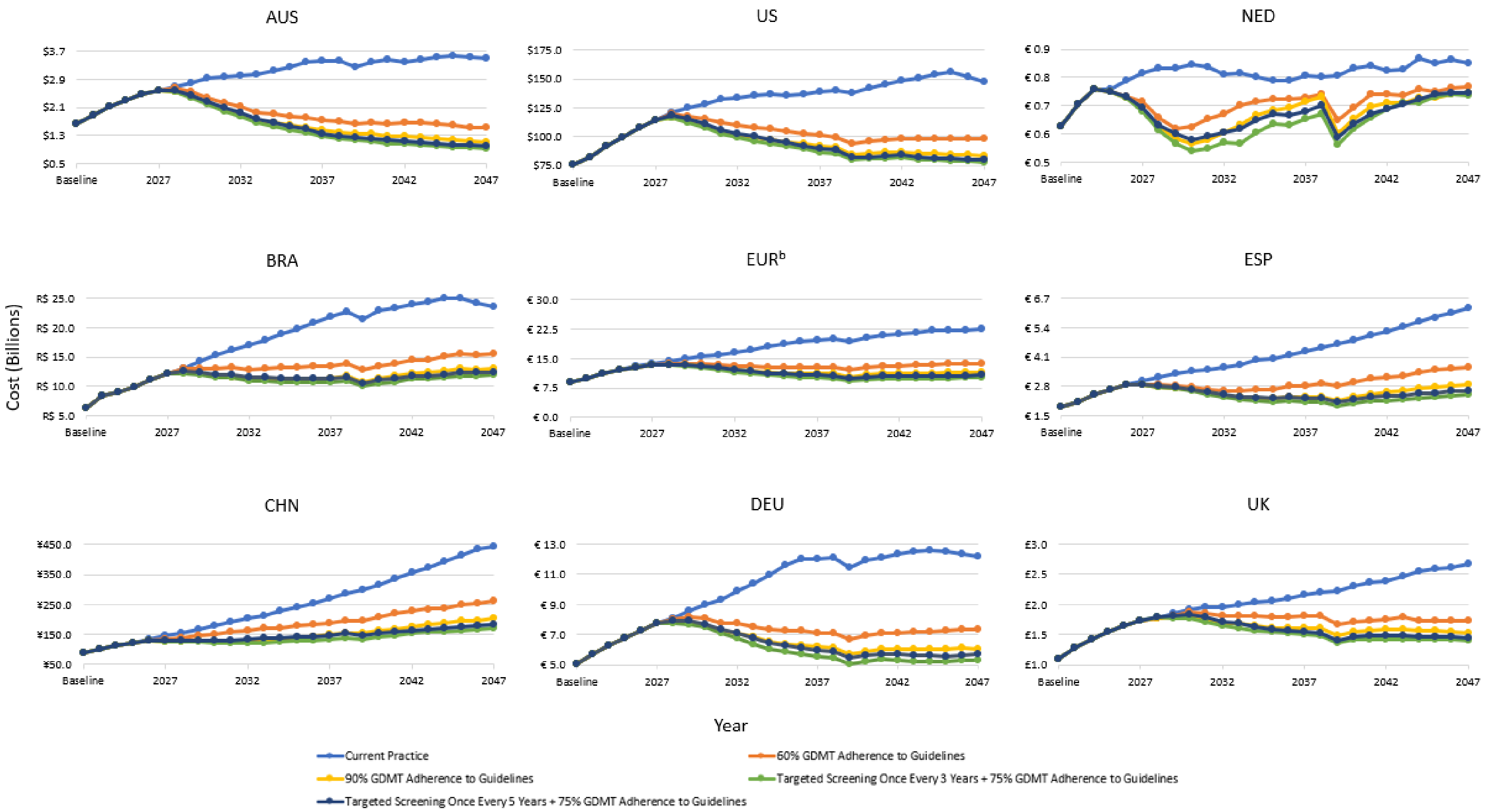

[a]Does not include costs associated with CKD treatment or screening.
[b]Currency conversion for the UK from GBP (£) to euro (€) was performed prior to aggregation across European countries using the 2022 annual average from ECB.[26] Values used in conversions were as follows: £1.0 = €1.173.
Abbreviations: AUS = Australia; BRA = Brazil; CHN = China; CKD = chronic kidney disease; DEU = Germany; ECB = European Central Bank; ESP = Spain; EUR = Europe; GBP = pound sterling; GDMT = guideline-directed medical therapy; KRT = kidney replacement therapy; NED = Netherlands; UK = United Kingdom; US = United States.

Supplementary Figure S15. Sensitivity Scenarios - Change in Total Cost[a] over 25 years.

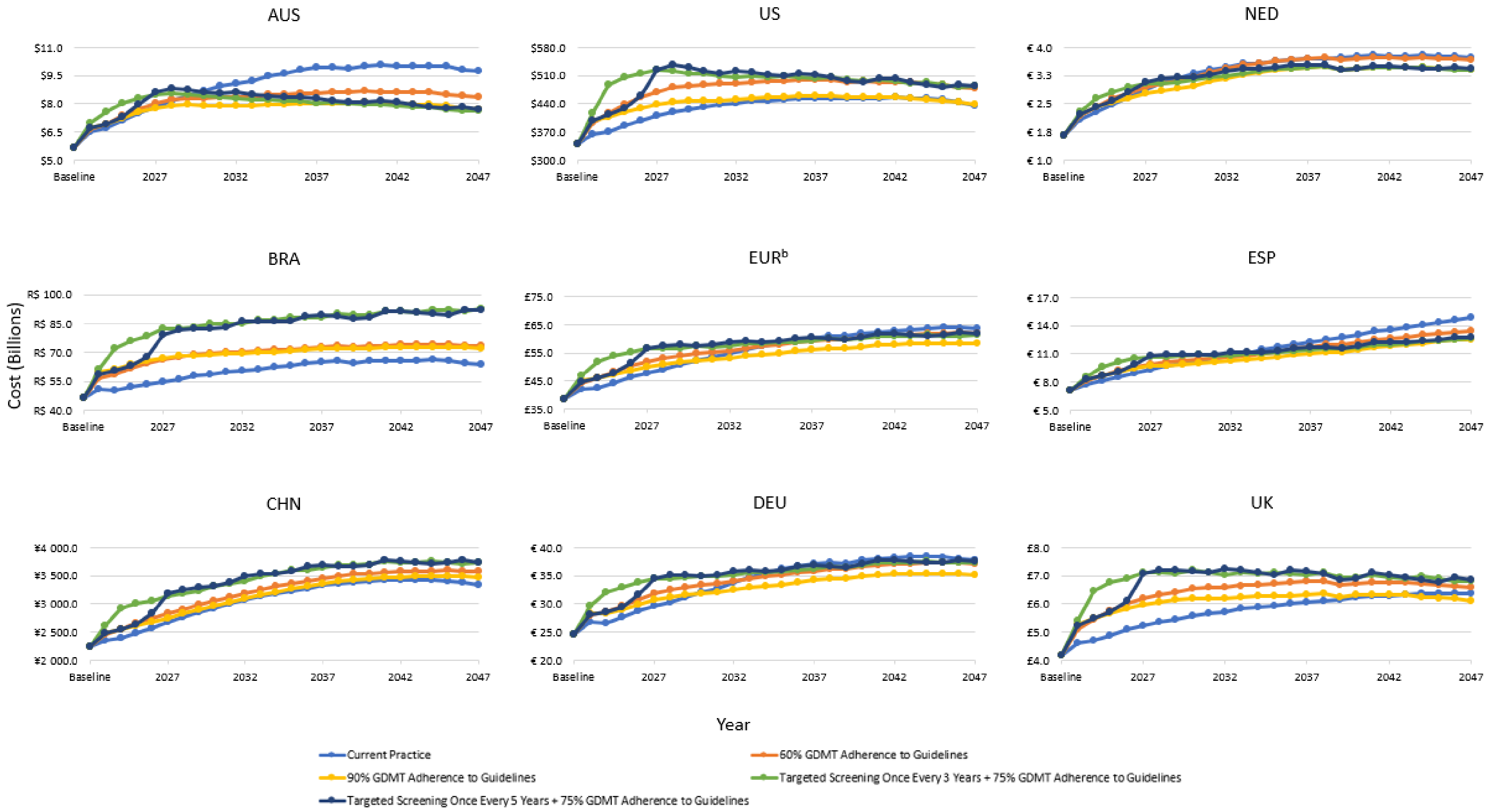

[a]Does not include costs associated with CKD screening.
[b]Currency conversion for the UK from GBP (£) to euro (€) was performed prior to aggregation across European countries using the 2022 annual average from ECB.[26] Values used in conversions were as follows: £1.0 = €1.173.
Abbreviations: AUS = Australia; BRA = Brazil; CHN = China; CKD = chronic kidney disease; DEU = Germany; ECB = European Central Bank; ESP = Spain; EUR = Europe; GBP = pound sterling; GDMT = guideline-directed medical therapy; NED = Netherlands; UK = United Kingdom; US = United States.

Supplementary Figure S16. Sensitivity Scenarios - Change in Freshwater Consumption due to all CKD over 25 years.

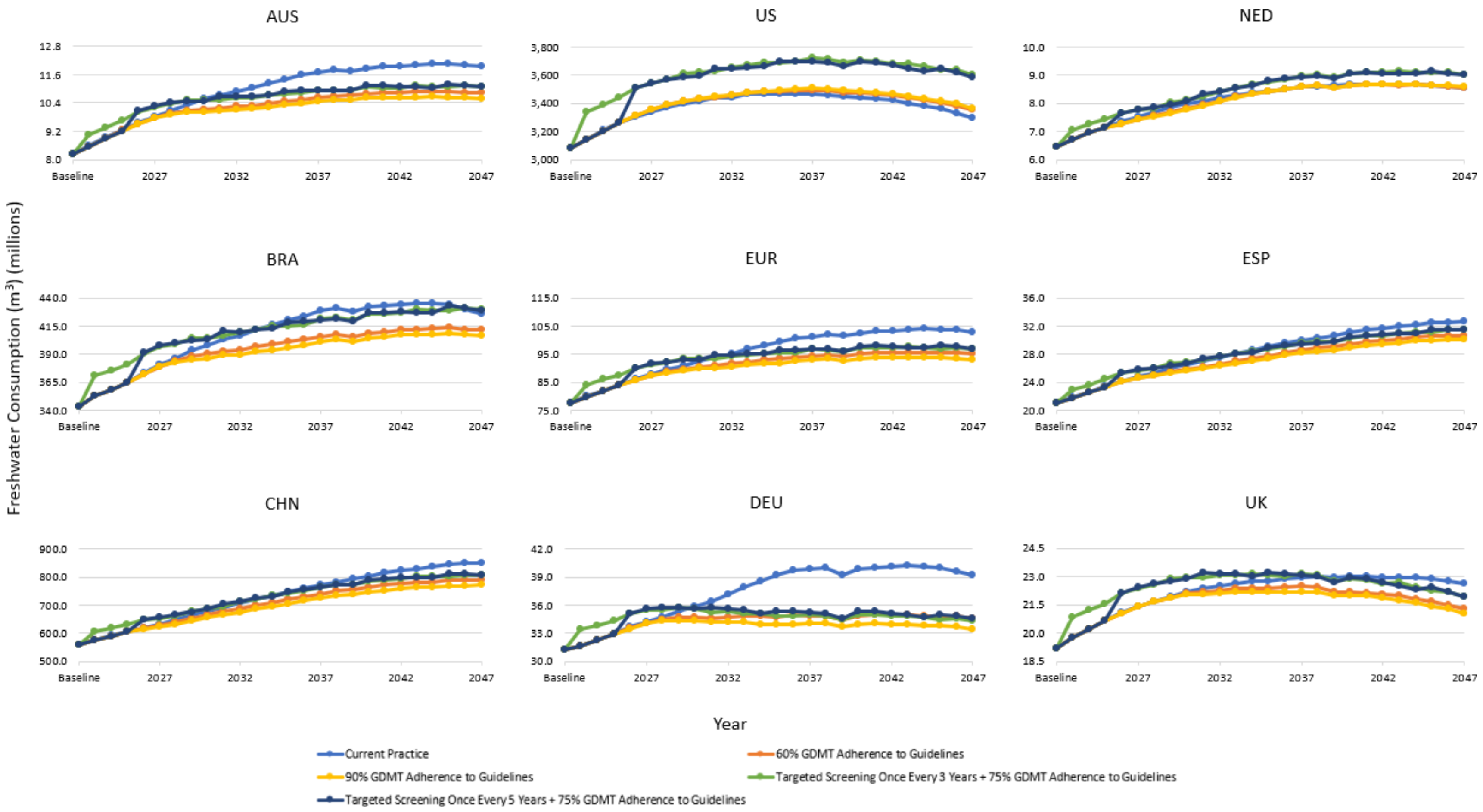

Abbreviations: AUS = Australia; BRA = Brazil; CHN = China; CKD = chronic kidney disease; DEU = Germany; ESP = Spain; EUR = Europe; GDMT = guideline-directed medical therapy; NED = Netherlands; UK = United Kingdom; US = United States.

Supplementary Figure S17. Sensitivity Scenarios - Change in Fossil Fuel Depletion due to all CKD over 25 years.

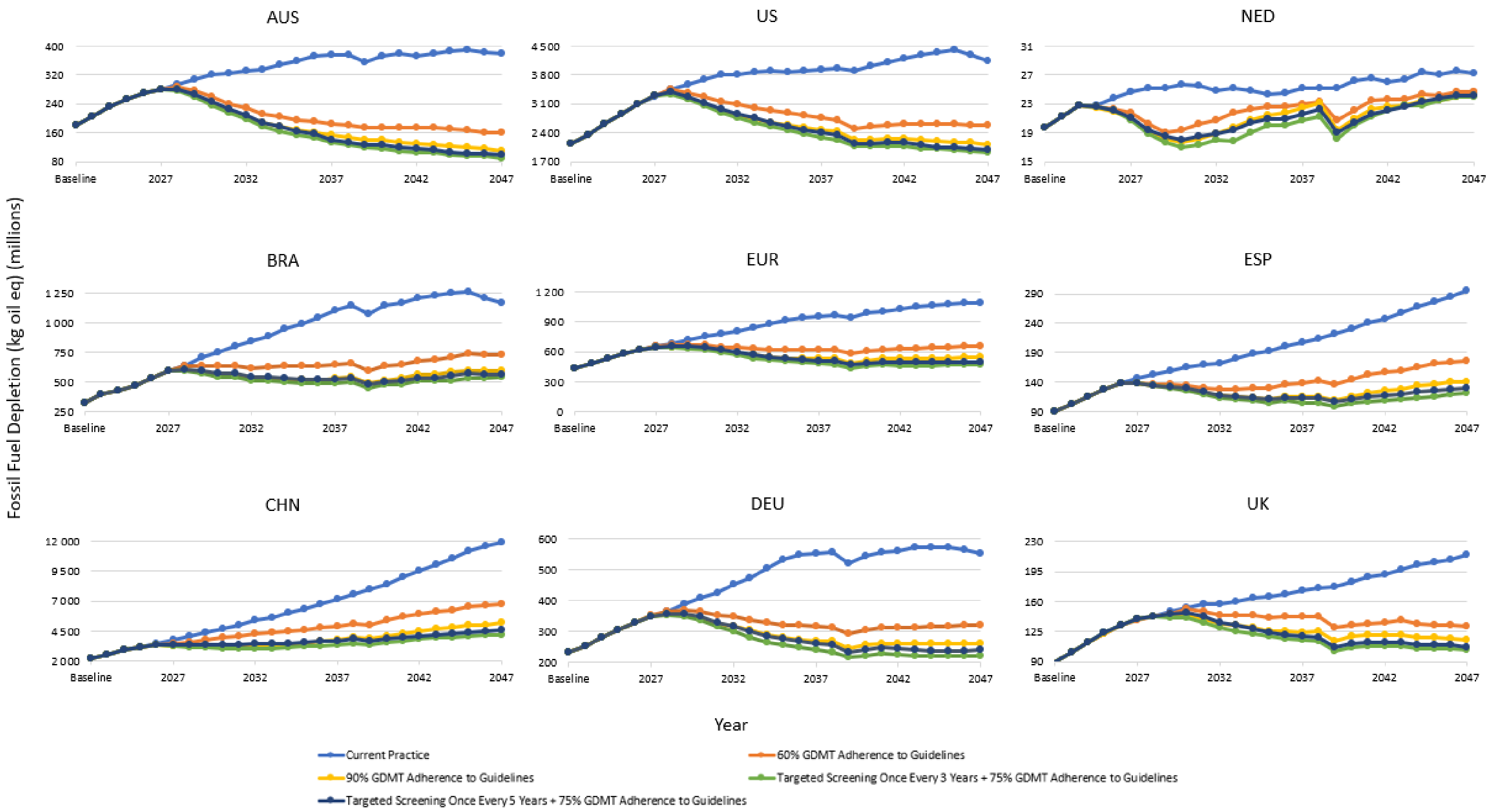

Abbreviations: AUS = Australia; BRA = Brazil; CHN = China; CKD = chronic kidney disease; DEU = Germany; eq = equivalent; ESP = Spain; EUR = Europe; GDMT = guideline-directed medical therapy; NED = Netherlands; UK = United Kingdom; US = United States.

Supplementary Figure S18. Sensitivity Scenarios - Change in Carbon Footprint due to all CKD over 25 years.

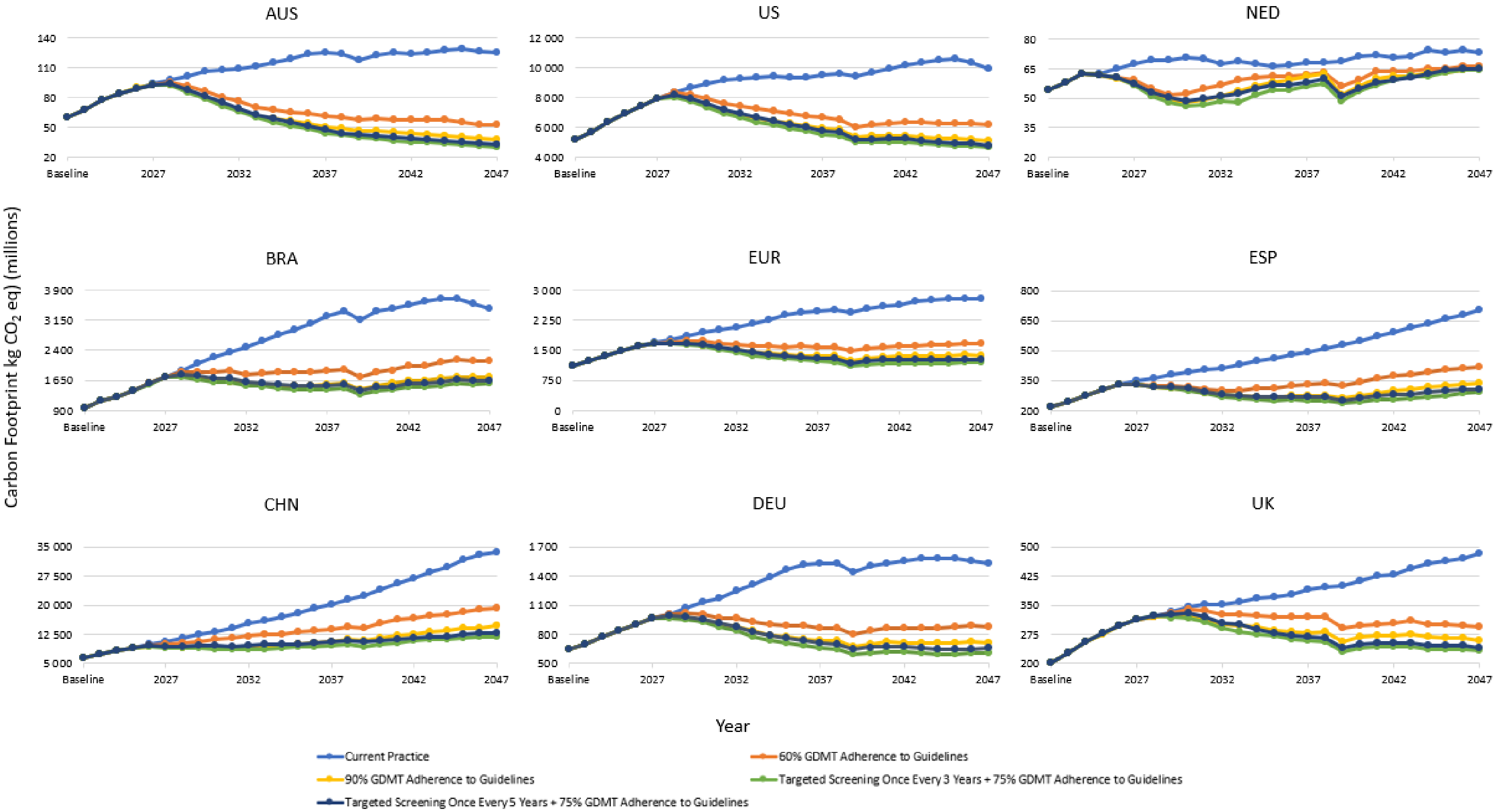

Abbreviations: AUS = Australia; BRA = Brazil; CHN = China; CKD = chronic kidney disease; DEU = Germany; eq = equivalent; ESP = Spain; EUR = Europe; GDMT = guideline-directed medical therapy; NED = Netherlands; UK = United Kingdom; US = United States.

Supplementary Figure S19. Sensitivity Scenarios - Change in Freshwater Consumption due to KRT over 25 years.

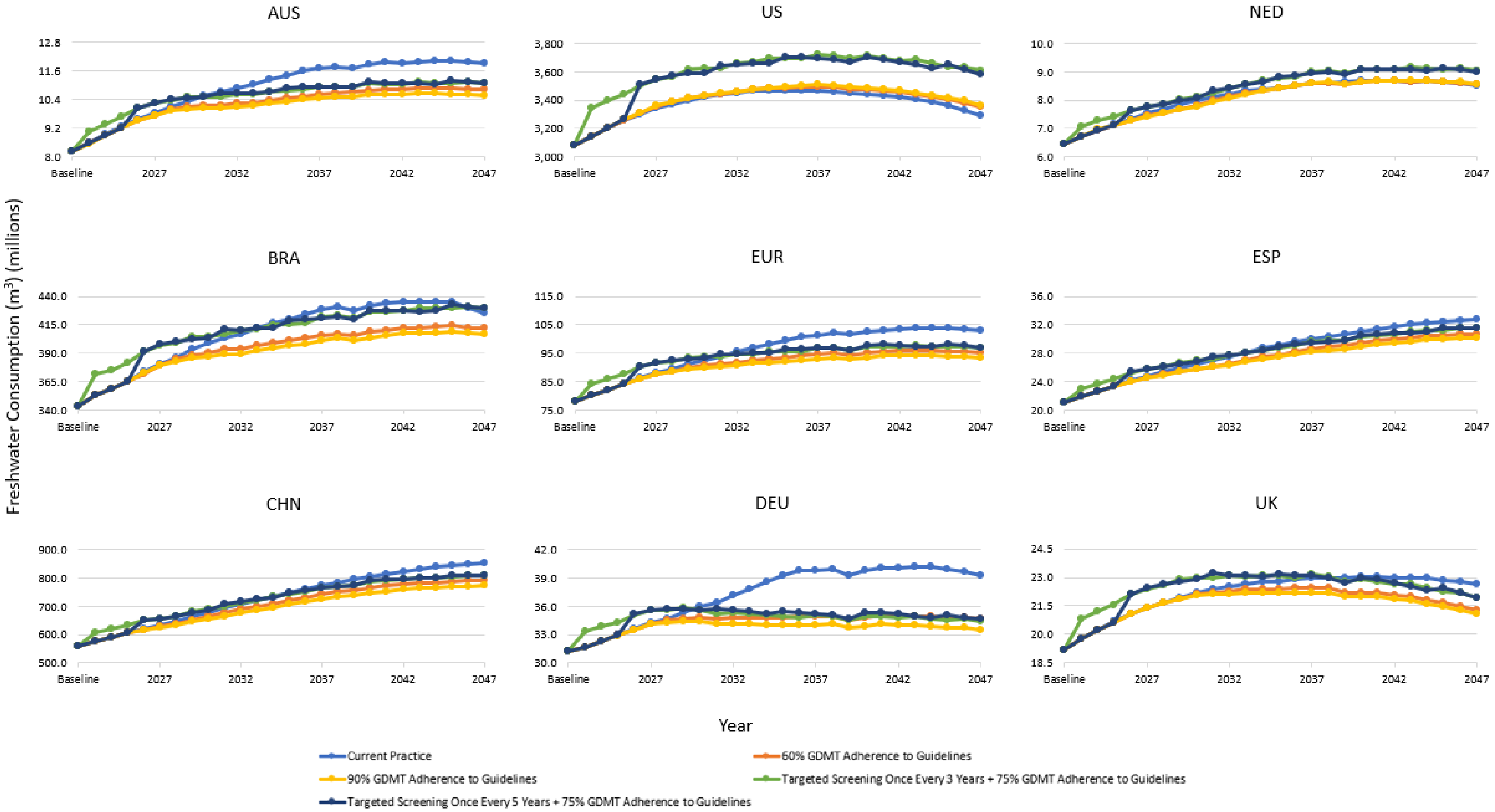

Abbreviations: AUS = Australia; BRA = Brazil; CHN = China; DEU = Germany; ESP = Spain; EUR = Europe; GDMT = guideline-directed medical therapy; KRT = kidney replacement therapy; NED = Netherlands; UK = United Kingdom; US = United States.

Supplementary Figure S20. Sensitivity Scenarios - Change in Fossil Fuel Depletion due to KRT over 25 years.

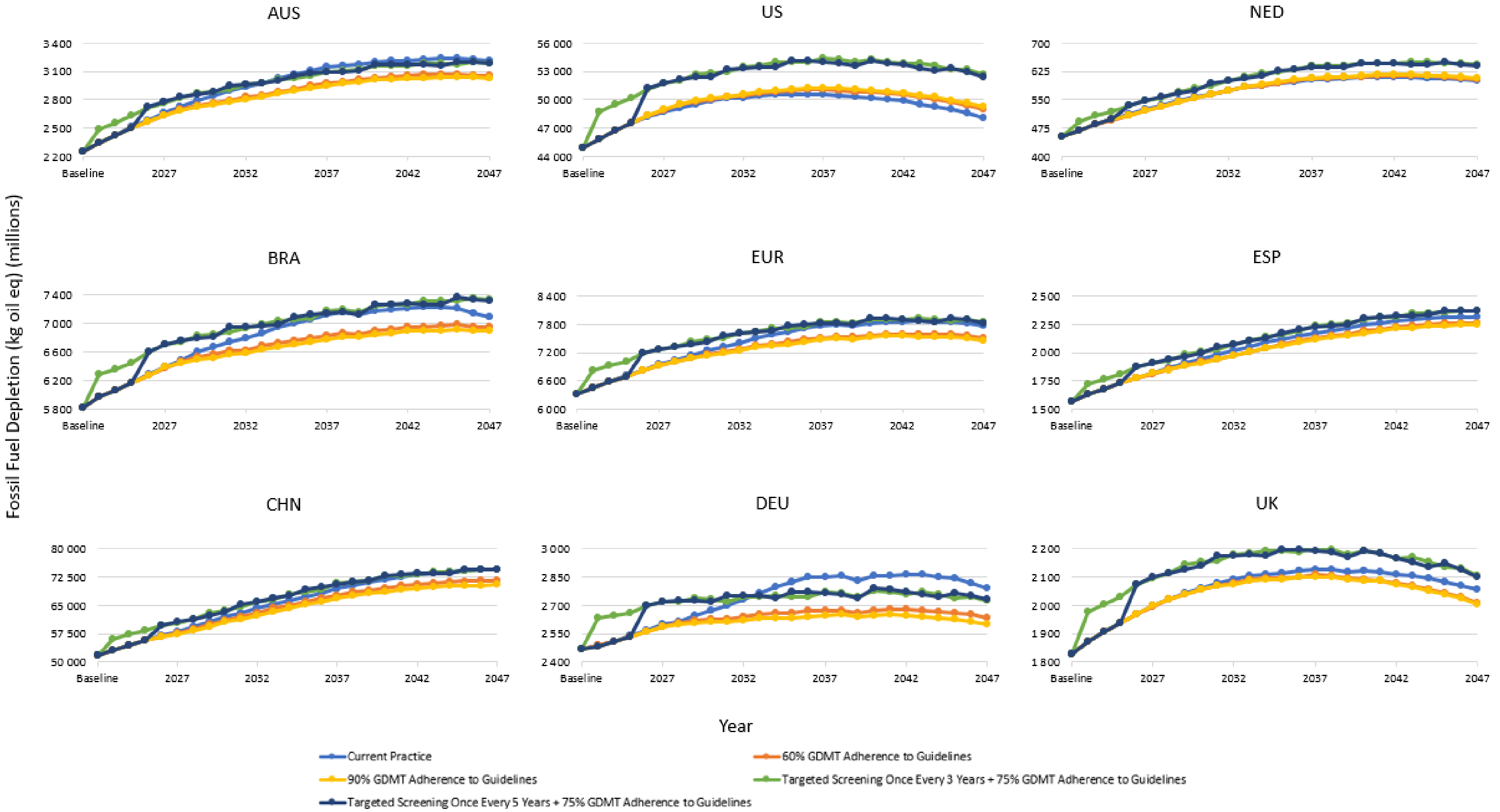

Abbreviations: AUS = Australia; BRA = Brazil; CHN = China; DEU = Germany; eq = equivalent; ESP = Spain; EUR = Europe; GDMT = guideline-directed medical therapy; KRT = kidney replacement therapy; NED = Netherlands; UK = United Kingdom; US = United States.

Supplementary Figure S21. Sensitivity Scenarios - Change in Carbon Footprint due to KRT over 25 years.

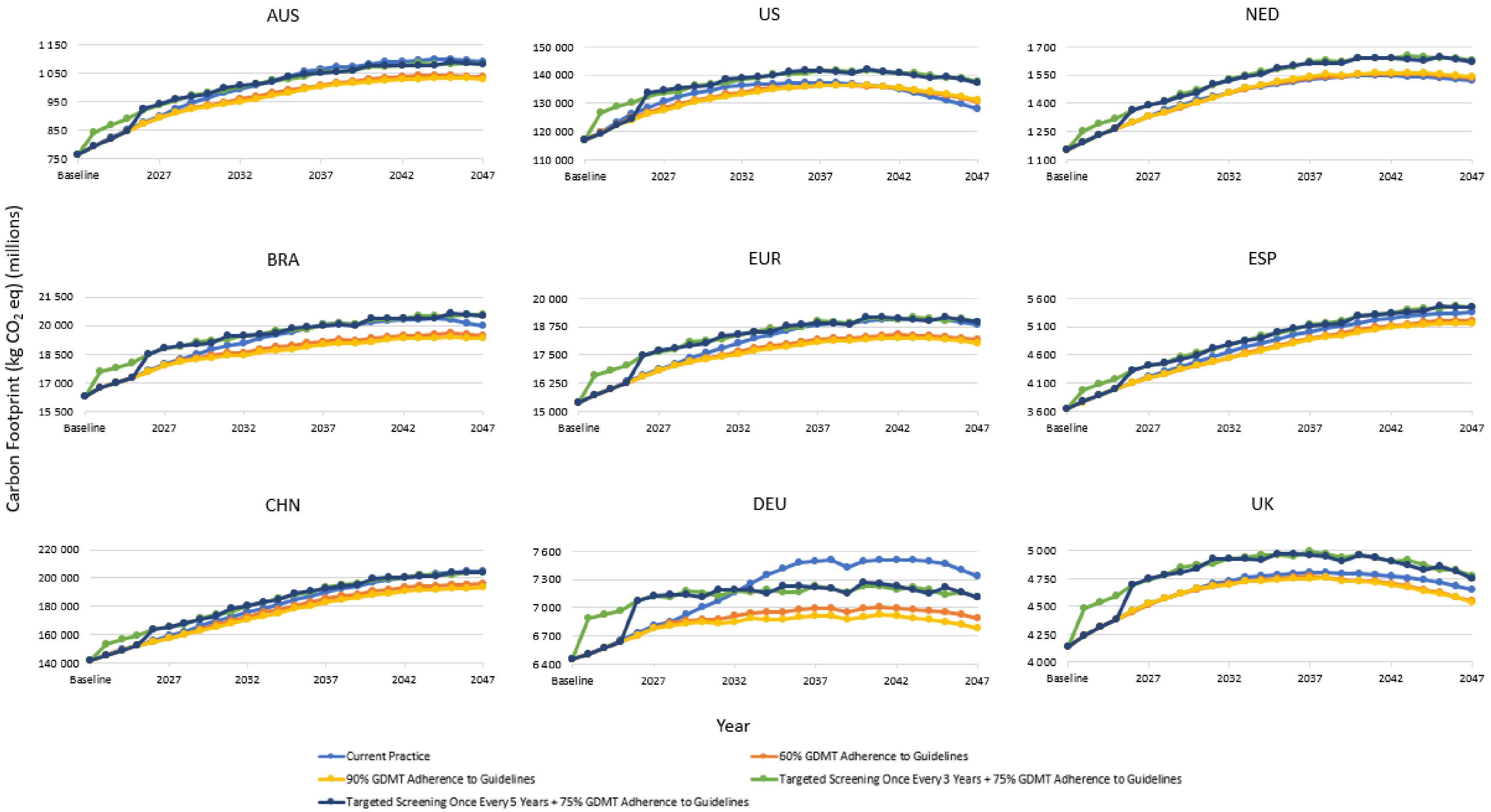

Abbreviations: AUS = Australia; BRA = Brazil; CHN = China; DEU = Germany; eq = equivalent; ESP = Spain; EUR = Europe; GDMT = guideline-directed medical therapy; KRT = kidney replacement therapy; NED = Netherlands; UK = United Kingdom; US = United States.

Supplementary References

S1. *Dapagliflozin*. PBS. https://www.pbs.gov.au/medicine/item/10011X-11291G-12823X-13106T
S2. *Irbesartan*. PBS. https://www.pbs.gov.au/medicine/item/13435D-8246B
S3. *Finerenone*. PBS. https://www.pbs.gov.au/medicine/item/13316W
S4. *Semaglutide*. PBS. https://www.pbs.gov.au/medicine/item/12080T
S5. *Simvastatin plus ezetimibe*. PBS. https://www.pbs.gov.au/medicine/item/13442L-9484E
S6. Evans M, Bain SC, Hogan S, Bilous RW, Collaborative Study Group p. Irbesartan delays progression of nephropathy as measured by estimated glomerular filtration rate: post hoc analysis of the Irbesartan Diabetic Nephropathy Trial. *Nephrol Dial Transplant*. Jun 2012;27(6):2255-63. doi:10.1093/ndt/gfr696
S7. *Drug Price Lists*. ANVISA. https://www.gov.br/anvisa/pt-br/assuntos/medicamentos/cmed/precos
S8. Tuttle KR, Bosch-Traberg H, Cherney DZI, et al. Post hoc analysis of SUSTAIN 6 and PIONEER 6 trials suggests that people with type 2 diabetes at high cardiovascular risk treated with semaglutide experience more stable kidney function compared with placebo. *Kidney Int*. Apr 2023;103(4):772-781. doi:10.1016/j.kint.2022.12.028
S9. Gu S, Mu Y, Zhai S, Zeng Y, Zhen X, Dong H. Cost-Effectiveness of Dapagliflozin versus Acarbose as a Monotherapy in Type 2 Diabetes in China. *PLoS One*. 2016;11(11):e0165629. doi:10.1371/journal.pone.0165629
S10. Annemans L, Demarteau N, Hu S, et al. An Asian regional analysis of cost-effectiveness of early irbesartan treatment versus conventional antihypertensive, late amlodipine, and late irbesartan treatments in patients with type 2 diabetes, hypertension, and nephropathy. *Value Health*. May-Jun 2008;11(3):354-64. doi:10.1111/j.1524-4733.2007.00250.x
S11. Bai G, Bennet C, Wang J, Anderson GF. Access to Antihypertensive Drugs in China. *Circulation*. Oct 23 2018;138(17):1777-1779. doi:10.1161/CIRCULATIONAHA.118.033360
S12. Ming J, Hong G, Xu Y, Mernagh P, Pochopien M, Li H. Cost-effectiveness of Finerenone in Addition to Standard of Care for Patients with Chronic Kidney Disease and Type 2 Diabetes in China. *Adv Ther*. Aug 2024;41(8):3138-3158. doi:10.1007/s12325-024-02906-w
S13. Feng Z, Tong WK, Zhang X, Tang Z. Cost-effectiveness analysis of once-daily oral semaglutide versus placebo and subcutaneous glucagon-like peptide-1 receptor agonists added to insulin in patients with type 2 diabetes in China. *Front Pharmacol*. 2023;14:1226778. doi:10.3389/fphar.2023.1226778
S14. Liu L, Ruan Z, Ung COL, et al. Long-Term Cost-Effectiveness of Subcutaneous Once-Weekly Semaglutide Versus Polyethylene Glycol Loxenatide for Treatment of Type 2 Diabetes Mellitus in China. *Diabetes Ther*. Jan 2023;14(1):93-107. doi:10.1007/s13300-022-01336-7
S15. Yang H, Li N, Zhou Y, et al. Cost-Effectiveness Analysis of Ezetimibe as the Add-on Treatment to Moderate-Dose Rosuvastatin versus High-Dose Rosuvastatin in the Secondary Prevention of Cardiovascular Diseases in China: A Markov Model Analysis. *Drug Des Devel Ther*. 2020;14:157-165. doi:10.2147/DDDT.S213968
S16. NAVLIN. CGM Lauer. https://portal.cgmlauer.cgm.com/LF/Seiten/Verwaltung/Kundencenter/1.aspx
S17. *List of Reference Prices for Medicinal Products*. BfArM. https://www.bfarm.de/EN/Medicinal-products/Information-on-medicinal-products/Reference-Pricing/_node.html
S18. Irbesartan EPAR
S19. Kerendia EPAR
S20. Green A. Kerendia. https://www.apotheke.green/online-shop/verf%C3%BCgbar%20lieferbar%20KERENDIA%2010%20mg%20Filmtabletten%20Apotheke
S21. *OZEMPIC 1 INJVLST 1.34MG/ML PEN 3ML + ACCESSORIES*. Dutch Healthcare Institute. https://www.medicijnkosten.nl/medicijn?artikel=OZEMPIC+1+INJVLST+1%2C34MG%2FML+PEN+3ML+%2B+TOEBEH&id=04581ca5fc12446c32c0d1eb0f348119
S22. *RYBELSUS TABLET 14MG*. Dutch Healthcare Institute. https://www.medicijnkosten.nl/medicijn?artikel=RYBELSUS+TABLET+14MG&id=0cfd3b1ef619a10dfbe313d9ad6281d4
S23. Baigent C, Landray MJ, Reith C, et al. The effects of lowering LDL cholesterol with simvastatin plus ezetimibe in patients with chronic kidney disease (Study of Heart and Renal Protection): a randomised placebo-controlled trial. *Lancet*. Jun 25 2011;377(9784):2181-92. doi:10.1016/S0140-6736(11)60739-3

S24. *FORXIGA TABLET FILM COVER 10MG*. Dutch Healthcare Institute. https://www.medicijnkosten.nl/medicijn?artikel=FORXIGA+TABLET+FILMOMHULD+10MG&id=87e2b5ca0568e9866b7a168becc03e19
S25. *IRBESARTAN XIROMED TABLET 75MG*. Dutch Healthcare Institute. https://www.medicijnkosten.nl/medicijn?artikel=IRBESARTAN+XIROMED+TABLET+75MG&id=39cba003f4d839bef7b65fe9ce653471
S26. *IRBESARTAN XIROMED TABLET 150MG*. Dutch Healthcare Institute. https://www.medicijnkosten.nl/medicijn?artikel=IRBESARTAN+XIROMED+TABLET+150MG&id=1f0657223d6fe3773e72d026c23d1391
S27. *IRBESARTAN AUROBINDO TABLET 300MG*. Dutch Healthcare Institute. https://www.medicijnkosten.nl/medicijn?artikel=IRBESARTAN+AUROBINDO+TABLET+300MG&id=94a8c5a342c1263237d78b7a2212ad15
S28. *KERENDIA TABLET FILM COVER 20MG*. Dutch Healthcare Institute. https://www.medicijnkosten.nl/medicijn?artikel=KERENDIA+TABLET+FILMOMHULD+20MG&id=9f056d6a4ce16387b3415f81f1161ecd
S29. *EZETIMIBE/SIMVASTATINE KRKA TABLET 10/20MG*. Dutch Healthcare Institute. https://www.medicijnkosten.nl/medicijn?artikel=EZETIMIBE%2FSIMVASTATINE+KRKA+TABLET+10%2F20MG&id=087d6ec5419860bda792bd543bfdd450
S30. *nformation on products included in the pharmaceutical benefits of the SNS*. Spanish Ministry of Health. https://www.sanidad.gob.es/profesionales/nomenclator.do?metodo=buscarProductos
S31. Dapagliflozin for treating chronic kidney disease (2022).
S32. BNF. *BNF 78*. BNF; 2020.
S33. Finerenone for treating chronic kidney disease in type 2 diabetes (2023).
S34. New Medicine Assessment - Semaglutide Oral Tablets (Rybelsus®) (2020).
S35. Haynes R, Lewis D, Emberson J, et al. Effects of lowering LDL cholesterol on progression of kidney disease. *J Am Soc Nephrol*. Aug 2014;25(8):1825-33. doi:10.1681/ASN.2013090965
S36. Physician AF. Dapagliflozin (Farxiga) for Type 2 Diabetes Mellitus. Updated June 15, 2015. https://www.aafp.org/pubs/afp/issues/2015/0615/p828.html
S37. Palmer AJ, Valentine WJ, Chen R, et al. A health economic analysis of screening and optimal treatment of nephropathy in patients with type 2 diabetes and hypertension in the USA. *Nephrol Dial Transplant*. Apr 2008;23(4):1216-23. doi:10.1093/ndt/gfn082
S38. Dayer V, Hansen R, Singh R, Kong S, Williamson T, Sullivan S. Potential Budget Impact of Finerenone in Patients with Chronic Kidney Disease and Type 2 Diabetes Being Treated with Standard of Care. *Value in Health*. 2022;25(7):S413.
S39. Hunt B, Hansen BB, Ericsson A, et al. Evaluation of the Cost Per Patient Achieving Treatment Targets with Oral Semaglutide: A Short-Term Cost-Effectiveness Analysis in the United States. *Adv Ther*. Dec 2019;36(12):3483-3493. doi:10.1007/s12325-019-01125-y
S40. Mody RR, Meyer KL, Ward JM, O'Day KB. Cost per Patient Achieving Treatment Targets and Number Needed to Treat with Tirzepatide Versus Semaglutide 1 mg in Patients with Type 2 Diabetes in the United States. *Diabetes Ther*. Dec 2023;14(12):2045-2055. doi:10.1007/s13300-023-01470-w
S41. Schlackow I, Kent S, Herrington W, et al. Cost-effectiveness of lipid lowering with statins and ezetimibe in chronic kidney disease. *Kidney Int*. Jul 2019;96(1):170-179. doi:10.1016/j.kint.2019.01.028
S42. Ruilope LM, Pitt B, Anker SD, et al. Kidney outcomes with finerenone: an analysis from the FIGARO-DKD study. *Nephrol Dial Transplant*. Feb 13 2023;38(2):372-383. doi:10.1093/ndt/gfac157
S43. Heerspink HJL, Stefansson BV, Correa-Rotter R, et al. Dapagliflozin in Patients with Chronic Kidney Disease. *N Engl J Med*. Oct 8 2020;383(15):1436-1446. doi:10.1056/NEJMoa2024816
S44. Robinson-Cohen C, Littman AJ, Duncan GE, et al. Physical activity and change in estimated GFR among persons with CKD. *J Am Soc Nephrol*. Feb 2014;25(2):399-406. doi:10.1681/ASN.2013040392
S45. Agarwal R, Filippatos G, Pitt B, et al. Cardiovascular and kidney outcomes with finerenone in patients with type 2 diabetes and chronic kidney disease: the FIDELITY pooled analysis. *Eur Heart J*. Feb 10 2022;43(6):474-484. doi:10.1093/eurheartj/ehab777
S46. Staplin N, Roddick AJ, Emberson J, et al. Net effects of sodium-glucose co-transporter-2 inhibition in different patient groups: a meta-analysis of large placebo-controlled randomized trials. *EClinicalMedicine*. Nov 2021;41:101163. doi:10.1016/j.eclinm.2021.101163

S47. Kelly M, Lewis J, Rao H, Carter J, Portillo I, Beuttler R. Effects of GLP-1 receptor agonists on cardiovascular outcomes in patients with type 2 diabetes and chronic kidney disease: A systematic review and meta-analysis. *Pharmacotherapy*. Dec 2022;42(12):921-928. doi:10.1002/phar.2737
S48. Tunnicliffe DJ, Palmer SC, Cashmore BA, et al. HMG CoA reductase inhibitors (statins) for people with chronic kidney disease not requiring dialysis. *Cochrane Database Syst Rev*. Nov 29 2023;11(11):CD007784. doi:10.1002/14651858.CD007784.pub3
S49. Brenner BM, Cooper ME, de Zeeuw D, et al. Effects of losartan on kidney and cardiovascular outcomes in patients with type 2 diabetes and nephropathy. *N Engl J Med*. Sep 20 2001;345(12):861-9. doi:10.1056/NEJMoa011161
S50. Berl T, Hunsicker LG, Lewis JB, et al. Cardiovascular outcomes in the Irbesartan Diabetic Nephropathy Trial of patients with type 2 diabetes and overt nephropathy. *Ann Intern Med*. Apr 1 2003;138(7):542-9. doi:10.7326/0003-4819-138-7-200304010-00010
S51. Mavrakanas TA, Tsoukas MA, Brophy JM, Sharma A, Gariani K. SGLT-2 inhibitors improve cardiovascular and kidney outcomes in patients with CKD: a systematic review and meta-analysis. *Sci Rep*. Sep 23 2023;13(1):15922. doi:10.1038/s41598-023-42989-z
S52. Yang S, He W, Zhao L, Mi Y. Association between use of sodium-glucose cotransporter 2 inhibitors, glucagon-like peptide 1 agonists, and dipeptidyl peptidase 4 inhibitors with kidney outcomes in patients with type 2 diabetes: A systematic review and network meta-analysis. *PLoS One*. 2022;17(4):e0267025. doi:10.1371/journal.pone.0267025

CHEERS 2022 Checklist

Topic	No.	Item	Location where item is reported
**Title**			
	1	Identify the study as an economic evaluation and specify the interventions being compared.	Page 1; Note: manuscript reports on a mathematical model that evaluates multiple aspects of CKD including epidemiology, economics, environmental impact. As such, "economic evaluation" is not stated in title.
**Abstract**			
	2	Provide a structured summary that highlights context, key methods, results, and alternative analyses.	Page 2
**Introduction**			
**Background and objectives**	3	Give the context for the study, the study question, and its practical relevance for decision making in policy or practice.	Page 4-5
**Methods**			
**Health economic analysis plan**	4	Indicate whether a health economic analysis plan was developed and where available.	Not applicable
**Study population**	5	Describe characteristics of the study population (such as age range, demographics, socioeconomic, or clinical characteristics).	Page 5

Topic	No.	Item	Location where item is reported
**Setting and location**	6	Provide relevant contextual information that may influence findings.	Page 5
**Comparators**	7	Describe the interventions or strategies being compared and why chosen.	Page 5-6; Figure 1
**Perspective**	8	State the perspective(s) adopted by the study and why chosen.	Page 5
**Time horizon**	9	State the time horizon for the study and why appropriate.	Page 5
**Discount rate**	10	Report the discount rate(s) and reason chosen.	Not applicable
**Selection of outcomes**	11	Describe what outcomes were used as the measure(s) of benefit(s) and harm(s).	Published methodology manuscript referenced on page 4 and 5: Brown et al, 2024
**Measurement of outcomes**	12	Describe how outcomes used to capture benefit(s) and harm(s) were measured.	Published methodology manuscript referenced on page 4 and 5: Brown et al, 2024
**Valuation of outcomes**	13	Describe the population and methods used to measure and value outcomes.	Published methodology manuscript referenced on page 4 and 5: Brown et al, 2024
**Measurement and valuation of resources and costs**	14	Describe how costs were valued.	Supplementary Table S1-S8

Topic	No.	Item	Location where item is reported
**Currency, price date, and conversion**	15	Report the dates of the estimated resource quantities and unit costs, plus the currency and year of conversion.	Page 6; Supplementary Table S1-S8
**Rationale and description of model**	16	If modelling is used, describe in detail and why used. Report if the model is publicly available and where it can be accessed.	Page 5; Published methodology manuscript referenced on page 4 and 5: Brown et al, 2024
**Analytics and assumptions**	17	Describe any methods for analysing or statistically transforming data, any extrapolation methods, and approaches for validating any model used.	For analyses: Page 6 and Figure 1; For epidemiological model: Published methodology manuscript referenced on page 4 and 5 - Brown et al, 2024
**Characterising heterogeneity**	18	Describe any methods used for estimating how the results of the study vary for subgroups.	Not applicable
**Characterising distributional effects**	19	Describe how impacts are distributed across different individuals or adjustments made to reflect priority populations.	Not applicable
**Characterising uncertainty**	20	Describe methods to characterise any sources of uncertainty in the analysis.	Sensitivity analyses: Page 7; Supplementary Materials Page 3
**Approach to engagement with patients and others affected by the study**	21	Describe any approaches to engage patients or service recipients, the general public, communities, or stakeholders (such as clinicians or payers) in the design of the study.	Not applicable

Topic	No.	Item	Location where item is reported
**Results**			
**Study parameters**	22	Report all analytic inputs (such as values, ranges, references) including uncertainty or distributional assumptions.	Table 1, Supplementary Table S1-S8 and Supplementary Table S9-S10; Country epidemiological inputs informing model reported in manuscript (Rao et al) currently under review at Kidney International Reports.
**Summary of main results**	23	Report the mean values for the main categories of costs and outcomes of interest and summarise them in the most appropriate overall measure.	Page 7-11; Figures 2-6; Supplementary Figures S1-S5; Supplementary Tables S11-S18;
**Effect of uncertainty**	24	Describe how uncertainty about analytic judgments, inputs, or projections affect findings. Report the effect of choice of discount rate and time horizon, if applicable.	Sensitivity analyses: Page 9 and Page 11; Supplementary Figures S9-S21
**Effect of engagement with patients and others affected by the study**	25	Report on any difference patient/service recipient, general public, community, or stakeholder involvement made to the approach or findings of the study	Not applicable
**Discussion**			
**Study findings, limitations, generalisability, and current knowledge**	26	Report key findings, limitations, ethical or equity considerations not captured, and how these could affect patients, policy, or practice.	Page 11-15
**Other relevant information**			

Topic	No.	Item	Location where item is reported
**Source of funding**	27	Describe how the study was funded and any role of the funder in the identification, design, conduct, and reporting of the analysis	Page 16
**Conflicts of interest**	28	Report authors conflicts of interest according to journal or International Committee of Medical Journal Editors requirements.	Page 16; ICMJE forms attached

From: Husereau D, Drummond M, Augustovski F, et al. Consolidated Health Economic Evaluation Reporting Standards 2022 (CHEERS 2022) Explanation and Elaboration: A Report of the ISPOR CHEERS II Good Practices Task Force. Value Health 2022;25. doi:10.1016/j.jval.2021.10.008
